# Supplementary material for: Coping with the COVID-19 Pandemic: Perceived Changes in Psychological Vulnerability, Resilience and Social Cohesion before, during and after Lockdown
Source: Int J Environ Res Public Health. 2022 Mar 10;19(6):3290. doi: 10.3390/ijerph19063290 (PMC8952288; doi:10.3390/ijerph19063290)
Supplement: Supplementary file 1 [file ijerph-19-03290-s001.zip › supplement S1 appendix B.pdf]

## Appendix B

**Table B1. The table below provides an overview of the descriptive statistics of the measures of state vulnerability, resilience, and social cohesion.**

| Questionnaire | Questions | Items | T1 | T2 | T3 | Summary type |
|---|---|---|---|---|---|---|
| Life satisfaction | How satisfied were you with your life? | | 5.77 (5.71/5.82) | 4.66 (4.60/4.73) | 5.41 (5.35/5.46) | Mean & 95% CI |
| Emotional state | My emotional state was characterized by.. | Valence | 1.23 (1.15/1.30) | -0.77 (-0.84/-0.69) | 0.93 (0.86/1.00) | Mean & 95% CI |
| | | Arousal | -0.77 (-0.84/-0.70) | -0.27 (-0.34/-0.20) | -0.51 (-0.58/-0.45) | Mean & 95% CI |
| Place of residence | Did you live at your usual place of residence? | Yes | 3366 | 3312 | 3392 | Total Numbers |
| | | No | 156 | 210 | 130 | Total Numbers |
| Household | With how many people did you live together? | Alone | 879 | 817 | 878 | Total Numbers |
| | | 1 | 1139 | 1285 | 1329 | Total Numbers |
| | | 2 | 707 | 622 | 581 | Total Numbers |
| | | 3 | 449 | 460 | 437 | Total Numbers |
| | | 4 | 232 | 218 | 195 | Total Numbers |
| | | 5 or more | 116 | 120 | 102 | Total Numbers |
| | Which of these people have you lived with? | Partner | 2041 | 2121 | 2092 | Total Numbers |
| | | Parents | 232 | 295 | 234 | Total Numbers |
| | | Children | 1079 | 1118 | 1093 | Total Numbers |
| | | Friends / Housemates | 362 | 305 | 303 | Total Numbers |
| | How many of them required care? | None | 2495 | 2541 | 2470 | Total Numbers |
| | | 1 | 116 | 120 | 132 | Total Numbers |
| | | 2 | 23 | 33 | 25 | Total Numbers |
| | | 3 | 7 | 9 | 11 | Total Numbers |
| | | 4 | 1 | 1 | 4 | Total Numbers |
| | | 5 or more | 3 | 1 | 3 | Total Numbers |
| Safety measures | Please evaluate to what extent you agree with the following statements | I followed the regulations. | 6.94 (6.90/6.98) | 7.13 (7.09/7.17) | 6.84 (6.80/6.89) | Mean & 95% CI |
| | | The regulations for the protection of the society were overall too strict. | 1.79 (1.72/1.86) | 2.10 (2.02/2.18) | 1.97 (1.90/2.04) | Mean & 95% CI |
| | | The regulations for the protection of the society were overall too weak. | 2.86 (2.78/2.94) | 2.56 (2.48/2.64) | 3.10 (3.02/3.19) | Mean & 95% CI |
| | | The people in my environment adhered too much to the regulations. | 2.54 (2.46/2.62) | 3.02 (2.93/3.11) | 2.66 (2.58/2.74) | Mean & 95% CI |
| | | The people in my environment did not adhere enough to the regulations. | 2.25 (2.18/2.32) | 2.28 (2.21/2.35) | 2.81 (2.73/2.88) | Mean & 95% CI |
| Optimism | How optimistic were you? | | 5.69 (5.64/5.75) | 4.33 (4.27/4.40) | 5.03 (4.97/5.09) | Mean & 95% CI |
| Alcohol consumption | AUDIT | | 2.65 (2.58/2.72) | 2.52 (2.45/2.60) | 2.58 (2.51/2.66) | Mean & 95% CI |
| | Did you feel as if your alcohol consumption has gotten out of control? | | 0.43 (0.40/0.47) | 0.76 (0.71/0.81) | 0.61 (0.56/0.65) | Mean & 95% CI |
| News | How often did you consume news and information concerning current negative events? | | 5.04 (4.97/5.11) | 6.75 (6.69/6.80) | 5.59 (5.52/5.65) | Mean & 95% CI |
| Mobility | How often did you spend time outdoors in your spare time? | | 5.52 (5.45/5.58) | 5.54 (5.47/5.62) | 6.42 (6.37/6.48) | Mean & 95% CI |
| Stress | How stressed were you? | | 4.00 (3.93/4.06) | 4.59 (4.51/4.66) | 3.91 (3.84/3.98) | Mean & 95% CI |
| Engagement | How much did you participate in social / political engagement? | Social | 3.93 (3.85/4.02) | 1.29 (1.23/1.35) | 2.94 (2.86/3.03) | Mean & 95% CI |
| | | Political | 2.95 (2.87/3.03) | 1.75 (1.68/1.82) | 1.98 (1.91/2.05) | Mean & 95% CI |
| Belonging | Please move the circles so that they best represent your belonging to the following groups | Me and family | 79.45 (78.60/80.29) | 76.88 (75.93/77.83) | 80.04 (79.19/80.89) | Mean & 95% CI |
| | | Me and friends | 75.54 (74.77/76.36) | 62.89 (61.90/63.88) | 73.28 (72.46/74.10) | Mean & 95% CI |
| | | Me and neighbours | 45.66 (44.66/46.66) | 39.35 (38.30/40.40) | 42.68 (41.68/43.73) | Mean & 95% CI |
| | | Me and population of Berlin | 41.90 (40.91/42.88) | 32.13 (31.14/33.12) | 36.9 (35.9/37.9) | Mean & 95% CI |
| | | Me and German population | 37.43 (36.42/38.42) | 29.90 (28.89/30.90) | 31.81 (30.82/32.81) | Mean & 95% CI |
| | | Me and European population | 37.69 (36.66/38.73) | 30.93 (29.90/31.97) | 32.51 (31.48/33.53) | Mean & 95% CI |

| | | | | | | | |
|---|---|---|---|---|---|---|---|
| | | Me and world population | | 33.33 (32.29/34.38) | 28.56 (27.53/31.97) | 29.56 (28.54/30.59) | Mean & 95% CI |
| Trust | How strongly did you trust in.. | Family | | 6.98 (6.92/7.03) | 7.02 (6.97/7.08) | 7.05 (6.99/7.10) | Mean & 95% CI |
| | | Friends | | 6.70 (6.65/6.75) | 6.60 (6.54/6.65) | 6.70 (6.65/6.75) | Mean & 95% CI |
| | | Neighbours | | 4.10 (4.03/4.16) | 4.07 (4.00/4.14) | 3.97 (3.90/4.04) | Mean & 95% CI |
| | | Fellow citizens | | 3.32 (3.27/3.38) | 3.14 (3.08/3.19) | 3.00 (2.95/3.06) | Mean & 95% CI |
| | | German public-legal media | | 5.24 (5.17/5.31) | 5.45 (5.37/5.52) | 5.32 (5.24/5.39) | Mean & 95% CI |
| | | German police | | 5.29 (5.22/5.35) | 5.37 (5.30/5.44) | 5.26 (5.19/5.33) | Mean & 95% CI |
| | | Berlin Senat | | 4.12 (4.06/4.18) | 4.37 (4.30/4.44) | 4.27 (4.19/4.34) | Mean & 95% CI |
| | | German Chancellor | | 5.37 (5.30/5.44) | 5.63 (5.55/5.70) | 5.46 (5.38/5.53) | Mean & 95% CI |
| | | German government | | 4.72 (4.66/4.79) | 5.09 (5.02/5.16) | 4.87 (4.80/4.94) | Mean & 95% CI |
| | | German health system | | 5.88 (5.82/5.94) | 6.01 (5.95/6.07) | 6.06 (6.00/6.12) | Mean & 95% CI |
| | | Science | | 6.62 (6.57/6.67) | 6.63 (6.58/6.69) | 6.61 (6.55/6.66) | Mean & 95% CI |
| Aggression | How often have you been verbally or physically aggressive towards … | Partner | | 0.73 (0.68/0.77) | 0.88 (0.83/0.93) | 0.60 (0.56/0.63) | Mean & 95% CI |
| | | Family members | | 0.78 (0.74/0.82) | 0.84 (0.79/0.88) | 0.59 (0.56/0.63) | Mean & 95% CI |
| | | Friends | | 0.45 (0.42/0.48) | 0.47 (0.44/0.51) | 0.38 (0.35/0.41) | Mean & 95% CI |
| | | Neighbours | | 0.32 (0.29/0.35) | 0.31 (0.28/0.34) | 0.28 (0.25/0.30) | Mean & 95% CI |
| | | Colleagues | | 0.60 (0.56/0.63) | 0.50 (0.46/0.53) | 0.44 (0.40/0.47) | Mean & 95% CI |
| | | Superiors | | 0.45 (0.42/0.49) | 0.40 (0.36/0.43) | 0.36 (0.33/0.39) | Mean & 95% CI |
| | | Strangers / other individuals | | 0.89 (0.85/0.93) | 0.88 (0.84/0.93) | 0.72 (0.68/0.76) | Mean & 95% CI |
| | How often have you been a victim of verbal or physical aggression by … | Partner | | 0.55 (0.51/0.59) | 0.65 (0.61/0.70) | 0.50 (0.47/0.54) | Mean & 95% CI |
| | | Family members | | 0.54 (0.50/0.57) | 0.58 (0.54/0.62) | 0.48 (0.45/0.52) | Mean & 95% CI |
| | | Friends | | 0.28 (0.26/0.30) | 0.33 (0.30/0.36) | 0.29 (0.26/0.32) | Mean & 95% CI |
| | | Neighbours | | 0.30 (0.28/0.33) | 0.30 (0.28/0.33) | 0.28 (0.26/0.31) | Mean & 95% CI |
| | | Colleagues | | 0.52 (0.49/0.56) | 0.45 (0.42/0.49) | 0.43 (0.39/0.46) | Mean & 95% CI |
| | | Superiors | | 0.50 (0.47/0.54) | 0.42 (0.38/0.46) | 0.40 (0.37/0.44) | Mean & 95% CI |
| | | Strangers / other individuals | | 1.15 (1.10/1.20) | 1.11 (1.06/1.17) | 0.98 (0.93/1.03) | Mean & 95% CI |
| Children in household | How many of the people in your household were children (younger than 18)? | | None | 2556 | 2549 | 2563 | Total Numbers |
| | | | 1 | 495 | 496 | 492 | Total Numbers |
| | | | 2 | 376 | 375 | 369 | Total Numbers |
| | | | 3 | 79 | 81 | 79 | Total Numbers |
| | | | 4 | 13 | 16 | 15 | Total Numbers |
| | | | 5 or more | 3 | 5 | 4 | Total Numbers |
| | Were you a single parent? | | Yes | 138 | 133 | 136 | Total Numbers |
| | | | No | 833 | 841 | 882 | Total Numbers |
| | Has it happened in your household that a child has been a witness or victim of verbally or physically aggressive behaviour, or that it has been neglected or overwhelmed? | | Yes | 136 | 155 | 77 | Total Numbers |
| | | | No | 831 | 819 | 882 | Total Numbers |
| | How often has at least one adult living (/ who previously lived) in your household with your child threatened to leave you or your child? (or has actually done it?) | | | 0.41 (0.22/0.59) | 0.55 (0.34/0.77) | 0.64 (0.32/0.96) | Mean & 95% CI |

| | | | | | | |
|---|---|---|---|---|---|---|
| Aggression children | How often has it occurred that a child in your household had to take on the responsibility of a significantly older child or adult? | | 0.71 (0.45/0.98) | 1.06 (0.77/1.34) | 0.71 (0.39/1.04) | Mean & 95% CI |
| | How often has an adult locked in a child of your household in a closet, attic, basement, garage, or another possibly very narrow, dark place? | | 0 (0/0) | 0.02 (0/0.04) | 0.01 (-0.01/0.04) | Mean & 95% CI |
| | How often did it occur that at least one parent (or other main caregiver / parent-like person) failed to understand the feelings of a child in your household and to be there for them? | | 1.83 (1.53/2.13) | 2.64 (2.32/2.96) | 2.06 (1.66/2.47) | Mean & 95% CI |
| | How often has at least one adult in your household purposely pushed, pinched, slapped, punched, or kicked a child in your household? | | 0.28 (0.16/0.41) | 0.35 (0.22/0.49) | 0.32 (0.18/0.46) | Mean & 95% CI |
| | How often has at least one adult in your household purposely pushed, pinched, slapped, punched, or kicked a child in your household? | | 0.76 (0.51/1.02) | 1.37 (1.07/1.67) | 1.05 (0.70/1.40) | Mean & 95% CI |
| | How often has a child in your household witnessed adults living in your household arguing violently with each other? | | 1.76 (1.44/2.09) | 2.47 (2.11/2.83) | 1.79 (1.38/2.21) | Mean & 95% CI |
| | How often has a child in your household seen adults beat their mother / father or another adult in the household so badly that they were severely injured? | | 0.07 (-0.02/0.15) | 0.06 (-0.03/0.16) | 0.01 (-0.01/0.04) | Mean & 95% CI |
| Income | Were you gainfully employed? | Yes | 2893 | 2675 | 2768 | Total Numbers |
| | | No | 629 | 847 | 754 | Total Numbers |
| Occupation | How many hours did you work on average per week | | 36.17 (35.75/36.58) | 33.44 (32.93/33.96) | 34.66 (34.20/35.12) | Mean & 95% CI |
| | How high was your workload? | | 5.85 (5.79/5.91) | 5.29 (5.21/5.38) | 5.49 (5.41/5.56) | Mean & 95% CI |
| | What percentage of your working time did you spend in home office | | 12.82 (11.92/13.71) | 54.31 (52.64/55.99) | 37.24 (35.74/38.74) | Mean & 95% CI |
| | What percentage of your working time did you spend at your usual workplace? | | 68.68 (67.23/70.12) | 35.98 (34.38/37.58) | 51.82 (50.25/53.39) | Mean & 95% CI |
| Financial | How would you rate your financial security? | | 5.62 (5.55/5.69) | 5.57 (5.50/5.64) | 5.80 (5.73/5.86) | Mean & 95% CI |
| | How difficult was it, to cover basic needs? (e.g. groceries, rent etc.)? | | 1.01 (0.96/1.07) | 1.14 (1.08/1.20) | 0.83 (0.78/0.88) | Mean & 95% CI |
| Contact | How often did you have contact with… | Partner | 3.74 (3.71/3.76) | 3.68 (3.64/3.71) | 3.72 (3.70/3.75) | Mean & 95% CI |
| | | Family members | 2.60 (2.56/2.64) | 1.87 (1.82/1.92) | 2.47 (2.43/2.52) | Mean & 95% CI |
| | | Friends | 2.50 (2.47/2.53) | 1.09 (1.05/1.12) | 2.24 (2.21/2.28) | Mean & 95% CI |
| | | Colleagues | 3.17 (3.13/3.21) | 1.55 (1.50/1.61) | 2.20 (2.15/2.25) | Mean & 95% CI |
| | | Superiors | 2.65 (2.61/2.70) | 1.24 (1.19/1.29) | 1.72 (1.67/1.77) | Mean & 95% CI |
| | | Other individuals | 2.19 (2.16/2.23) | 1.02 (0.99/1.05) | 1.51 (1.48/1.55) | Mean & 95% CI |
| | On average, how pleasant were the personal interactions with the following people? | Partner | 2.33 (2.27/2.39) | 2.20 (2.14/2.27) | 3.25 (3.20/3.30) | Mean & 95% CI |
| | | Family members | 2.62 (2.57/2.68) | 2.00 (1.94/2.06) | 2.77 (2.72/2.82) | Mean & 95% CI |
| | | Friends | 2.97 (2.93/3.01) | 1.85 (1.79/1.91) | 2.93 (2.89/2.98) | Mean & 95% CI |
| | | Colleagues | 1.57 (1.52/1.63) | 0.84 (0.79/0.90) | 1.61 (1.55/1.67) | Mean & 95% CI |
| | | Superiors | 0.95 (0.89/1.01) | 0.43 (0.38/0.48) | 0.99 (0.92/1.05) | Mean & 95% CI |
| | | Other individuals | 0.83 (0.78/0.88) | 0.11 (0.06/0.15) | 0.47 (0.42/0.52) | Mean & 95% CI |
| Online contact | How often did you have online contact with … | Partner | 2.24 (2.18/2.29) | 1.91 (1.86/1.97) | 2.17 (2.11/2.22) | Mean & 95% CI |
| | | Family members | 2.35 (2.32/2.39) | 2.55 (2.51/2.59) | 2.44 (2.40/2.48) | Mean & 95% CI |
| | | Friends | 2.50 (2.46/2.53) | 2.68 (2.65/2.72) | 2.51 (2.48/2.54) | Mean & 95% CI |
| | | Colleagues | 1.82 (1.78/1.87) | 2.23 (2.18/2.27) | 2.07 (2.02/2.11) | Mean & 95% CI |
| | | Superiors | 1.45 (1.40/1.49) | 1.83 (1.78/1.88) | 1.66 (1.61/1.70) | Mean & 95% CI |
| | | Other individuals | 1.30 (1.27/1.34) | 1.18 (1.15/1.22) | 1.15 (1.12/1.19) | Mean & 95% CI |
| | | Partner | 1.84 (1.77/1.90) | 1.66 (1.60/1.73) | 2.62 (2.55/2.68) | Mean & 95% CI |
| | | Family members | 2.43 (2.38/2.49) | 2.51 (2.46/2.56) | 2.66 (2.61/2.71) | Mean & 95% CI |

| | | | | | | |
|---|---|---|---|---|---|---|
| | On average, how pleasant were the personal interactions with the following people? | Friends | 2.70 (2.66/2.75) | 2.68 (2.63/2.73) | 2.81 (2.77/2.85) | Mean & 95% CI |
| | | Colleagues | 1.23 (1.18/1.28) | 1.27 (1.22/1.32) | 1.56 (1.50/1.61) | Mean & 95% CI |
| | | Superiors | 0.66 (0.61/0.71) | 0.71 (0.66/0.77) | 0.98 (0.92/1.04) | Mean & 95% CI |
| | | Other individuals | 0.66 (0.62/0.70) | 0.46 (0.41/0.50) | 0.58 (0.53/0.62) | Mean & 95% CI |
| Burden | How burdened did you feel by the following factors? | Negative political events and media coverage | 3.19 (3.12/3.26) | 5.11 (5.04/5.18) | 4.30 (4.22/4.37) | Mean & 95% CI |
| | | Financial or economic problems | 2.00 (1.93/2.07) | 2.41 (2.32/2.49) | 2.14 (2.07/2.22) | Mean & 95% CI |
| | | Loss of job or insolvency of a private company for yourself or someone in your household | 0.87 (0.81/0.93) | 1.59 (1.51/1.67) | 1.33 (1.26/1.41) | Mean & 95% CI |
| | | Problems with the supply of basic needs or the procurement of goods and services | 0.84 (0.79/0.90) | 2.11 (2.04/2.19) | 0.90 (0.84/0.95) | Mean & 95% CI |
| | | Personal physical health problems | 2.25 (2.18/2.33) | 2.10 (2.02/20.18) | 1.95 (1.87/2.03) | Mean & 95% CI |
| | | Physical health problems of friends, family members or colleagues | 2.40 (2.32/2.47) | 2.54 (2.46/2.63) | 2.28 (2.20/2.35) | Mean & 95% CI |
| | | Personal mental health problems | 1.77 (1.69/1.84)) | 2.33 (2.24/2.41) | 1.94 (1.86/2.02) | Mean & 95% CI |
| | | Mental health problems of friends, family members or colleagues | 1.88 (1.81/1.95) | 2.36 (2.28/2.44) | 2.03 (1.96/2.10) | Mean & 95% CI |
| | | Personal increased risk of catching an infectious disease | 1.06 (0.99/1.12) | 2.23 (2.14/2.31) | 1.94 (1.86/2.02) | Mean & 95% CI |
| | | Increased risk of an infectious disease among friends, family members or colleagues | 1.52 (1.45/1.59) | 3.63 (3.54/3.71) | 2.93 (2.85/3.02) | Mean & 95% CI |
| | | Problems concerning childcare, difficulties in coordinating childcare and work | 0.56 (0.51/0.61) | 1.26 (1.17/1.34) | 0.77 (0.71/0.83) | Mean & 95% CI |
| | | Tension or conflicts at home or family conflicts | 1.58 (1.51/1.65) | 2.00 (1.92/2.08) | 1.50 (1.43/1.57) | Mean & 95% CI |
| | | Tension or conflicts with friends and / or acquaintances | 0.94 (0.90/0.99) | 1.18 (1.12/1.24) | 0.95 (0.89/1.00) | Mean & 95% CI |
| | | Stressful experiences (e.g. conflicts) at work, university or other occupation | 2.06 (1.99/2.13) | 2.12 (2.04/2.20) | 1.80 (1.73/1.88) | Mean & 95% CI |
| | | Increased workload | 3.13 (3.04/3.21) | 3.12 (3.02/3.22) | 3.07 (2.98/3.16) | Mean & 95% CI |
| | | Housing conditions such as tight living space (e.g. no access to a private outdoor area, too many roommates) | 0.97 (0.91/1.04) | 1.49 (1.41/1.57) | 1.09 (1.03/1.16) | Mean & 95% CI |
| | | Loss of a loved one through separation or death | 0.87 (0.80/0.93) | 0.86 (0.80/0.93) | 0.79 (0.72/0.85) | Mean & 95% CI |
| | | Limitations of social contacts and / or opportunities to participate in social events | 1.13 (1.06/1.19) | 4.71 (4.63/4.79) | 3.16 (3.09/3.24) | Mean & 95% CI |
| | | Limited travel options (private or business) | 0.97 (0.91/1.04) | 4.52 (4.43/4.61) | 3.76 (3.67/3.85) | Mean & 95% CI |
| | | Limited leisure opportunities and / or little exercise | 1.38 (1.31/1.44) | 4.71 (4.63/4.79) | 3.35 (3.27/3.43) | Mean & 95% CI |
| | | Discrimination based on your migration status | 0.20 (0.17/0.23) | 0.18 (0.15/0.21) | 0.14 (0.12/0.17) | Mean & 95% CI |
| | | Discrimination based on your sexual orientation | 0.18 (0.15/0.20) | 0.12 (0.09/0.14) | 0.12 (0.10/0.15) | Mean & 95% CI |
| | | Discrimination based on your gender | 0.60 (0.55/0.64) | 0.35 (0.32/0.39) | 0.35 (0.31/0.38) | Mean & 95% CI |
| | | Other reasons | 0.80 (0.74/0.85) | 1.11 (1.04/1.18) | 0.98 (0.94/1.04) | Mean & 95% CI |

| | | | | | | |
|---|---|---|---|---|---|---|
| Prosocial | How many times have the following people offered you help or have done something for you? | Partner | 3.06 (3.03/3.10) | 3.08 (3.05/3.12) | 3.05 (3.02/3.09) | Mean & 95% CI |
| | | Family members | 2.34 (2.30/2.37) | 2.27 (2.23/2.31) | 2.34 (2.31/2.38) | Mean & 95% CI |
| | | Friends | 2.11 (2.08/2.15) | 1.89 (1.85/1.92) | 2.05 (2.01/2.09) | Mean & 95% CI |
| | | Neighbours | 0.96 (0.93/0.99) | 0.91 (0.87/0.94) | 0.87 (0.83/0.90) | Mean & 95% CI |
| | | Colleagues | 1.60 (1.56/1.64) | 1.34 (1.30/1.38) | 1.38 (1.34/1.42) | Mean & 95% CI |
| | | Superiors | 1.17 (1.13/1.21) | 1.04 (1.00/1.08) | 1.02 (0.98/1.06) | Mean & 95% CI |
| | | Strangers/Other individuals | 0.67 (0.64/0.69) | 0.46 (0.44/0.49) | 0.52 (0.50/0.55) | Mean & 95% CI |
| | How often have you offered your help or have done something for the following people? | Partner | 3.25 (3.22/3.29) | 3.21 (3.17/3.24) | 3.17 (3.13/3.20) | Mean & 95% CI |
| | | Family members | 2.73 (2.69/2.76) | 2.67 (2.63/2.71) | 2.60 (2.57/2.64) | Mean & 95% CI |
| | | Friends | 2.37 (2.34/2.41) | 2.08 (2.04/2.11) | 2.15 (2.12/2.19) | Mean & 95% CI |
| | | Neighbours | 1.19 (1.16/1.23) | 1.17 (1.14/1.21) | 1.03 (0.99/1.06) | Mean & 95% CI |
| | | Colleagues | 1.99 (1.95/2.03) | 1.65 (1.61/1.70) | 1.66 (1.62/1.70) | Mean & 95% CI |
| | | Superiors | 1.49 (1.44/1.53) | 1.20 (1.15/1.24) | 1.20 (1.16/1.24) | Mean & 95% CI |
| | | Strangers/Other individuals | 1.04 (1.00/1.07) | 0.75 (0.71/0.78) | 0.75 (0.72/0.78) | Mean & 95% CI |
| | In your perception, how much time did you allocate towards helping others? | | 3.19 (3.13/3.25) | 3.13 (3.07/3.19) | 3.01 (2.95/3.07) | Mean & 95% CI |
| Stress | Perceived Stress Scale (PSS4) | | 5.86 (5.77/5.95) | 7.09 (6.99/7.19) | 6.05 (5.9/6.15) | Mean & 95% CI |
| Subjective expectation of competence | Allgemeine Selbstwirksamkeit Kurzskala (ASKU) | | 4.37 (4.34/4.39) | 4.04 (4.76/4.07) | 4.18 (4.16/4.21) | Mean & 95% CI |
| Internet | How much time did you spend online … | … in order to nurture social bonds? | 3.86 (3.79/3.93) | 4.84 (4.76/4.91) | 4.06 (3.99/4.14) | Mean & 95% CI |
| | | … in order to participate in debates / express your opinion? | 1.44 (1.38/1.50) | 1.79 (1.72/1.86) | 1.57 (1.51/1.64) | Mean & 95% CI |
| | | … for entertainment? | 4.50 (4.42/4.58) | 4.95 (4.88/5.03) | 4.32 (4.24/4.40) | Mean & 95% CI |
| | | … for distraction/ out of boredom? | 3.90 (3.82/3.98) | 4.21 (4.12/4.29) | 3.45 (3.37/3.53) | Mean & 95% CI |
| | | … for information research? | 5.66 (5.60/5.72) | 6.00 (5.94/6.06) | 5.37 (5.31/5.43) | Mean & 95% CI |
| | | … for work (at your external workplace)? | 4.07 (3.97/4.17) | 3.42 (3.31/3.53) | 3.29 (3.19/3.39) | Mean & 95% CI |
| | | … for work in home office? | 1.36 (1.28/1.44) | 3.44 (3.32/3.55) | 2.76 (2.66/2.87) | Mean & 95% CI |
| | | … to do home-schooling / education? | 1.18 (1.11/1.25) | 2.19 (2.10/2.28) | 1.74 (1.65/1.82) | Mean & 95% CI |
| | | … for other reasons? | 2.13 (2.06/2.21) | 2.38 (2.30/2.46) | 2.10 (2.03/2.17) | Mean & 95% CI |
| Addictive substances | How strong was your desire for the following activities? | (Online-)videogames | 1.10 (1.04/1.17) | 1.44 (1.37/1.52) | 1.17 (1.10/1.23) | Mean & 95% CI |
| | | Watch series, films or TV | 3.73 (3.66/3.81) | 4.59 (4.51/4.68) | 3.84 (3.76/3.92) | Mean & 95% CI |
| | | Online-shopping | 2.31 (2.24/2.38) | 2.85 (2.77/2.93) | 2.38 (2.31/2.45) | Mean & 95% CI |
| | | Use of social networks (e.g. Instagram, Facebook, TikTok, etc.) | 2.71 (2.62/2.80) | 3.19 (3.09/3.28) | 2.78 (2.69/2.87) | Mean & 95% CI |
| | | Online-gambling | 0.08 (0.06/0.09) | 0.09 (0.07/0.11) | 0.08 (0.06/0.10) | Mean & 95% CI |
| | | Internet pornography | 0.92 (0.87/0.98) | 0.98 (0.92/1.04) | 0.87 (0.81/0.93) | Mean & 95% CI |
| | | Other internet surfing | 2.68 (2.61/2.75) | 2.74 (2.66/2.82) | 2.21 (2.14/2.28) | Mean & 95% CI |
| | | … backpain? | 2.14 (2.07/2.21) | 2.37 (2.29/2.45) | 1.92 (1.84/1.99) | Mean & 95% CI |

| | | | | | | |
|---|---|---|---|---|---|---|
| Psychosomatic | How much did you suffer from … | … exhaustion / tiredness? | 3.22 (3.15/3.29) | 3.57 (3.49/3.66) | 2.97 (2.90/3.05) | Mean & 95% CI |
| | | … sleep disorders? | 2.28 (2.21/2.36) | 2.83 (2.74/2.91) | 2.21 (2.13/2.29) | Mean & 95% CI |
| | | … headache / migraine? | 1.43 (1.37/1.49) | 1.69 (1.62/1.76) | 1.34 (1.28/1.41) | Mean & 95% CI |
| | | … cardiovascular problems? | 0.62 (0.57/0.66) | 0.75 (0.70/0.80) | 0.62 (0.57/0.67) | Mean & 95% CI |
| | | … gastrointestinal complaints | 1.21 (1.15/1.27) | 1.36 (1.29/1.43) | 1.09 (1.03/1.15) | Mean & 95% CI |
| | | … cold symptoms (e.g. throat-, nose- und ear pain, cough, etc.)? | 1.45 (1.39/1.51) | 0.92 (0.87/0.98) | 0.46 (0.42/0.50) | Mean & 95% CI |
| Depression | Patient Health Questionnaire (PHQ4) | | 1.34 (1.30/1.38) | 1.89 (1.84/1.94) | 1.47 (1.43/1.52) | Mean & 95% CI |
| Chance | Please evaluate the following statements. | Crises or problems have also brought positive changes for me personally. | 4.40 (4.32/4.48) | 4.30 (4.23/4.38) | 4.35 (4.28/4.43) | Mean & 95% CI |
| | | Crises and problems have also brought positive changes for society in general. | 3.81 (3.74/3.88) | 3.87 (3.80/3.95) | 3.80 (3.72/3.87) | Mean & 95% CI |
| Coping | How much have the following things helped you to overcome crises and problems? | Support from others | 5.13 (5.05/5.20) | 4.98 (4.91/5.06) | 5.18 (5.11/5.26) | Mean & 95% CI |
| | | Physical exercise | 4.06 (3.97/4.15) | 3.96 (3.87/4.06) | 4.27 (4.18/4.36) | Mean & 95% CI |
| | | Spend time in nature | 4.96 (4.88/5.04) | 5.47 (5.39/5.55) | 5.79 (5.72/5.86) | Mean & 95% CI |
| | | Humour | 5.96 (5.90/6.02) | 5.85 (5.79/5.92) | 5.87 (5.81/5.94) | Mean & 95% CI |
| | | Religion, spirituality, meditation | 1.75 (1.67/1.83) | 1.66 (1.58/1.74) | 1.60 (1.52/1.68) | Mean & 95% CI |
| | | Cultural offerings (also online) | 3.33 (3.25/3.42) | 2.14 (2.06/2.22) | 2.55 (2.47/2.63) | Mean & 95% CI |
| | | Music | 5.14 (5.06/5.21) | 4.90 (4.82/4.98) | 4.90 (4.82/4.99) | Mean & 95% CI |
| | | Actively changing the situation | 5.04 (4.96/5.12) | 3.56 (3.48/3.65) | 4.34 (4.27/4.42) | Mean & 95% CI |
| | | Trying to accept the situation | 4.70 (4.63/4.77) | 5.27 (5.20/5.34) | 5.02 (4.95/5.09) | Mean & 95% CI |
| | | Distraction | 4.57 (4.50/4.64) | 4.67 (4.60/4.75) | 4.50 (4.43/4.58) | Mean & 95% CI |
| | | Open display of emotions when you are not doing well | 3.85 (3.77/3.93) | 3.67 (3.59/3.75) | 3.69 (3.61/3.77) | Mean & 95% CI |
| | | Mentally plan the next steps ahead | 5.35 (5.28/5.42) | 4.54 (4.46/4.61) | 4.78 (4.70/ 4.85) | Mean & 95% CI |
| Anxiety | How anxious were you? | | 1.25 (1.21/1.29) | 1.83 (1.78/1.89) | 1.42 (1.37/1.47) | Mean & 95% CI |
| Fear | How much did you fear … | … to run out of food? | 0.27 (0.24/0.30) | 1.30 (1.24/1.36) | 0.31 (0.28/0.34) | Mean & 95% CI |
| | | … to run out of toilet paper? | 0.21 (0.18/0.24) | 1.45 (1.38/1.52) | 0.22 (0.19/0.24) | Mean & 95% CI |
| | | … to run out of desinfectants, cleaning agents or soap? | 0.25 (0.22/0.28) | 1.36 (1.29/1.42) | 0.29 (0.26/0.32) | Mean & 95% CI |
| | | … to not have enough money at your disposal? | 1.01 (0.95/1.06) | 1.43 (1.36/1.50) | 1.02 (0.96/1.08) | Mean & 95% CI |
| | | … to catch a disease or a virus? | 1.44 (1.38/1.50) | 3.74 (3.66/3.82) | 2.71 (2.64/2.79) | Mean & 95% CI |
| | | … to lose your job? | 0.79 (0.74/0.85) | 1.51 (1.43/1.59) | 1.08 (1.01/1.15) | Mean & 95% CI |
| | | … that the health system could be overwhelmed? | 0.97 (0.91/1.04) | 4.27 (4.19/4.35) | 2.48 (2.41/2.55) | Mean & 95% CI |
| | | … that your existence is threatened? | 0.78 (0.73/0.84) | 1.67 (1.59/1.75) | 1.15 (1.09/1.22) | Mean & 95% CI |
| | | … that there could be an economic crisis? | 1.75 (1.68/1.81) | 4.30 (4.22/4.38) | 3.50 (3.42/3.58) | Mean & 95% CI |
| | | … that a political crisis could arise? | 2.22 (2.15/2.29) | 3.96 (3.88/4.04) | 3.59 (3.51/3.67) | Mean & 95% CI |
| | | … that international conflicts could intensify? | 3.37 (3.30/3.45) | 4.34 (4.26/4.42) | 3.96 (3.88/4.04) | Mean & 95% CI |

| | | | | | | |
|---|---|---|---|---|---|---|
| Fear behaviour | How much did you do the following things? | Stock up on food | 0.96 (0.91/1.01) | 2.40 (2.33/2.47) | 0.72 (0.68/0.77) | Mean & 95% CI |
| | | Stock up on toilet paper | 0.47 (0.44/0.51) | 1.51 (1.45/1.57) | 0.41 (0.38/0.44) | Mean & 95% CI |
| | | Stock up on desinfectants, cleaning agents or soap | 0.58 (0.54/0.62) | 1.55 (1.49/1.62) | 0.55 (0.51/0.59) | Mean & 95% CI |
| | | Withdraw large sums of cash from the account | 0.27 (0.24/0.30) | 0.58 (0.53/0.62) | 0.27 (0.24/0.30) | Mean & 95% CI |
| Resilience | How quickly did you recover after demanding / stressful moments? | | 5.06 (5.01/5.12) | 4.62 (4.55/4.69) | 4.95 (4.89/5.01) | Mean & 95% CI |
| | How difficult was it for you to recover from demanding / stressful situations? | | 5.00 (4.94/5.06) | 4.58 (4.52/4.65) | 4.89 (4.82/4.96) | Mean & 95% CI |

**Table B2. The table below provides an overview of the scales and self-generated questions administered to participants as part of state measures assessment.**

| Construct | Short | Questions | Items | Respondence scale | Reference |
|---|---|---|---|---|---|
| Life satisfaction | Lezu_state | How satisfied were you with your life? | | 0 (dissatisfied) – 8 (very satisfied) | Self-generated |
| Emotional state | Gefühlslage | My emotional state was characterized by.. | Valence | axis affect grid -4 (unpleasant feelings) – 4 (pleasant feelings) | Self-generated |
| | | | Arousal | axis affect grid -4 (low arousal/ activation) – 4 (high arousal/ activation) | |
| Place of residence | Wohnort | Did you live at your usual place of residence? | | Yes/No | Self-generated |
| Household | Haushalt_Personen | How many people did you live with? | | 0 (alone) – 5 (5 or more) | Self-generated |
| | HH_Personen | With whom of these people have you lived with? | Partner | Yes/No | Self-generated |
| | | | Parents | | |
| | | | Children | | |
| | | | Friends / Housemates | | |
| | Haushalt_pflegebedürftig | How many of those required care? | | 0 (None) – 5 (5 or more) | Self-generated |
| Safety precautions | Schutzmaßnahmen | Please evaluate to what extent you agree with the following statements: | I followed the regulations. | Slider:<br>0 (fully disagree) – 8 (fully agree) | Self-generated |
| | | | The regulations for the protection of the society were overall too strict. | | |
| | | | The regulations for the protection of the society were overall too weak. | | |
| | | | The people in my environment adhered too much to the regulations. | | |
| | | | The people in my environment did not adhere enough to the regulations. | | |
| Optimism | Optimismus_state | How optimistic were you? | | Slider:<br>0 (not at all) – 8 (highly optimistic) | Self-generated |
| Alcohol consumption | AUDIT | | | (Bush et al., 1998; Rumpf et al., 2001) | |
| | Alkohol_Kontrolle | Did you feel like your alkohol consumption has gotten out of control? | | Slider:<br>0 (not at all) – 8 (very much) | Self-generated |
| News | Nachrichten | How often did you consume news and information concerning current negative events? | | Slider:<br>0 (not at all) – 8 (very often) | Self-generated |
| Mobility | Mobilität | How often did you spend time outdoors in your time off? | | Slider:<br>0 (not at all) – 8 (very often) | Self-generated |
| Stress | Stress | How stressed were you? | | Slider:<br>0 (not at all) – 8 (highly stressed) | Self-generated |
| Engagement | Partizipation_sozial | How much did you participate in social / political engagement? | Social | Slider:<br>0 (not at all) – 8 (very much) | Self-generated |
| | Partizipation_politisch | | Political | | |
| Belonging | Belonging_Familie | Please draw circle so that they best represent your belonging to the following groups | Me and family | Draw circles (0-100) | Self-generated |
| | | | Me and friends | | |
| | | | Me and neighbours | | |
| | | | Me and Berlin population | | |
| | | | Me and German population | | |
| | | | Me and European population | | |
| | | | Me and world population | | |
| | | | Family | | |
| | | | Friends | | |
| | | | Neighbours | | |

| | | | | | |
|---|---|---|---|---|---|
| Trust | Vertrauen | How strongly did you trust.. | Fellow citizens | Slider: 0 (not at all) – 8 (very strongly) | Self-generated |
| | | | German public-legal media | | |
| | | | German police | | |
| | | | Berlin Senat | | |
| | | | German Chancellor | | |
| | | | German government | | |
| | | | German health system | | |
| | | | Science | | |
| Aggression | Aggression_Täter | How often have you been verbally or physically aggressive towards … | Partner | Slider: 0 (not at all) – 8 (very often) | Self-generated |
| | | | Family members | | |
| | | | Friends | | |
| | | | Neighbours | | |
| | | | Colleagues | | |
| | | | Superiors | | |
| | | | Strangers / other individuals | | |
| | Aggression_Opfer | How often have you been a victim of verbal or physical aggression by … | Partner | Slider: 0 (not at all) – 8 (very often) | Self-generated |
| | | | Family members | | |
| | | | Friends | | |
| | | | Neighbours | | |
| | | | Colleagues | | |
| | | | Superiors | | |
| | | | Strangers / other individuals | | |
| Children in household | Haushalt_Kinder | How many of the people in your household were children (younger than 18)? | | 0 (none) – 5 (5 or more) | Self-generated |
| | Alonerziehend | Were you a single parent? | | Yes/No | Self-generated |
| | Aggression_Kinder_screening | Has it happened in your household that a child has been a witness or victim of verbally or physically aggressive behaviour, or that it has been neglected or overwhelmed? | | Yes/No | Self-generated |
| Aggression children | Aggression_Kinder | How often has at least one adult living (/ who previously lived) in your household with your child? threatened to leave you or your child? (or has actually done it?) | | Slider: 0 (not at all) – 8 (very often) | Self-generated |
| | | How often has it occurred that a child in your household had to take on the responsibility of a significantly older child or adult? | | | |
| | | How often has an adult locked in a child of your household in a closet, attic, basement, garage, or other, possibly very narrow, dark place? | | | |
| | | How often did it occur that at least one parent (or other main caregiver / parent-like person) failed to understand the feelings of a child in your household and to be there for them? | | | |
| | | How often has at least one adult in your household purposely pushed, pinched, slapped, punched, or kicked a child in your household? | | | |
| | | How often has at least one adult in your household purposely pushed, pinched, slapped, punched, or kicked a child in your household? | | | |
| | | How often has a child in your household seen how adults living in your household arguing violently? | | | |
| | | How often has a child in your household seen adults beat their mother / father or another adult in the household so badly that they were severely injured? | | | |
| Income | Erwerb | Were you gainfully employed? | | Yes/No | Self-generated |

| | | | | | |
|---|---|---|---|---|---|
| Occupation | Beruf_Arbeitszeit | How many hours did you work on average per week | | Numeric entrance 0-100 | Self-generated |
| | Beruf_Aufkommen | How high was your workload? | | 0 (no workload) – 8 (very high) | Self-generated |
| | Beruf_Homeoffice | What percentage of your working time did you spend working in home office | | Slider: 0 (0%) – 100 (100%) | Self-generated |
| | Beruf_Arbeitsplatz | What percentage of your working time did you spend at your external workplace? | | Slider: 0 (0%) – 100 (100%) | Self-generated |
| Financial | Finanzen_Mittel | How would you rate your financial security? | | Slider: 0 (very low) – 8 (very high) | Self-generated |
| | Finanzen_Grundbedürfnisse | How difficult was it, to cover basic needs? (e.g. groceries, rent etc.)? | | Slider: 0 (not at all) – 8 (very difficult) | Self-generated |
| Contact | Kontakt_Person | How often did you have contact with… | Partner | Single choice:<br>0 (never) – 4 (very often) | Self-generated |
| | | | Family members | | |
| | | | Friends | | |
| | | | Colleagues | | |
| | | | Superiors | | |
| | | | Other individuals | | |
| | Kontakt_Person_Valenz | On average, how pleasant were the personal interactions with the following people? | Partner | Slider: -4 (very unpleasant) – 0 (neutral) – 4 (very pleasant) | Self-generated |
| | | | Family members | | |
| | | | Friends | | |
| | | | Colleagues | | |
| | | | Superiors | | |
| | | | Other individuals | | |
| Online contact | Kontakt_online | How often did you have online contact with … | Partner | Single choice:<br>0 (never) – 4 (very often) | Self-generated |
| | | | Family members | | |
| | | | Friends | | |
| | | | Colleagues | | |
| | | | Superiors | | |
| | | | Other individuals | | |
| | Kontakt_online_Valenz | On average, how pleasant were the personal interactions with the following people? | Partner | Slider: -4 (very unpleasant) – 0 (neutral) – 4 (very pleasant) | Self-generated |
| | | | Family members | | |
| | | | Friends | | |
| | | | Colleagues | | |
| | | | Superiors | | |
| | | | Other individuals | | |
| | | | Negative political events and media coverage | | |
| | | | Financial or economic problems | | |
| | | | Loss of job or insolvency of a private company for yourself or someone in your household | | |
| | | | Problems with the supply of basic needs or the procurement of goods and services | | |
| | | | Personal physical health problems | | |

| | | | | | |
|---|---|---|---|---|---|
| Burden | Belastung | How burdened did you feel by the following factors? | Physical health problems of friends, family members or colleagues | Slider: 0 (not at all) – 8 (highly burdened) | Self-generated |
| | | | Personal mental health problems | | |
| | | | Mental health problems of friends, family members or colleagues | | |
| | | | Personal increased risk of catching an infectious disease | | |
| | | | Increased risk of an infectious disease among friends, family members or colleagues | | |
| | | | Problems concerning childcare, difficulties in coordinating childcare and work | | |
| | | | Tension or conflicts at home or family conflicts | | |
| | | | Tension or conflicts with friends and / or acquaintances | | |
| | | | Stressful experiences (e.g. conflicts) at work, university or other occupation | | |
| | | | Increased workload | | |
| | | | Housing conditions such as tight living space (e.g. no access to a private outdoor area, too many roommates) | | |
| | | | Loss of a loved one through separation or death | | |
| | | | Limitations of social contacts and / or opportunities to participate in important social events | | |
| | | | Limited travel options (private or business) | | |
| | | | Limited leisure opportunities and / or little exercise | | |
| | | | Discrimination based on your migration status | | |
| | | | Discrimination based on your sexual orientation | | |
| | | | Discrimination based on your gender | | |
| | | | Other reasons | | |
| Prosocial | Prosozial_Frequenz_Other | How many times have the following people offered you help or have done something for you? | Partner | Single choice:<br>0 (never) – 4 (very often) | Self-generated |
| | | | Family members | | |
| | | | Friends | | |
| | | | Neighbours | | |
| | | | Colleagues | | |
| | | | Superiors | | |
| | | | Strangers/Other individuals | | |
| | Prosozial_Frequenz_Self | How often have you offered your help or have done something for the following people? | Partner | Single choice:<br>0 (never) – 4 (very often) | Self-generated |
| | | | Family members | | |
| | | | Friends | | |
| | | | Neighbours | | |
| | | | Colleagues | | |

| | | | | | |
|---|---|---|---|---|---|
| | | | Superiors | | |
| | | | Strangers/Other individuals | | |
| | Prosozial_Aufwand | In your perception, how much time did you allocate towards helping others? | | Slider: 0 (very little) – 8 (a lot) | Self-generated |
| Stress | Perceived Stress Scale (PSS4) | | | (PSS-4; Cohen, Kamarck & Mermelstein, 1983) | |
| Subjective expectation of competence | Allgemeine Selbstwirksamkeit Kurzskala (ASKU) | | | (ASKU; Beierlein et al., 2013) | |
| Internet | Compulsive Internet use scale (CIUS) | | | (Bush et al., 1998; Rumpf et al., 2001) | |
| | Internet | How much time did you spend online … | … in order to nurture social bonds? | Slider: 0 (none at all) – 8 (a lot) | Self-generated |
| | | | … in order to participate in debates / express your opinion? | | |
| | | | … for entertainment? | | |
| | | | … for distraction/ out of boredom? | | |
| | | | … for information research? | | |
| | | | … for work (at your external workplace)? | | |
| | | | … for work in home office? | | |
| | | | … to do home-schooling / education? | | |
| | | | … for other? | | |
| Addictive substances | Suchtmittel | How strong was your desire for the following activities? | (Online-)videogames | Slider: 0 (not at all) – 8 (very strong) | Self-generated |
| | | | Watch series, films or TV | | |
| | | | Online-shopping | | |
| | | | Use of social networks (e.g. Instagram, Facebook, TikTok, etc.) | | |
| | | | Online-gambling | | |
| | | | Internet pornography | | |
| | | | Other internet surfing | | |
| Psychosomatic | Psychosomatik | How much did you suffer from … | … backpain? | Slider: 0 (not at all) – 8 (very much) | Self-generated |
| | | | … exhaustion / tiredness? | | |
| | | | … sleep disorders? | | |
| | | | … headaches / migraine? | | |
| | | | … cardiovascular problems? | | |
| | | | … gastrointestinal complaints | | |
| | | | … cold symptoms (e.g. throat-, nose- und ear pain, cough, etc.)? | | |
| Depression | Patient Health Questionnaire (PHQ2) | | | (PHQ-2; Löwe, Kroenke & Gräfe, 2005) | |
| Loneliness | Einsamkeit | How lonely did you feel? | | Slider: 0 (not at all) – 8 (very lonely) | Self-generated |
| Chance | Chance | Please evaluate the following statements. | Crises or problems have also brought positive changes for me personally. | | Self-generated |

| | | | | | |
|---|---|---|---|---|---|
| | | | Crises and problems have also brought positive changes for society in general. | Slider: 0 (not true at all) – 8 (perfectly true) | |
| Coping | Coping | How much have the following things helped you to overcome crises and problems? | Support from others | Slider: 0 (not at all) – 8 (very much) | Self-generated |
| | | | Physical exercise | | |
| | | | Spend time in nature | | |
| | | | Humour | | |
| | | | Religion, spirituality, meditation | | |
| | | | Cultural offerings (also online) | | |
| | | | Music | | |
| | | | Actively changing the situation | | |
| | | | Trying to accept the situation | | |
| | | | Distraction | | |
| | | | Open display of emotions when you are not doing well | | |
| | | | Mentally plan the next steps ahead | | |
| Anxiety | Ängstlich | How anxious were you? | | Slider: 0 (not at all) – 8 (very anxious) | Self-generated |
| | Generalized Anxiety Disorder Scale (GAD-2) | | | (GAD-2; Kroenke et al., 2007) | |
| Fear | Angst | How much did you fear … | … to run out of food? | Slider: 0 (not at all) – 8 (very anxious) | Self-generated |
| | | | … to run out of toilet paper? | | |
| | | | … to run out of disinfectants, cleaning agents or soap? | | |
| | | | … to not have any money at your disposal? | | |
| | | | … to get infected with diseases or viruses? | | |
| | | | … to lose your job? | | |
| | | | … that the health system could be overloaded? | | |
| | | | … that your existence is threatened? | | |
| | | | … that there could be an economic crisis? | | |
| | | | … that a political crisis could arise? | | |
| | | | … that international conflicts could intensify? | | |
| Fear behaviour | Angst_Verhaltensweisen | How much did you do the following things? | Stock up on food | Slider: 0 (not at all) – 8 (very much) | Self-generated |
| | | | Stock up on toilet paper | | |
| | | | Stock up on disinfectants, cleaning agents or soap | | |
| | | | Withdraw large sums of cash from the account | | |
| Resilience | Widerstand_schnell | How quickly did you recover after demanding / stressful moments? | | Slider: 0 (very slowly) – 8 (very quickly) | Self-generated |
| | Widerstand_leicht | How difficult was it for you to recover from demanding / stressful situations? | | | |

**Perspective Taking:**

These perspective taking exercises were performed prior to participants completing the retrospective questionnaires for T1, T2, T3.

**1. T1 – Pre-lockdown period.**

The first phase covers the **period before the acute corona crisis** and relates to the month of **January 2020**

**[January 2020 (before to the crisis)]**

**The following questionnaire relates to the phase in January 2020, and thus to the time frame prior to the acute corona crisis.**

Please take a moment to remember this period. You recently celebrated Christmas and New Year's Eve and you have just started into the year 2020. Newspapers reported about the bushfires in Australia, the climate demonstrations related to the World Economic Forum in Davos and the panda bear twins in the Berlin Zoo. Please think specifically about personal events in January 2020, as well as any regular activities or trips you did during that period. Also, please review the expectations and plans you had at the start of the year 2020. Ideally, you could take a look into your personal planner to recall personal appointments or meetings to put yourself into that time period as good as possible.

**2. T2 – Lockdown period.**

The second phase is defined as the **period of lockdown** and refers to the month **between mid-March and mid-April 2020**

**[mid-March – mid-April 2020 (lockdown)]**

**The following questionnaire refers to the lockdown phase between mid-March and mid-April 2020.**

Please take a moment to remember this period. After carnival meetings in North Rhine-Westphalia, the coronavirus also spread faster and faster in Germany. News from Italy and Spain showed long ques in front of supermarkets and overcrowded hospitals. In Germany, schools, stores, restaurants, etc. were closed. Public life mostly came to a stop. Angela Merkel addressed the population in a TV speech and called on people to stay at home whenever possible. Please think specifically about personal events in mid-March and mid-April 2020, as well as any regular activities or trips you did during that period. Ideally, you could take a look into your personal planner to recall personal appointments or meetings to put yourself into said time period as best as possible.

**3. T3 – Re-opening period.**

The third phase describes the **timeframe of relaxation of the restrictions** and refers to the month of **June 2020**

**[June 2020 (relaxations)]**

**The following questionnaire refers to the phase of relaxations in June 2020.**

Please take a moment to remember this period. Stores, hairdressers as well as gastronomy gradually reopened. Public life resumed with restrictions, such as the requirement to wear a mask. Anti-racism protests following the death of George Floyd were held in Germany and elsewhere. COVID-19 broke out on a large scale at the Tönnies slaughterhouse in Westphalia, and the Corona warning app was released. Berlin schools started their summer vacations and some headed off to holidays. Now please also think about personal events in June 2020 as well as regular activities or trips you pursued during this period. It is best to look at your calendar again to recall private appointments or encounters and thereby put yourself in this time as well as possible.
