# Supplementary material for: Coping with the COVID-19 Pandemic: Perceived Changes in Psychological Vulnerability, Resilience and Social Cohesion before, during and after Lockdown
Source: Int J Environ Res Public Health. 2022 Mar 10;19(6):3290. doi: 10.3390/ijerph19063290 (PMC8952288; doi:10.3390/ijerph19063290)
Supplement: Supplementary file 1 [file ijerph-19-03290-s001.zip › Supplement S1.pdf]

The CovSocial Project Phase 1:

Online study about social cohesion and mental health during the SARS-CoV-2 pandemic

Design, Sample, and Measurements

The CovSocial Team

Social Neuroscience Lab
Max Planck Society
Campus Nord, Haus 5
Humboldt-Universität zu Berlin
Phillipstraße 13
10099 Berlin
Germany
Phone: +49 302 09346 180
Fax: +49 302 09346 189
E-Mail: singer@social.mpg.de

Table of Contents

1 Introduction ____ 3

2 Design and Timeline ____ 7

3 Recruitment, Dropouts, and Outlier Detection ____ 10

4 Final Sample Description and Representativeness of the Sample ____ 13

5 Measures ____ 31

6 Results ____ 35

1 Introduction

The CovSocial project aims to unravel the impact of the SARS-CoV-2 pandemic and the associated lockdown on various aspects of mental health and social cohesion of the Berlin population. The pandemic has had far-reaching consequences on the daily lives of the people owing to economic ramifications and social restrictions brought about by the spread of the COVID-19 disease and the lockdowns imposed to curb its spread. Recent rapid reviews of empirical studies on mental health during the pandemic have shown a drastic effect on mental well-being and the feelings of belonging and connectedness of the society. Therefore, in the current project we aimed to understand the impact of the pandemic on the key psychological constructs of vulnerability, resilience, and social cohesion in a sample of Berliners. Vulnerability, in the current context, constitutes the predispositional and genetic factors that make an individual more likely to develop psychological disorders such as depression, anxiety and dysfunctional levels of stress. These vulnerability factors comprise the risk factors that make an individual vulnerable to mental health problems. On the flip side, resilience and social cohesion, in our present study, constitute protective factors that protect and individual from the development of psychological disorders. Resilience is considered to be the ability to bounce back from adversities, such as stressors, and comprises of adaptive strategies and coping mechanisms. Similarly, social cohesion is seen as a societal-level protective mechanism that consists of multilevel and multidimensional core mechanisms that unite individuals in communities and societies through social engagement, feelings of belonging and trust, and social interaction. Given the severe impact of the pandemic on the individual and social functioning, in the current project we aimed to examine which of these risk (vulnerability) and protective (resilience and social cohesion) factors might be key to

understanding mental health and social relations in the present pandemic. Therefore, in this first phase of the CovSocial project, we assessed different indicators of vulnerability, resilience, and social cohesion, including genetic markers of these indicators, and the changes in these indicators over the period of January 2020 to April 2021. The findings from this first phase of the project will help in identifying individuals who might be more at risk due to the global stressors, and what protective factors can diminish the impact of such stressors and protect mental health. This will also allow for the development and testing of psychological interventions aimed at improving resilience and social cohesion while lowering vulnerability during similar stressors of global scale, which will further improve our readiness for future collective crises of such nature. Consequently, in a further second phase of the project, we will examine the impact of online socioemotional and mindfulness-based interventions on vulnerability, resilience and social cohesion during the pandemic (see Figure S1 for an overview of the entire project).

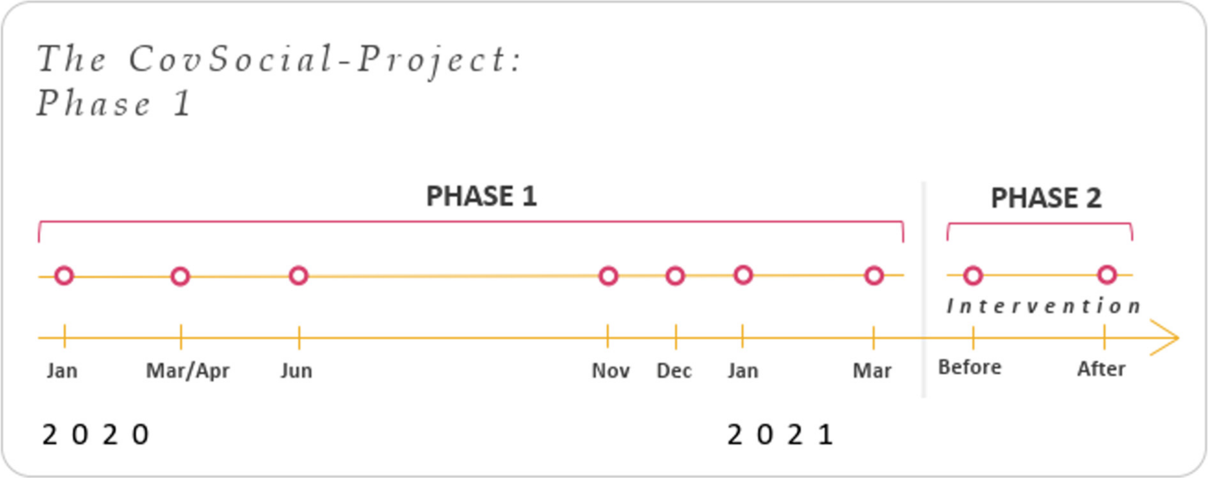

Figure S1. Design of the CovSocial Project. Phase 1 consists of a retrospective and longitudinal examination of indicators of vulnerability, resilience and social cohesion during the SARS-CoV-2 pandemic. Phase 2 of the project consists of evaluating the impact of socioemotional and mindfulness-based intervention on these indicators.

This document describes the design, sample, methods, and measures including key descriptive statistics of the first three retrospective measurement timepoints of the first phase of the CovSocial project (www.covsocial.de). The first phase of the project involved a longitudinal study on the effects of the SARS-CoV-2 pandemic and the related lockdown on measures of mental vulnerability, psychological resilience, and social cohesion in a Berlin sample. The purpose of this document is to provide a detailed description of the design and methods with full transparency, and seeks to serve as an important supplementary material for all peer-reviewed publications resulting from the first assessment phase (with measurement timepoints T1-T3) of this project.

The CovSocial project is headed by Prof. Dr. Tania Singer, scientific head of the Social Neuroscience Lab of the Max Planck Society in Berlin, and is conducted by both Tania Singer's team at the social neuroscience lab and a large team of cooperation partners from different Berlin universities and from the Max Planck Institute for Psychiatry, Munich. Following cooperation partners were part of phase 1: Prof. Dr. Mazda Adli (Chief Doctor, Fliedner Clinic Berlin, and Head of Research Department on Affective Disorders, Department of Psychiatry and Psychotherapy, Charité – Universitätsmedizin Berlin), Prof. Dr. Elisabeth Binder (Executive Director, Department for Translational Research in Psychiatry, Max Planck Institute of Psychiatry, Munich), Prof. Dr. Sonja Entringer (Institute for Medical Psychology, Charité – Universitätsmedizin Berlin), Prof. Dr. Christine Heim (Director, Institute for Medical Psychology, Charité – Universitätsmedizin Berlin), and Prof. Dr. Manuel Voelkle (Professor, Psychological Research Methods at the Institute for Psychology, Humboldt Universität zu Berlin).

More than 50,000 individuals from the population of Berlin residents were invited to take part in the first phase of the CovSocial study which was mainly consisting of an online assessment via questionnaires. In the present document, we report additional information on the recruitment process, and important descriptive statistics regarding the sample, for example, dropouts, outliers, and composition of the sample. Specific details related to hypotheses, methods, and results will be detailed in the main peer-reviewed publications emerging from the project.

2 Design and Timeline

The phase 1 of the CovSocial project employed a cross-sectional, retrospective longitudinal design. The study consisted of a total of 7 assessment timepoints: January 2020 (T1), March-April 2020 (T2), June 2020 (T3), November 2020 (T4), December 2020 (T5), January 2021 (T6), and March-April 2021 (T7). Please see Figure S2 for an overview of phase 1. Timepoints T1-T3 were assessed in a retrospective manner during the period of September 11 to December 7 2020, while T4-T7 were completed longitudinally with T4 being completed in December 2020, T5 in January 2021, T6 in February 2021, and T7 in April 2021. In the present supplementary materials, we only refer to the assessments for timepoints T1-T3 (see Figure S3). The longitudinal assessment T4-T7 will be covered in subsequent publications as the phase 1 of the project is ongoing and the results from the later timepoints are yet to come in.

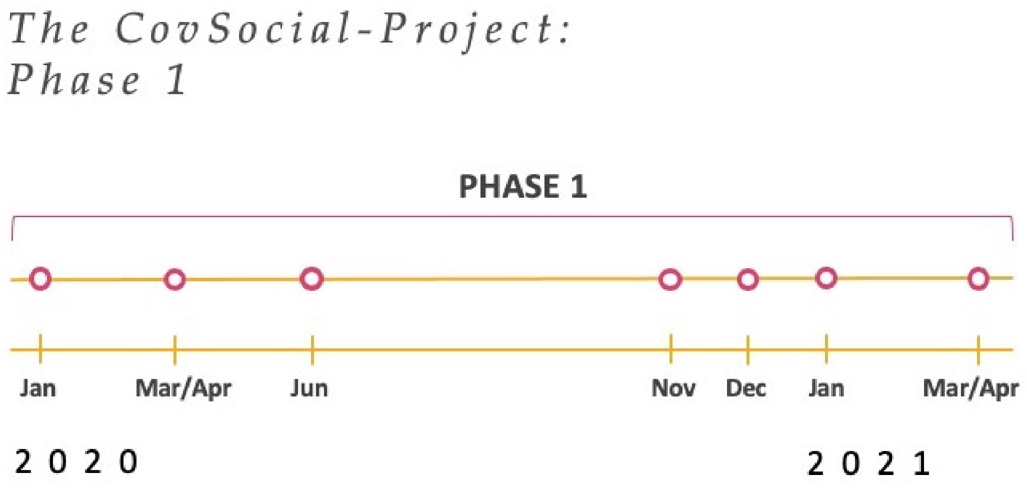

Figure S2. A depiction of the timeline of the entire phase 1 of the CovSocial project.

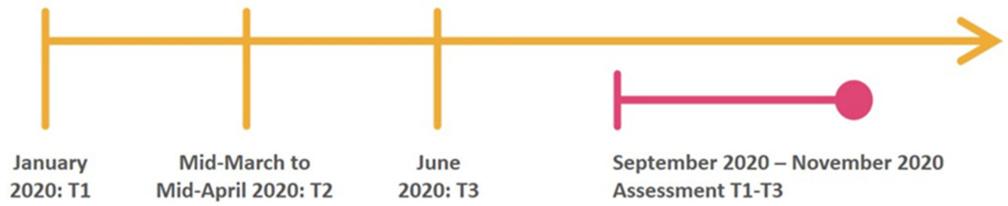

Figure S3. A depiction of the timeline of retrospective timepoints of interest, and their actual assessment in the phase 1 of the CovSocial project.

Participants completed the following types of questionnaires: a) questions pertaining to demographic information and context variables, b) questions assessing trait-level indicators, and c) questions assessing state-level indicators for the seven different time points. Questions related to demographic information and context variables were assessed only once, measured in one single block. Similarly, questions pertaining to *trait-level* factors indicators were measured only once, but were spread out over three blocks. However, participants completed the same questions assessing *state-level* indicators 7 times. First, during the retrospective assessment of T1-T3, participants completed 3 blocks of same questions assessing state-level indicators, one each for the three timepoints. Then, longitudinally, participants completed one block each of questions assessing state-level indicators for T4-T7. In the present supplementary materials, we will only refer to the state-level indicator assessment for T1-T3. Participants completed the three blocks of state-level indicator assessment for T1-T3 in a retrospective manner, with each block completed from the perspective of a specific timepoint: January 2020 (Pre-lockdown period; T1), Mid-March to Mid-April 2020 (Lockdown period; T2), and June 2020 (Post-Lockdown period; T3). The blocks assessing state-level factors required participants to take the perspective of the particular timepoints, for example January, and respond to that particular block of questions

based on how they felt and behaved during January. Please see *Chapter 7* for detailed information related to the procedure of retrospective assessment. For assessment during September to November 2020, participants were presented all the three types of questionnaires in the form of blocks in the following order: 1) Demographic questions, 2) T1 state-level indicator questions (Pre-lockdown period), 3) Trait 1 (first block of trait-level questionnaires), 4) T2 state-level indicator questions (Lockdown period), 5) Trait 2 (second block of trait-level questionnaires), 6) T3 state-level indicator questions (Post-lockdown period), and 7) Trait 3 (third block of trait-level questionnaires).

The participants completed the blocks of questionnaires during the period 11 September to 7 December 2020. Participants could complete all the blocks of questionnaires in one sitting or could re-login multiple times to complete the blocks at their convenience.

3 Recruitment, Dropouts, and Outlier Detection

Participants were recruited during the period August 2020 to November 2020. Participants registered for the study through the CovSocial project webpage (www.covsocial.de) by making a personal account on the website (please see Figure S4 for the dedicated landing page of the website/webapp in German and in English).

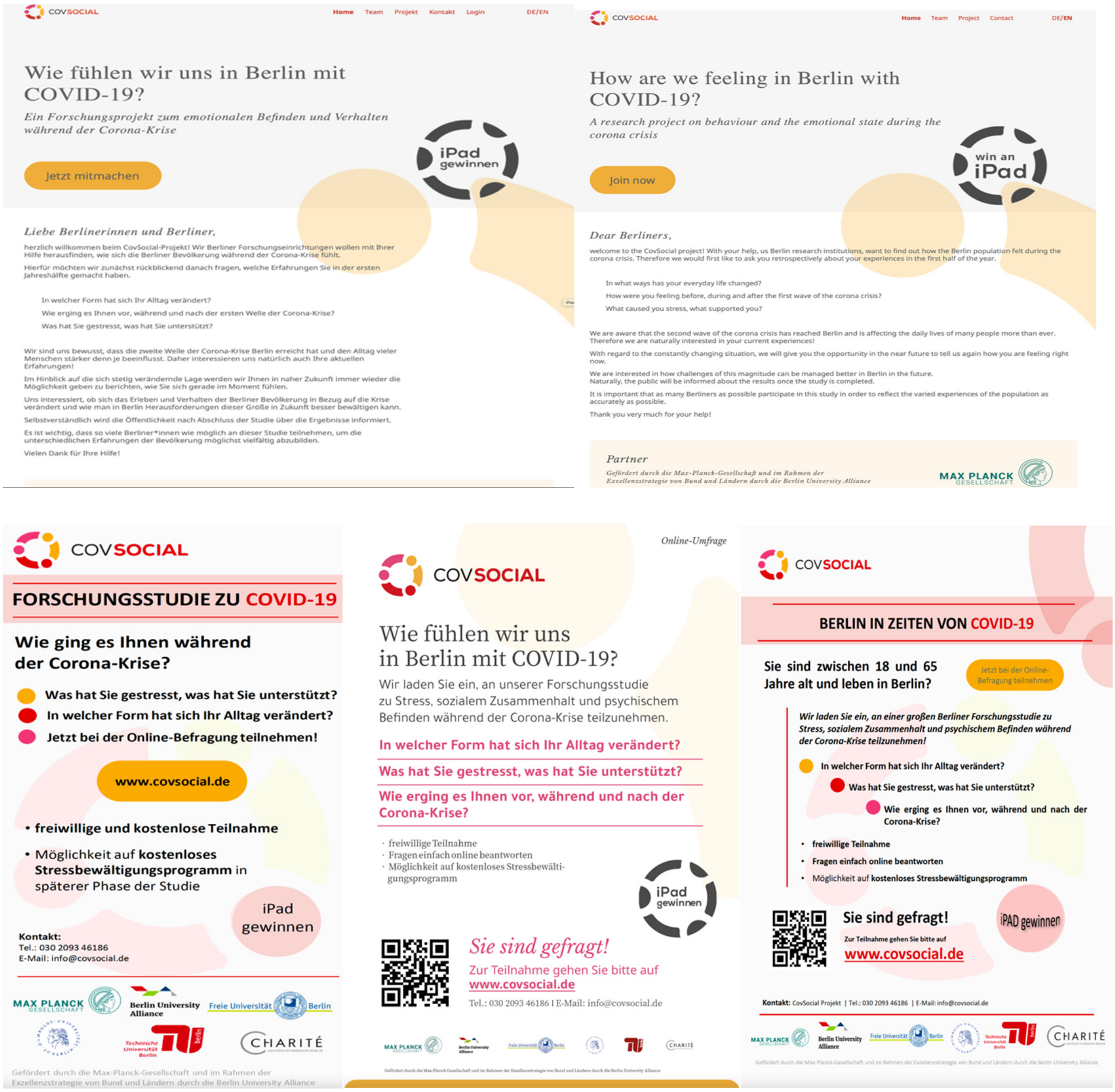

Figure S4. The landing page of the CovSocial website in German and English (Top panels). Three different versions of the recruitment poster used in newspapers, posted on social media, and used as flyers that were put up in subway stations and public transport hubs (Bottom panels).

3.1 Recruitment procedure

Participants were recruited from the population of the city of Berlin, Germany. For phase 1 of the CovSocial project, we aimed to recruit a total of 2000 participants between ages of 18 and 65 years old.

Figure S4 provides the original German version of the recruitment text that was used in the letters, flyers, posts, and advertisements. Figure S5 provides an overview of how many participants were recruited through the various avenues of recruitment.

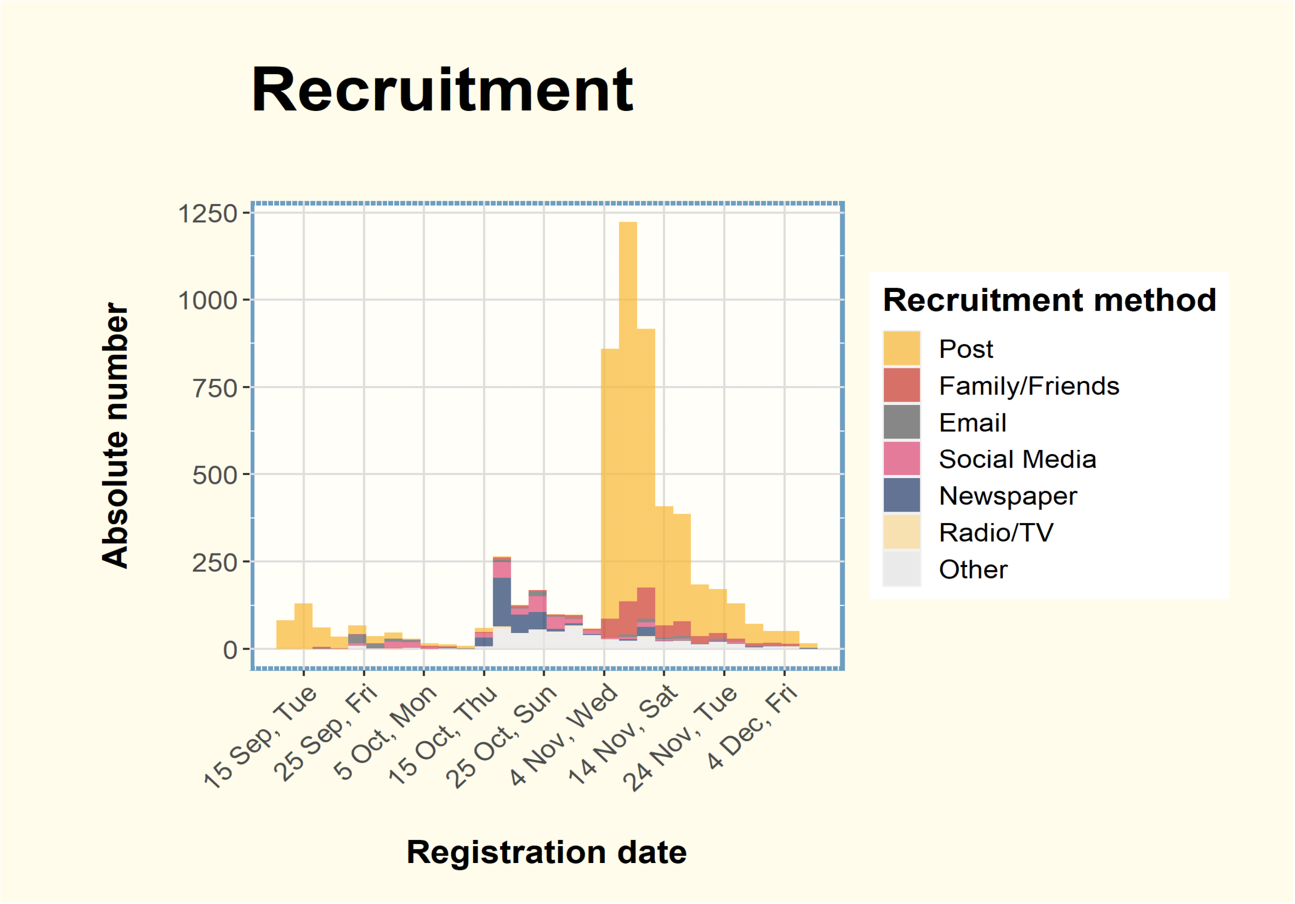

Figure S5. An overview of how many participants were recruited through the various avenues of recruitment over time.

3.2 Initial sample and dropouts

Please see Figure S5 below for a depiction of dropouts from registration to the completion of the study. The dropped-out participants either only registered to take part in the study or only provided responses to less than 7 blocks of questions, i.e., they did not complete all the questionnaires.

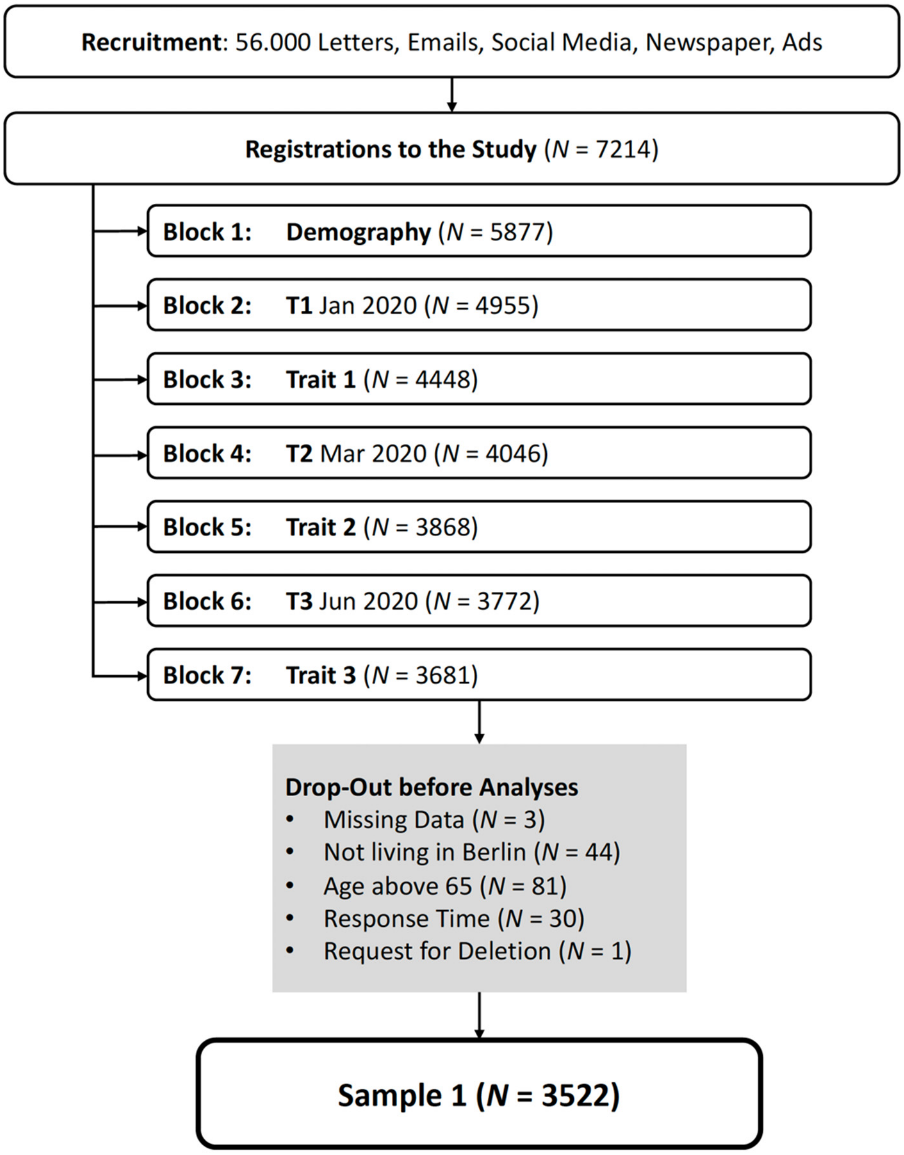

Figure S6. A depiction of the recruited sample, dropouts at every stage of the study, and the process of reaching the final sample. Sample 1 indicates the final sample of participants that completed the T1-T3 assessment. In subsequent publications we will give an overview of the dropout of participants for T4-T7 assessments and genetic markers assessment.

4 Final Sample Description and Representativeness of the Sample

4.1 Final sample description

Upon the dropouts and the removal of outliers, a final sample of 3522 individuals was obtained. Now, using the data collected from the first block of questions pertaining to demographics and context variables, we describe our sample. Our final sample had an average age of 43.95 years, with 37.39% individuals in 51-65 years age group and 8.63% individuals in 18-25 years age group (see Figure S7). In our sample, 65.11% participants were females and 34.89% were males (see Figure S8 for an overview of sex according to age, and for an overview of gender distribution). Furthermore, 61.04% individuals in our sample reported having a heterosexual identity, while 10.02% individuals reported having a homosexual identity, and 1.7% reported having an asexual identity (see Figure S9). Further, 10.9% individuals in our sample reported having a migratory background, while others did not report a migratory background (see Figure S10).

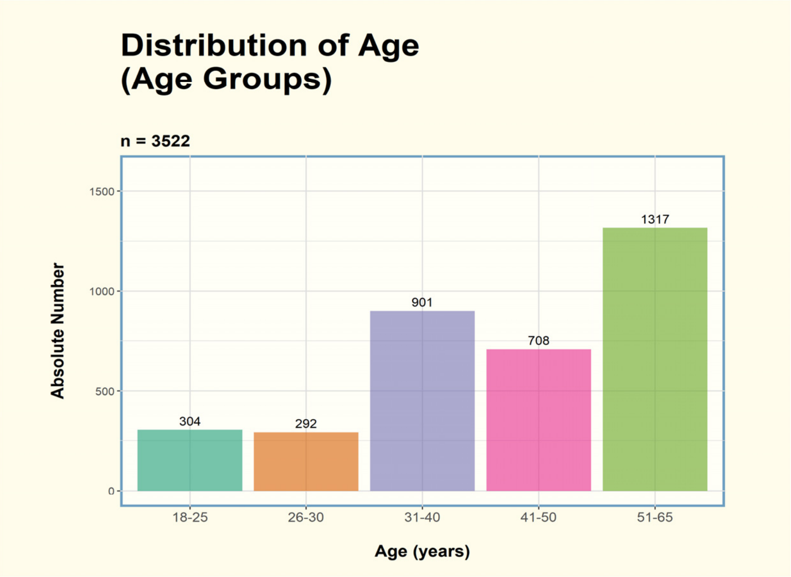

Figure S7. An overview of the number of individuals in different age groups in our sample.

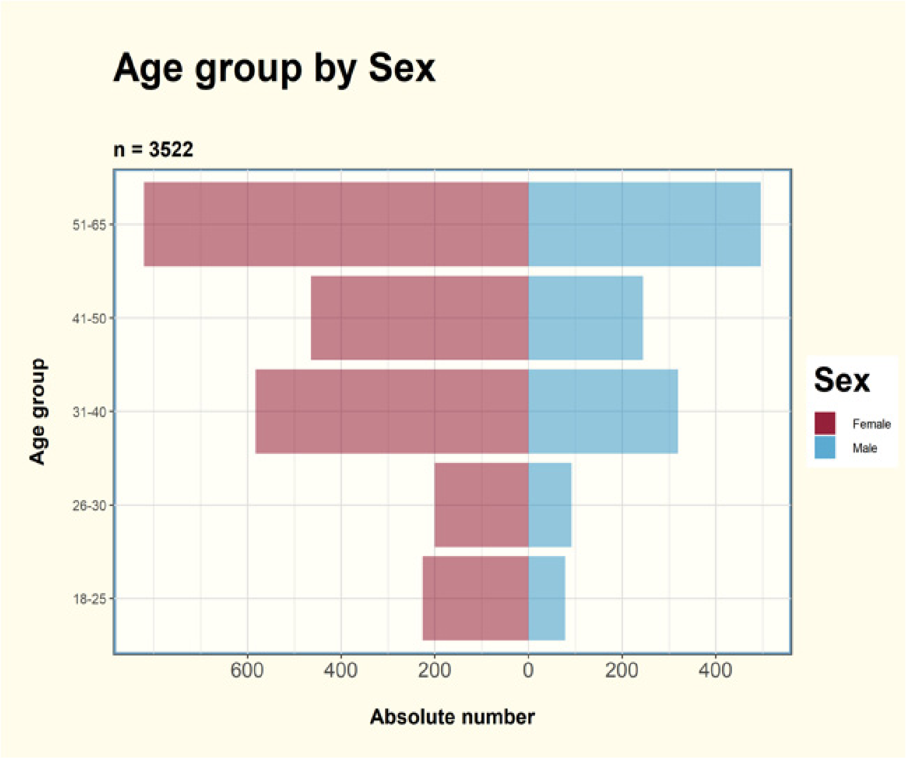

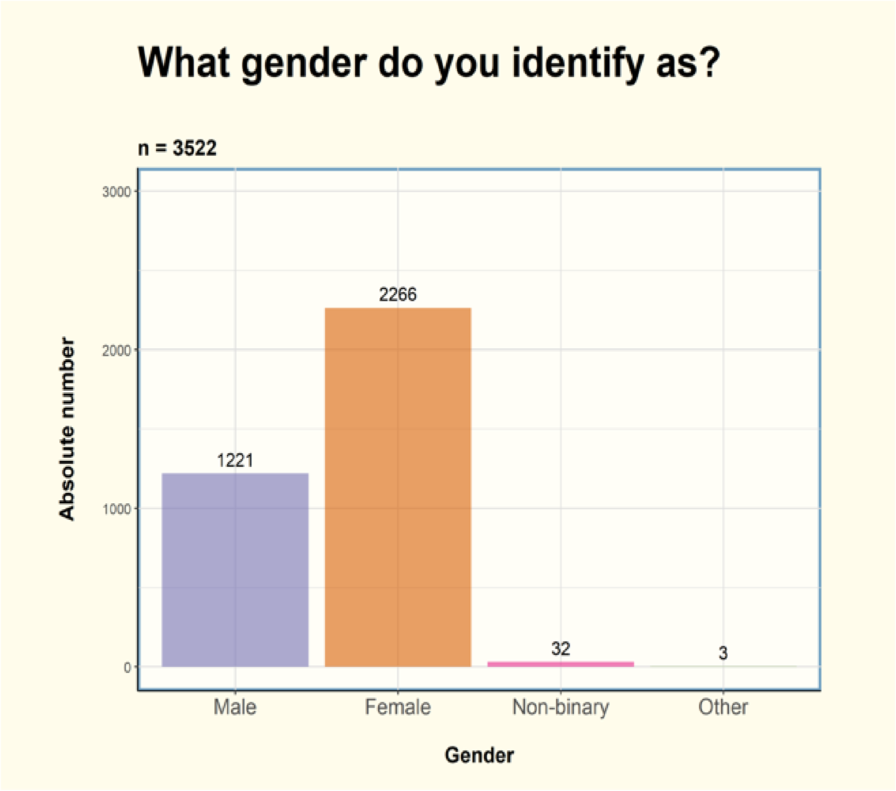

Figure S8. Top panel shows the distribution of number of males and females according to age groups in our sample. The bottom panel shows the distribution of gender identity of the participants.

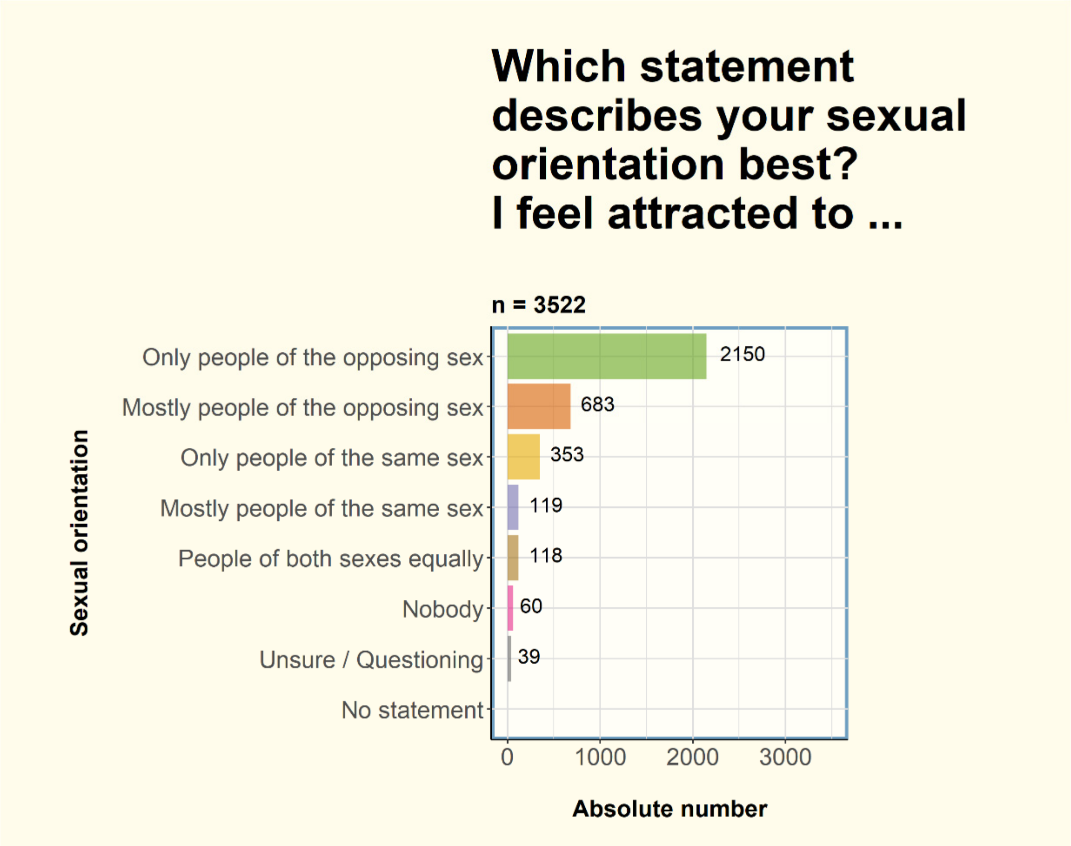

Figure S9. An overview of the sexual orientation of participants in our sample.

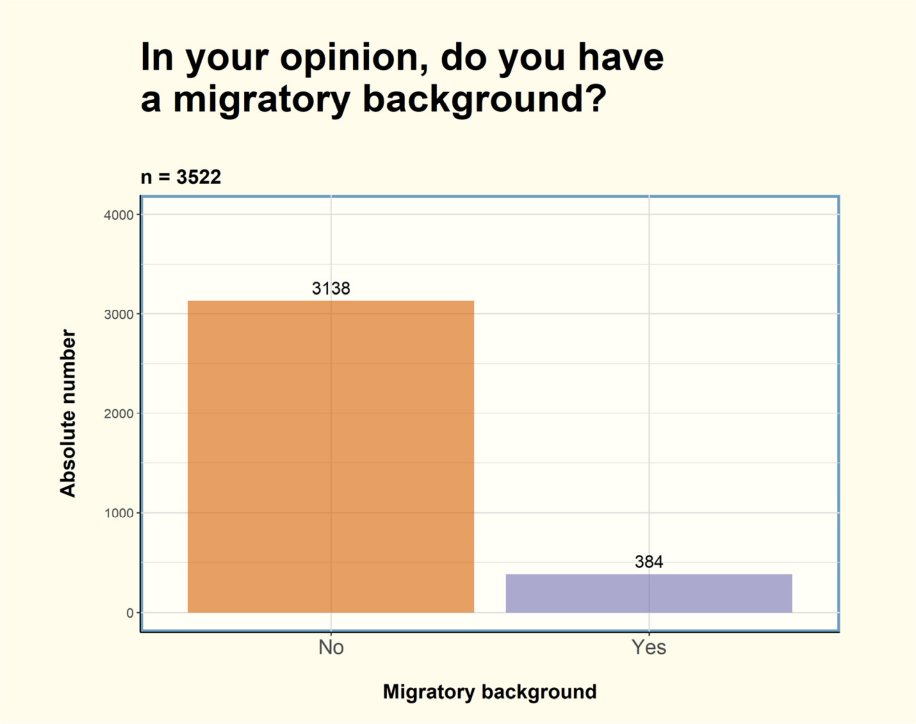

Figure S10. An overview of migration background in our sample.

Most individuals in our sample (74.39%) had completed higher education entrance qualification (see Figure S11 for an overview of education attainment levels, and the number of years of education), and 64.56% participants had obtained a Bachelor degree or diploma or higher. With respect to employment, 55% individuals in our sample were engaged in full-time employment, while 13.74% reported having no employment (see Figure S12). With respect to socioeconomic status, €3000 - 3250 was the median range of income in our sample. Moreover, 48.52% individuals in our sample reported they were unmarried, and 36.97% individuals reported being married and living with spouse (see Figure S13). Furthermore, 72.43% of the individuals in our sample reported no history of psychiatric diagnosis, while 24.87% reported history of psychiatric illness with depressive disorders being the most common category of diagnosis (see Figure S14). A description of the final sample, in terms of demographic and context variables, is provided in Table S13.

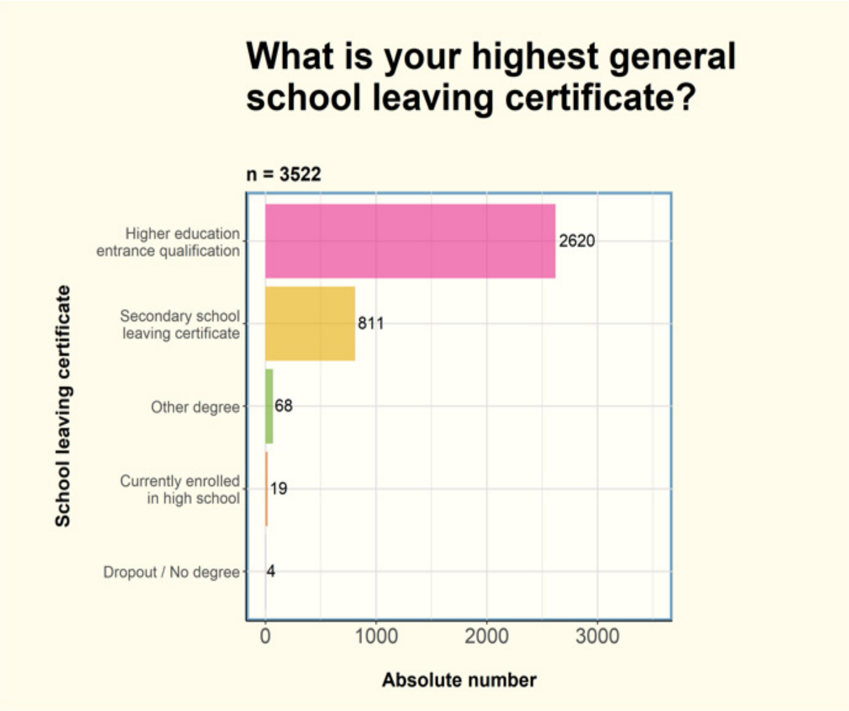

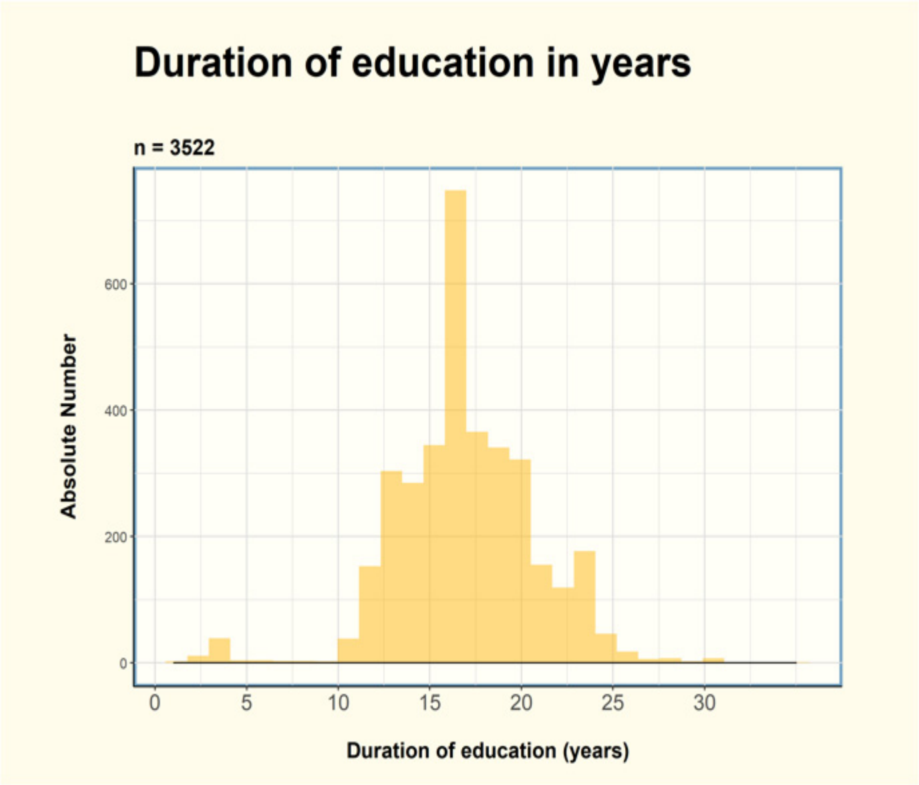

Figure S11. Top panel gives an overview of the level of high school education obtained in our sample. Bottom panel gives an overview of the distribution of number of education years in our sample.

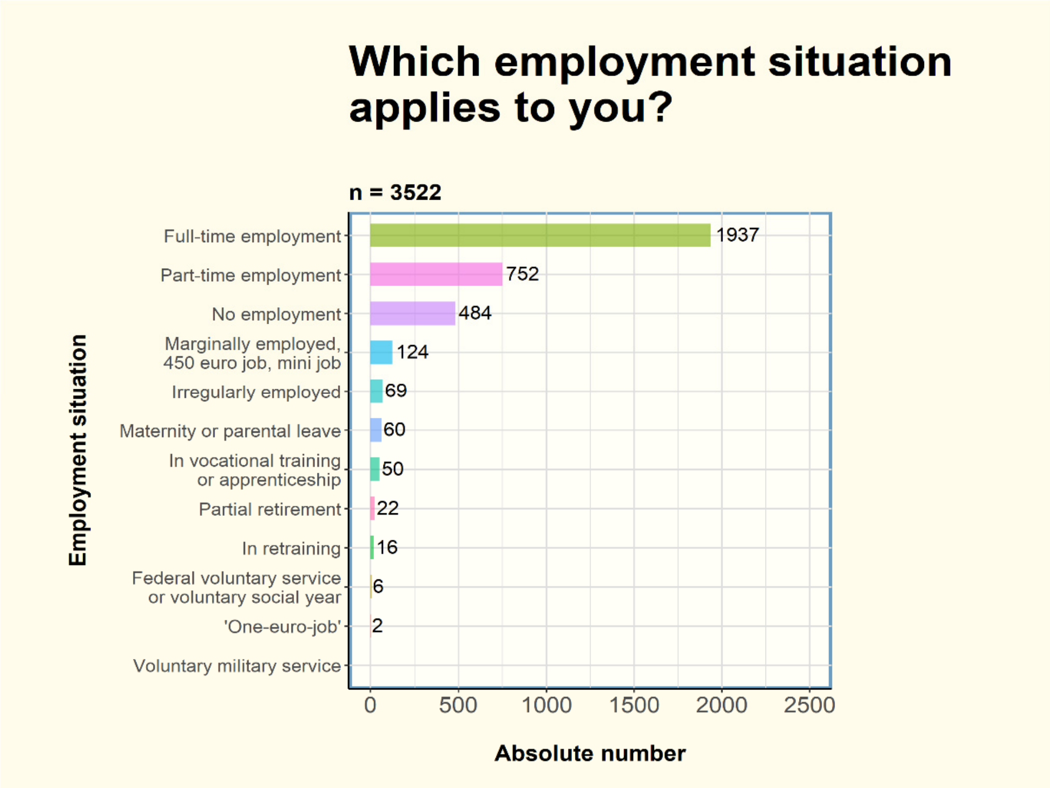

Figure S12. An overview of the employment situation of the participants in our sample.

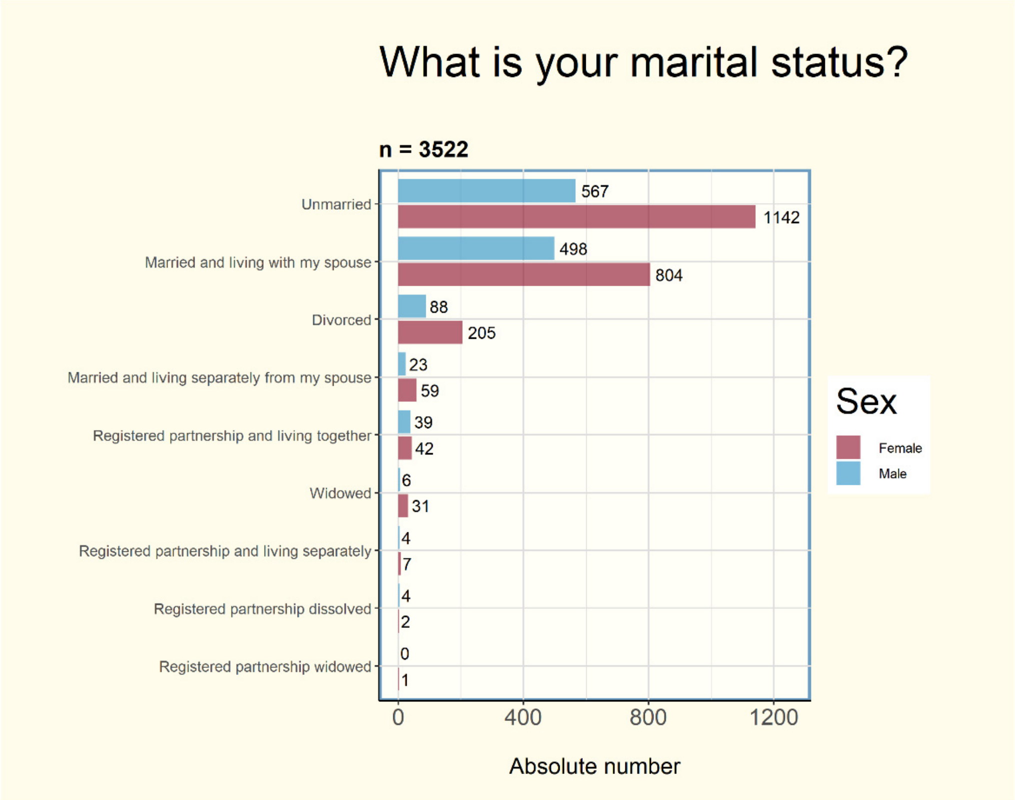

Figure S13. An overview of the marital status of the participants in our study.

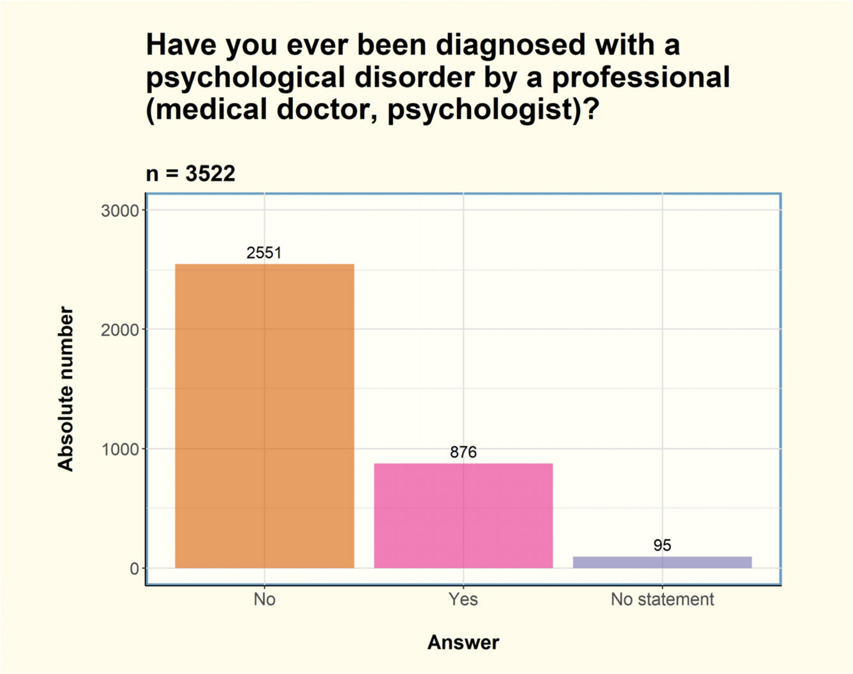

Figure S14. Left panel provides information on how many people reported having or not having a past or present psychiatric diagnosis. Right panel provides an overview of the category of the reported psychiatric diagnosis in the individuals who reported having a diagnosis.

Table S13<br>Basic Demographics	Total sample<br>*N* = 3522
Grouping	
Age	Mean age = 43.95<br>(*SD* = 12.69)
Gender	
Male	1221
Female	2266
Non-binary	32
Other	3
Sex	
Male	1229
Female	2293
Marital status	
Unmarried	1709
Married living with spouse	1302
Married living separately from spouse	82
Divorced	293
Widowed	37
Registered Partnership living together	81
Registered Partnership living separately	11
Registered Partnership dissolved	6
Registered Partnership widowed	1
German citizenship	

Yes	3316
Other	206
Place of birth	
Germany	3177
Other	345
Father’s place of birth	
Germany	2955
Other	567
Mother’s place of birth	
Germany	3002
Other	520
Migratory Background	
Yes	384
No	3138
Native Language	
German	3347
Other	175
Highest general school leaving certificate	
Full-time student	19
High School drop-out	4
Secondary school leaving certificate	73
Polytechnic High School (GDR, 8th / 9th grade)	5
Secondary school leaving certificate	447
Polytechnic High School of the (GDR, 10th grade)	286
Entrance qualification for a university of applied science	285
General or subject-linked higher education entrance qualification	2335
Other degree	68
Vocational training/ university degrees	
In vocational training	303
High School student and attending vocational advanced school	17
No professional qualification	130
Completed vocational training (apprenticeship)	909
Professional qualification from vocational training	0
Preparatory service for service in public administration	28
Completion of a year-long training at a health school	14
Completion of a two to three-year training course at a health school	203
Completion of training as an educator	111
Graduated from a technical college in the GDR	119
Completion of a master craftsman, technical school, etc.	205
Bachelor	502
Diploma	783
Master	794
PhD	195
Other professional qualification	210
Employment situation	
Full-time employment	1937
Part-time employment	752
Partial retirement	22

Marginally employed, 450 Euro job, mini job	124
“One-euro-job”	2
Occasionally or irregularly employed	69
In vocational training / apprenticeship	50
In retraining	16
Voluntary military service	0
Federal voluntary service or voluntary social year	6
Maternity, parental leave, or other leave of absence	60
No employment	484
Type of employment: Self-employed/ freelancing	
Yes	520
No	2395
Amount of people contributing to the income of the household	
1	1562
2	1852
3	81
4	18
5	6
6	2
7	0
More than 8	1
Average monthly household net income	Median range 3000-3250€
Diagnosed with a psychological disorder	
Yes	876
No	2551
No statement	95

Table S13. An overview of all the demographic and context variables assessed in our study.

4.2 COVID-19 specific demographics

We also collected demographic information pertaining specifically to the current SARS-CoV-2 pandemic and the related lockdown context. In our final sample, 23.74% of individuals reported belonging to COVID-19 biological risk group (e.g., due to heart disease, high blood pressure, lung disease, immunodeficiency or other risk-factors, see Figure S15). Meanwhile, 24.56% of the total sample also reported belonging to COVID-19 job-related risk group, i.e., working in a professional environment which exposed them to an increased risk for COVID-19 infection (see Figure S15). Moreover, only 1.64% individuals in our sample

reported having actually received a positive test for the COVID-19 infection. During the lockdown imposed by the German state (mid-March to mid-April 2020), 62.58% reported that they quarantined for all 4 weeks, while 18.57% claimed no isolation during the period (see Figure S16). Only 2.81% of individuals in our sample reported that they did not leave the house at all, while 75.52% left their house only to buy groceries or take a stroll (see Figure S16). On the other hand, during the re-opening period (June 2020), 47.5% claimed to have quarantined for a week or longer, and 52.5% reported not isolating at all during the period (see Figure S17). During the T3, 75.89% participants reported leaving their house for limited social activities, while less than 1% reported not leaving the house at all in the reopening period (see Figure S17). An overview of COVID-19-specific context variables in our final sample is provided in Table S14.

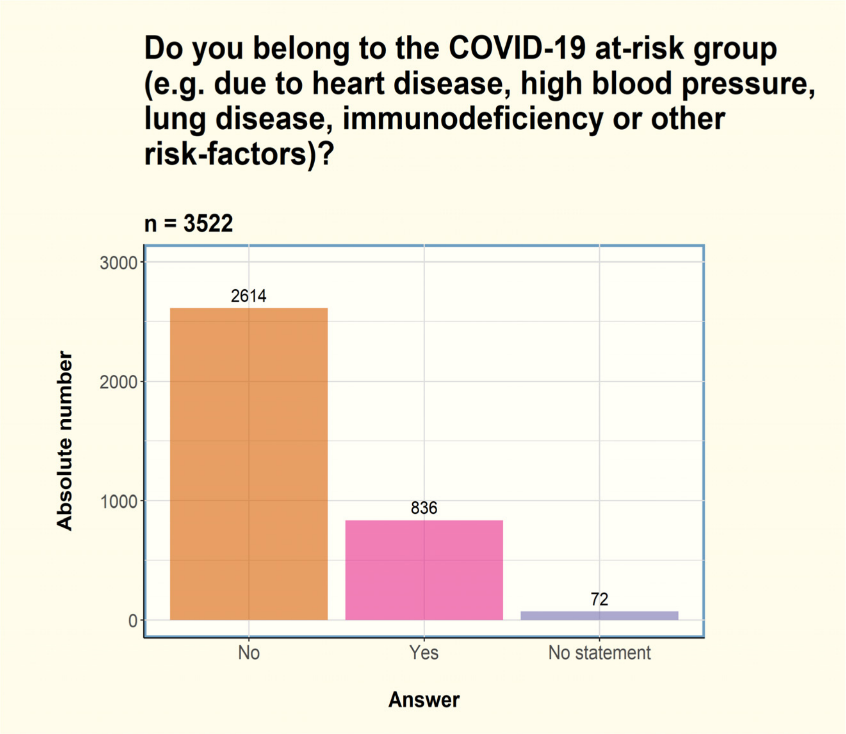

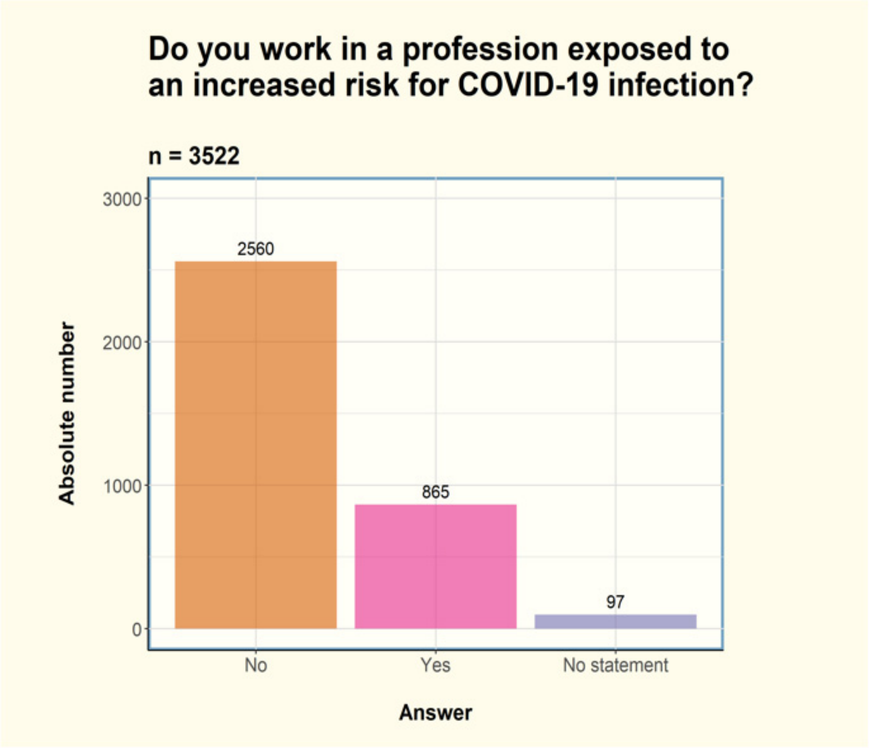

Figure S15. Top panel provides an overview of the number of participants in our sample who reported being in a biological risk group for the COVID-19 disease. Bottom panel provides an overview of the number of participants in our sample who reported being in a professional risk group for contracting COVID-19 disease.

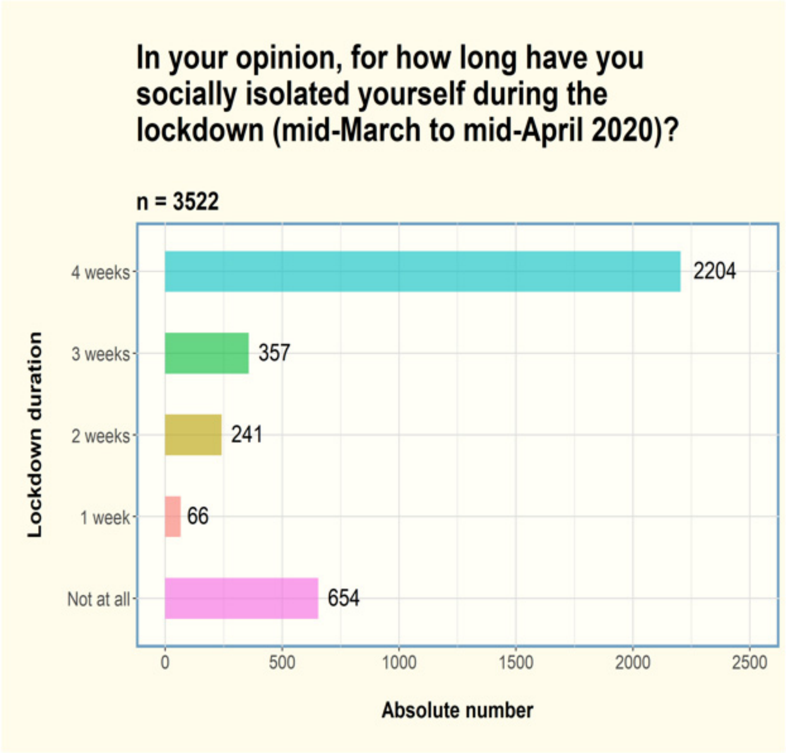

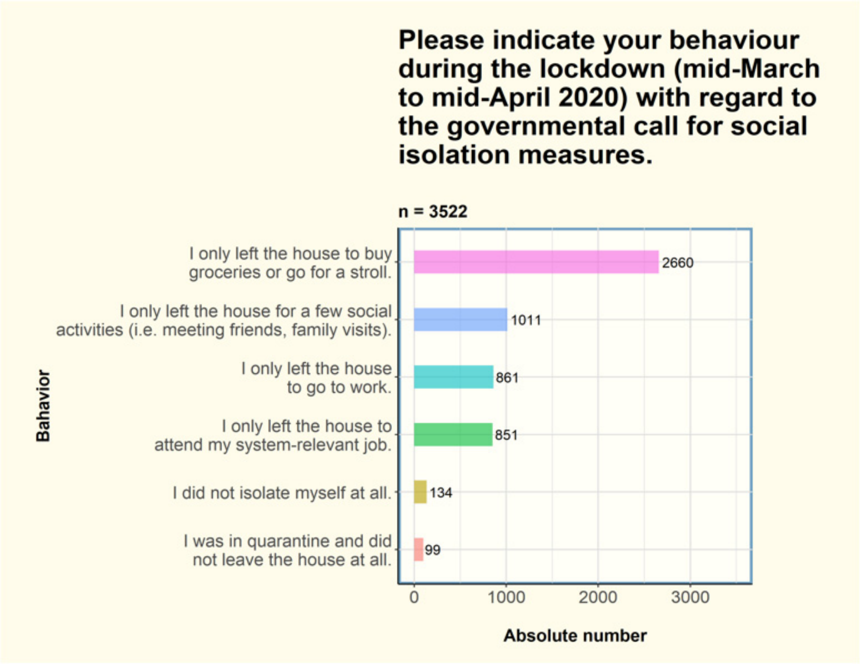

Figure S16. Top panel shows the number of weeks participants isolated during the lockdown period in Berlin (T2). The lockdown period from March 2020 to April 2020 lasted for 4 weeks in Berlin. Therefore, participants who indicated they isolated for 4 weeks essentially isolated for the entire prescribed lockdown period. Bottom panel provides an overview of the different reasons participants indicated that they left their house for during the lockdown period in Berlin (T2).

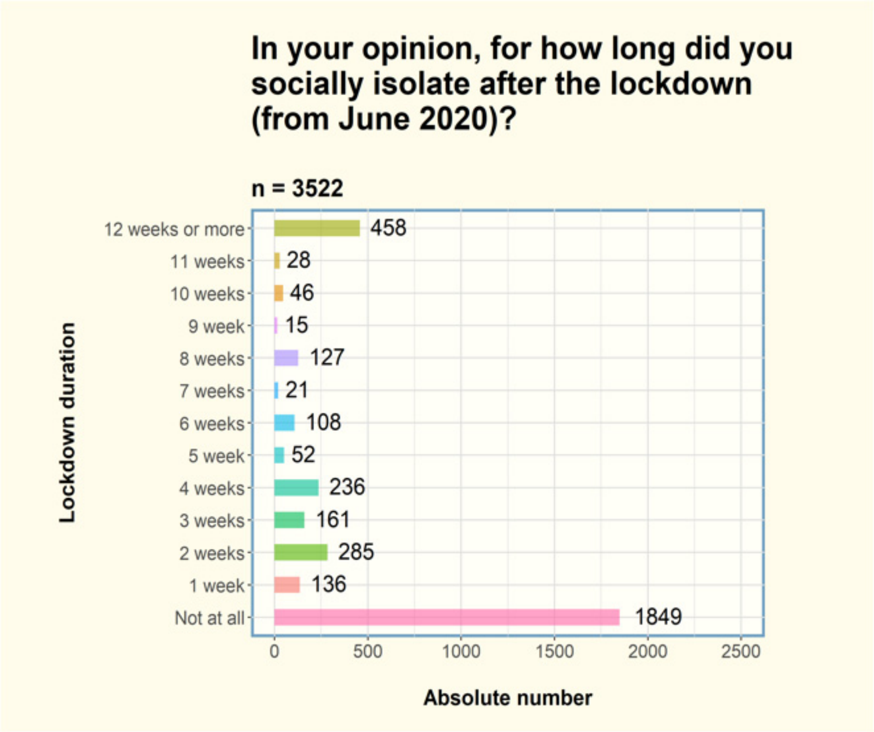

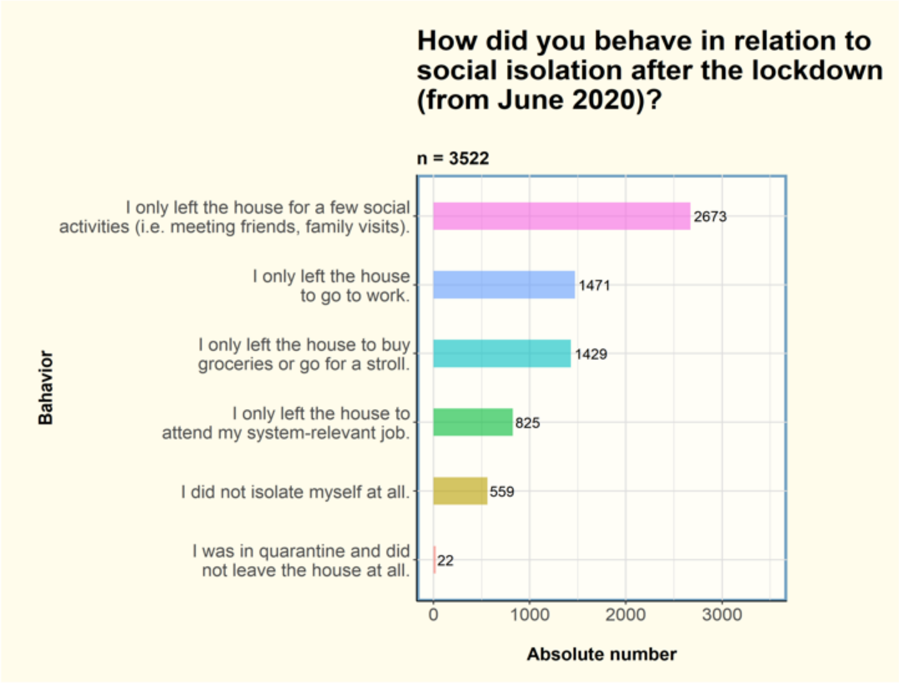

Figure S17. Top panel shows the number of weeks participants isolated during the reopening period in Berlin (T3). The reopening period was assessed as June 2020. Bottom panel provides an overview of the different reasons participants indicated that they left their house for during the reopening period in Berlin (T3).

Table S14<br>COVID-19 related questions	Total sample<br>*N* = 3522
Grouping	
Belong to COVID-19 at-risk group (e.g. due to heart disease, high blood pressure, lung disease, immunodeficiency)	
Yes	836
No	2614
No statement	72
Previously received positive COVID-19 test result through a nose or throat swap	
Yes	58
No	3464
(If yes) Subjective rating of symptom severeness	
0 No symptoms	24
1	6
2	13
3	6
4	5
5	6
6	3
7	2
8 Strong symptoms	0
(If yes) Hospitalized due to COVID-19 infection	
Yes	2
No	61
Work in profession exposed to increased risk for COVID-19 infection	
Yes	865
No	2560
No statement	97
Behaviour during the lockdown	
Did not leave the house at all	99
Only left the house for groceries, to go for a stroll	2660
Only left the house to attend system-relevant job	851
Only left the house to go to work	861
Only left the house for a few social activities	1011
No isolation at all	134
Perceived length of social isolation during lockdown (mid-March to mid-April 2020)	
Not at all	654
1 week	66
2 weeks	241
3 weeks	357
4 weeks	2204
Behaviour in relation to social isolation after the lockdown (June 2020)	
Did not leave the house at all	22
Only left the house for groceries, to go for a stroll	1429
Only left the house to attend system-relevant job	825
Only left the house to go to work	1471
Only left the house for a few social activities	2673
No isolation at all	559

Perceived length of social isolation during lockdown (mid-March to mid-April 2020)	
Not at all	1849
1 week	136
2 weeks	285
3 weeks	161
4 weeks	236
5 weeks	52
6 weeks	108
7 weeks	21
8 weeks	127
9 weeks	15
10 weeks	46
11 weeks	28
12 weeks or more	458

Table S14. An overview of the pandemic-specific demographic and context variables assessed in our study.

4.3 Sample representativeness

In order to ensure the representativeness of our final sample, we compared it to the samples of several studies conducted regarding the SARS-CoV-2 pandemic and the related lockdown. The comparison studies were conducted either in mixed international and German-speaking samples[1] or in purely international samples (Italy[2] and United States of America[3]). We also compared our sample to the demographic distribution of the Berlin population from the year 2019, obtained from the Department of Statistics, Berlin. We found our sample comparable to the samples of other COVID-19 studies in terms of mean age, and history of psychiatric diagnosis. With regards to sex distribution, our sample was

[1] Veer, I. M., Riepenhausen, A., Zerban, M., Wackerhagen, C., Puhlmann, L. M., Engen, H., ... & Kalisch, R. (2021). Psycho-social factors associated with mental resilience in the Corona lockdown. *Translational Psychiatry, 11(*1), 1-11.
[2] Gualano, M. R., Lo Moro, G., Voglino, G., Bert, F., & Siliquini, R. (2020). Effects of Covid-19 lockdown on mental health and sleep disturbances in Italy. *International Journal of Environmental Research and Public Health, 17*(13), 4779.
[3] Rozenfeld, Y., Beam, J., Maier, H., Haggerson, W., Boudreau, K., Carlson, J., & Medows, R. (2020). A model of disparities: risk factors associated with COVID-19 infection. *International Journal for Equity in Health, 19*(1), 1-10.

female-heavy, however this is also in line with the sample distributions of other COVID-19 studies which have been more female-heavy. Furthermore, our sample was favorably comparable to the demographic distribution of Berlin statistics, in terms of mean age, marital status, and family composition. Please see Figures S18A, S18B, S18C and S18D for a direct comparison of our sample to those from other studies related to the SARS-CoV-2 pandemic, and the statistics from the city of Berlin for the year 2019.

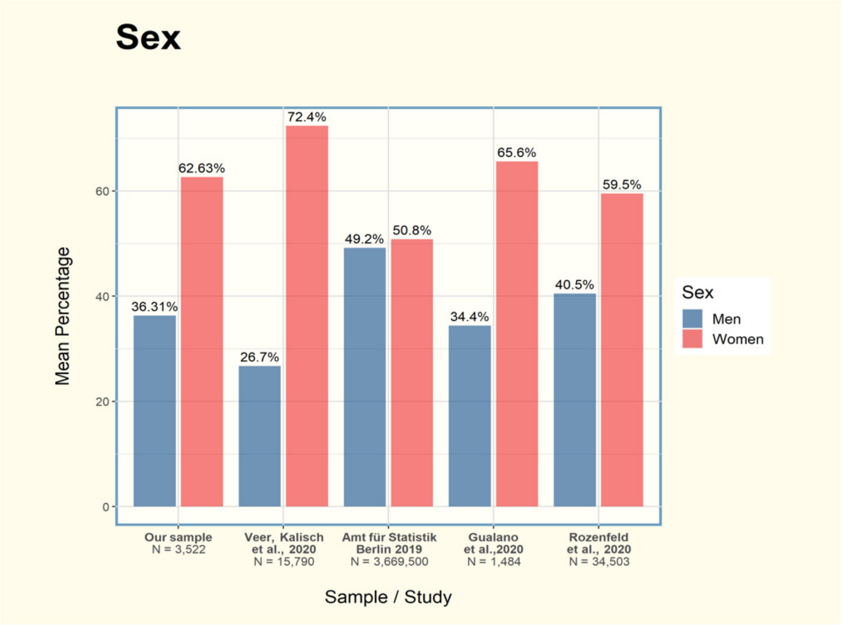

Figure S18A. Comparison of the representation of men and women in our sample with other studies and the statistics from city of Berlin in 2019.

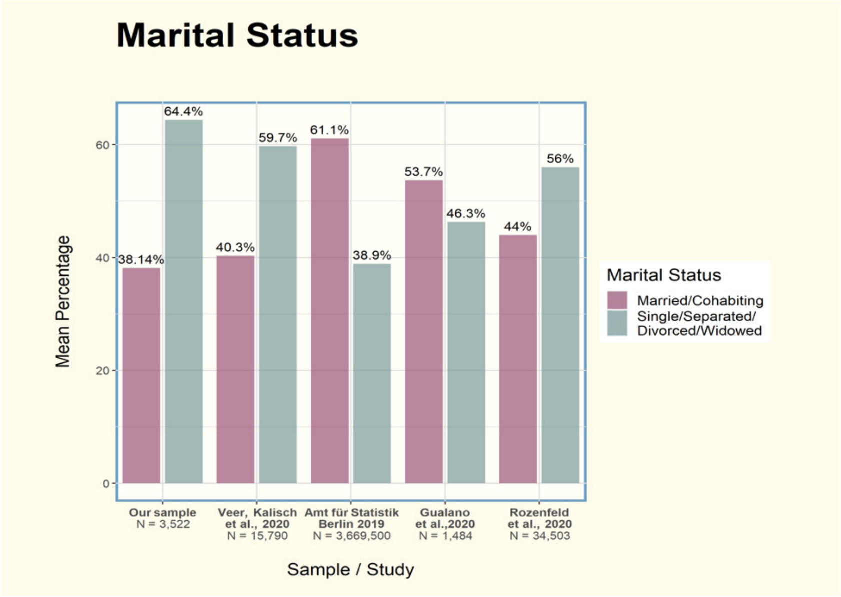

Figure S18B. Comparison of the representation of different categories of marital status in our sample with other studies and the statistics from city of Berlin in 2019.

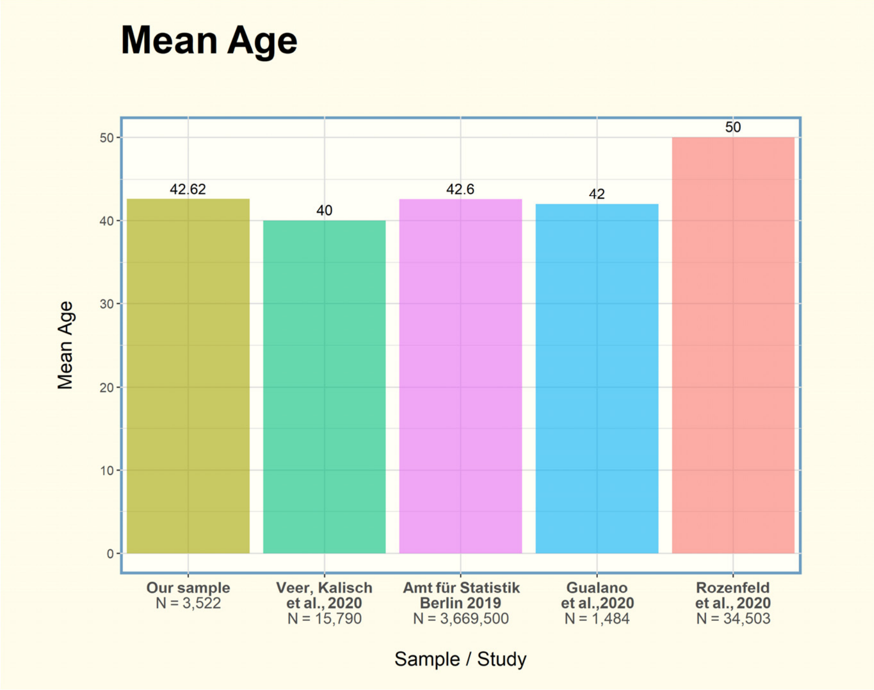

Figure S18C. Comparison of the average age in our sample with other studies and the statistics from city of Berlin in 2019.

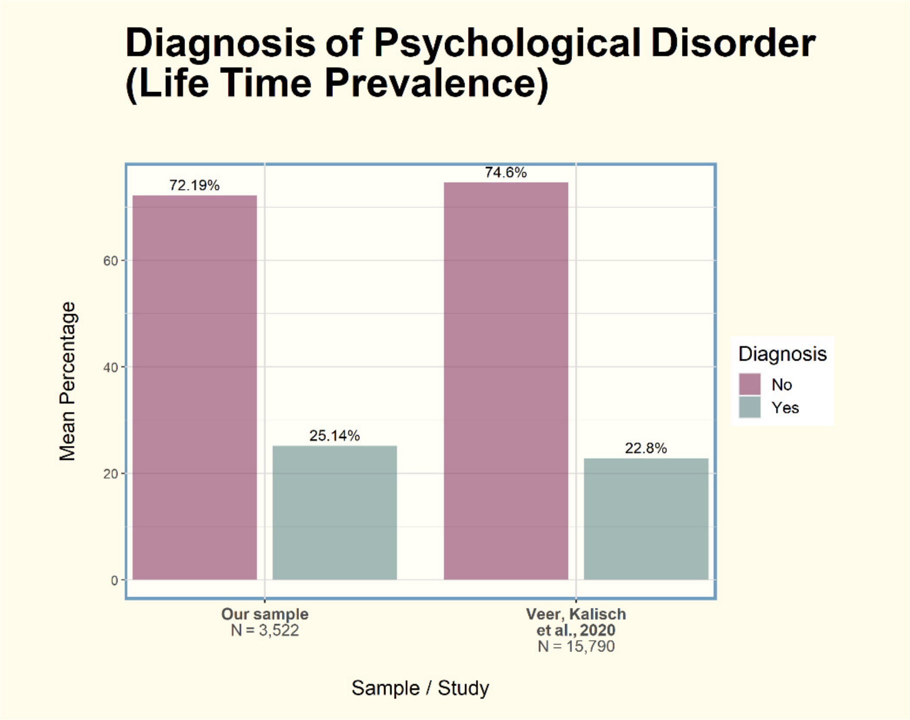

Figure S18D. Comparison of the representation of presence of psychiatric diagnosis in our sample with other studies.

5 Measures

The project used a multidisciplinary approach to assess the impact of the SARS-CoV-2 pandemic and the related lockdown on vulnerability, resilience, and social cohesion factors in our Berliner sample. We assessed two types of measures of vulnerability, resilience, and social cohesion: trait and state. As mentioned previously, participants completed the questionnaires using the webapp (www.covsocial.de). All participants completed the questionnaires in a clickable questionnaire format. Additionally, we also assessed genetic markers of vulnerability, resilience and social cohesion using saliva samples obtained from participants. However, these genetic measures and the results thereof will be discussed in subsequent publications as the genetic data is currently in processing stages.

5.1 Data protection. Central technical and organizational measures for data protection of participants were applied in accordance with the regulations of Charité – Universitätsmedizin Berlin and Max Planck Society. Personal data was stored in a pseudonymized manner, such that all personal and identifying information of the participants was separated before the storage and use of research data. Participants provided their explicit consent for storage and use of data in a pseudonymized manner on the webapp of the study. The declaration of consent and personal information of the participants were stored in electronic form on the server of Max Planck Institute for Infection Biology (MPIIB), and remain there exclusively. Participants were informed that pseudonymized data will be stored for a period of 10 years. After this period, the code key which allows the research data to be linked to the personal and identifying data will be deleted. From this time on, research data will be stored in anonymous form.

5.2 Trait measures. Trait measures of vulnerability, resilience, and social cohesion were assessed through the use of validated questionnaires. The trait measures were assessed only once throughout the study.

5.3 State measures – Psychological Variables. Several state constructs were measured using validated scales, while others were assessed through self-generated questions. The following variables of state vulnerability were assessed: emotional state (valence and arousal), alcohol use and control of alcohol use, negative news consumption, stress and perceived stress, internet consumption, compulsive internet use, craving for internet use, strains and burdens, loneliness, depressive symptoms, anxiety symptoms, psychosomatic difficulties, aggression, and perceived appropriateness of lockdown and protective measures. The following variables of state resilience were assessed: self-efficacy, optimism, seeing crisis as an opportunity (Positive Reappraisal), life satisfaction, resilience, and the use of coping strategies. The following variables of state social cohesion were assessed: social and political participation, prosocial experience and behavior, social interaction, trust, and sense of belonging. Participants completed the same state measures three times during the study for each of the three timepoints, January 2020 (Pre-lockdown period; T1), Mid-March to Mid-April 2020 (Lockdown period; T2), and June 2020 (Post-Lockdown period; T3). The state measures for T1-T3 were completed retrospectively during the period of 11 September to 7 December 2020. Therefore, in order to ensure that participants were completely mentally immersed into the timeframe of the particular timepoint of interest, they underwent a brief perspective-taking exercise prior to responding to the state-related measures. Prior to each block of the state questionnaires, participants were asked to take a moment to recollect what was happening during the

timepoint. They were reminded of the major *world* events taking place at that particular timepoint, and were asked to recall the major *individual* life events taking place during that time. They were also asked to consult their planners, schedules and agendas to jog their memory of the daily life events taking place during the period, and to immerse themselves in the perspective of the particular timepoint. For example, prior to completing the state measures for T1, participants were reminded about the recent Christmas and New Year's Eve celebrations of 2019, major local Berlin events such as arrival of the baby panda bear twins at the Berlin Zoo, and world events such as World Economic Forum at Davos and the Australian bushfires of January 2020. They were then asked to consult their personal planners for the month of January 2020 to remind themselves of the daily events taking place in their lives during that month. Once the participants had appropriately immersed themselves into the perspective of the timepoint, they could then begin completing the state measures from that perspective. We must reiterate that study also longitudinally assessed state-level measures during the period of second lockdown in Berlin (November 2020 – April 2021, T4-T7). However, due to the fact that the complete dataset has not been obtained yet, the results from T4-T7 timepoints will be made available in subsequent publications.

5.4 State Measures – COVID-19 Specific Variables. Given the unique impact of the COVID-19 pandemic and the related lockdown on certain, specific aspects of individual life, we also assessed the impact on these covid-specific in our Berlin sample. This was done in order to examine whether changes in these covid-specific variables over the three timepoints impacted the state measures of vulnerability, resilience, and social cohesion on one timepoint or over timepoints. The covid-specific variables were also measured for each of the three timepoints T1, T2 and T3, and they were assessed only through self-generated

questions. The following covid-specific variables were assessed: living at regular place of residence, number and type of co-residents, time spent outdoors, number of working hours, changes in workload, amount of time spent working in home office and at workplace, perceived financial security, financial ability to cover basic needs, covid-related anxiety, covid-specific fears (such as running out of food or toilet paper, losing job, contracting diseases or viruses, etc.), and covid-specific behaviors (such as stocking up on food, stocking up on toilet paper, withdrawing large sums of money, etc.).

6 Results

In this chapter we provide the descriptive results of the trait and state measures of vulnerability, resilience and social cohesion, and the state measures of COVID-19 specific variables used in our study.

6.1 Trait Measures. Please see Table S15 below for descriptives related to the trait questionnaires assessed in our sample. Figure S17 provides an overview of the correlation between the trait variables of interest.

Measure	Questionnaire	Measure	Mean (95% CI)
Vulnerability	State-Trait Anxiety Inventory	Anxiety	39.93 (39.59/40.28)
	Neuroticism-Extraversion-Openness	Neuroticism	20 (19/20)
	Trier Inventory Chronic Stress	Stress	17.42 (17.07/17.77)
	UCLA Loneliness Scale	Loneliness	1.9 (1.9/2.0)
	Cognitive Emotion Regulation Questionnaire	Self-blame	3.9 (3.8/4.0)
		Other-blame	2.7 (2.7/2.8)
		Catastrophizing	2.6 (2.6/2.7)
	Life Orientation test	Pessimism	4.0 (4.0/4.1)
Resilience	Brief Resilience Scale	Resilience	3.4 (3.4/3.4)
	Satisfaction with Life Scale	Life satisfaction	25 (25/25)
	Life Orientation test	Optimism	8.3 (8.2/8.4)
	Self-Compassion scale	Self-kindness	6.2 (6.2/6.3)
		Common Humanity	6.0 (6.0/6.1)
		Mindfulness	7.3 (7.2/7.3)
	Coping Orientation to Problems Experienced	Deflection	3.38 (3.33/3.43)
		Emotional support	3.24 (3.19/3.29)
		Positive reinterpretation	3.39 (3.34/3.44)
		Humor	2.7 (2.64/2.75)
		Exercise	3.49 (3.44/3.54)
		Instrumental support	2.64 (2.59/2.69)
		Act on emotions	2.34 (2.3/2.39)
		Planning	3.86 (3.81/3.9)
		Acceptance	3.28 (3.23/3.33)
		Religion	0.86 (0.81/0.91)
	Big Five Inventory	Extraversion	3.2 (3.2/3.2)
		Agreeableness	3.2 (3.2/3.2)
		Conscientiousness	3.7 (3.7/3.7)
		Openness	3.8 (3.8/3.8)
Social Cohesion	General Trust Scale	Trust	2.5 (2.5/2.5)
	Berlin social support scale	Social support	3.5 (3.5/3.5)
	Social Cohesion Prosocialness Scale	Prosocialness	3.6 (3.5/3.6)

	Interpersonal Reactivity Index	Empathic concern	19.76 (19.61/19.91)
		Perspective taking	18.39 (18.24/18.54)

Table S15. An overview of the variables assessed for trait vulnerability, resilience, and social cohesion. The name of the validated questionnaire used is provided, along with the construct of interest measured by the questionnaire in our study, the associated subscales used, and the mean and 95% confidence interval.

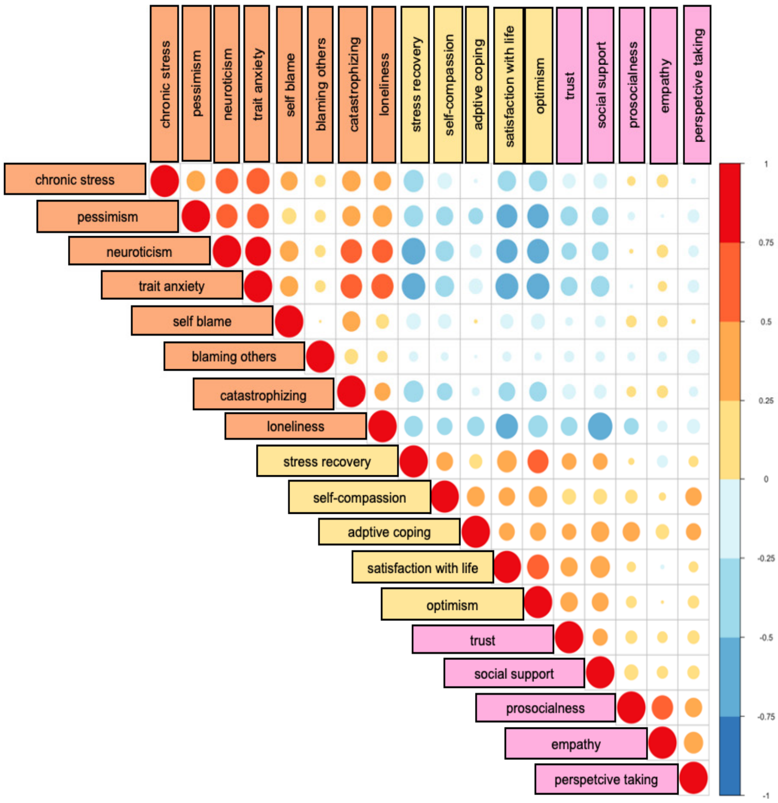

Figure S17. An overview of the correlations between the different measures of trait vulnerability (orange-colored variables), resilience (yellow-colored variables), and social cohesion (pink colored variables). The bigger the circle indicates stronger the magnitude of correlation, and the color of the circle indicates whether the correlation is positive or negative.

6.2 State Measures - Psychological Variables

6.2.1 State Vulnerability. Compared to pre-lockdown (T1) and re-opening (T3), during lockdown period participants reported elevated median levels of loneliness (Figure S20), stress and perceived stress (Figure S21), negative news consumption (Figure S22), depression (Figure S23), and anxiety (Figure S24). However, participants did not report any

numerical changes, from T1 to T2 and T2 to T3, in the following variables: median levels of alcohol use as measured through AUDIT (Figure S25), median levels of control of alcohol use (Figure S25), mean levels of aggression as perpetrator (Figure S26), or mean levels of being a victim of aggression (Figure S27). We found overall numerical increases in financial burdens (Figure S28), mental and physical health burdens (Figure S29), burdens created by interpersonal stresses or conflicts (Figure S30), and in burdens resulting from limitations imposed by the lockdown (Figure S31). Further, participants reported no numerical differences in burdens related to discriminatory behavior (Figure S32). We found a numerical elevation in internet consumption for various activities (Figure S33), and a corresponding numerical increase in craving or desire for internet consumption (Figure S34). However, participants did not report elevations in levels of compulsive internet use during the lockdown phase (Figure S35). Furthermore, we found a numerical increase in reporting of several different categories of psychosomatic behaviors, but surprisingly a decline in reporting of psychosomatic cold symptoms (Figure S36). Lastly, participants reported a negative emotional state during the lockdown (Figure S37), and a numerical increase in emotional arousal (Figure S38).

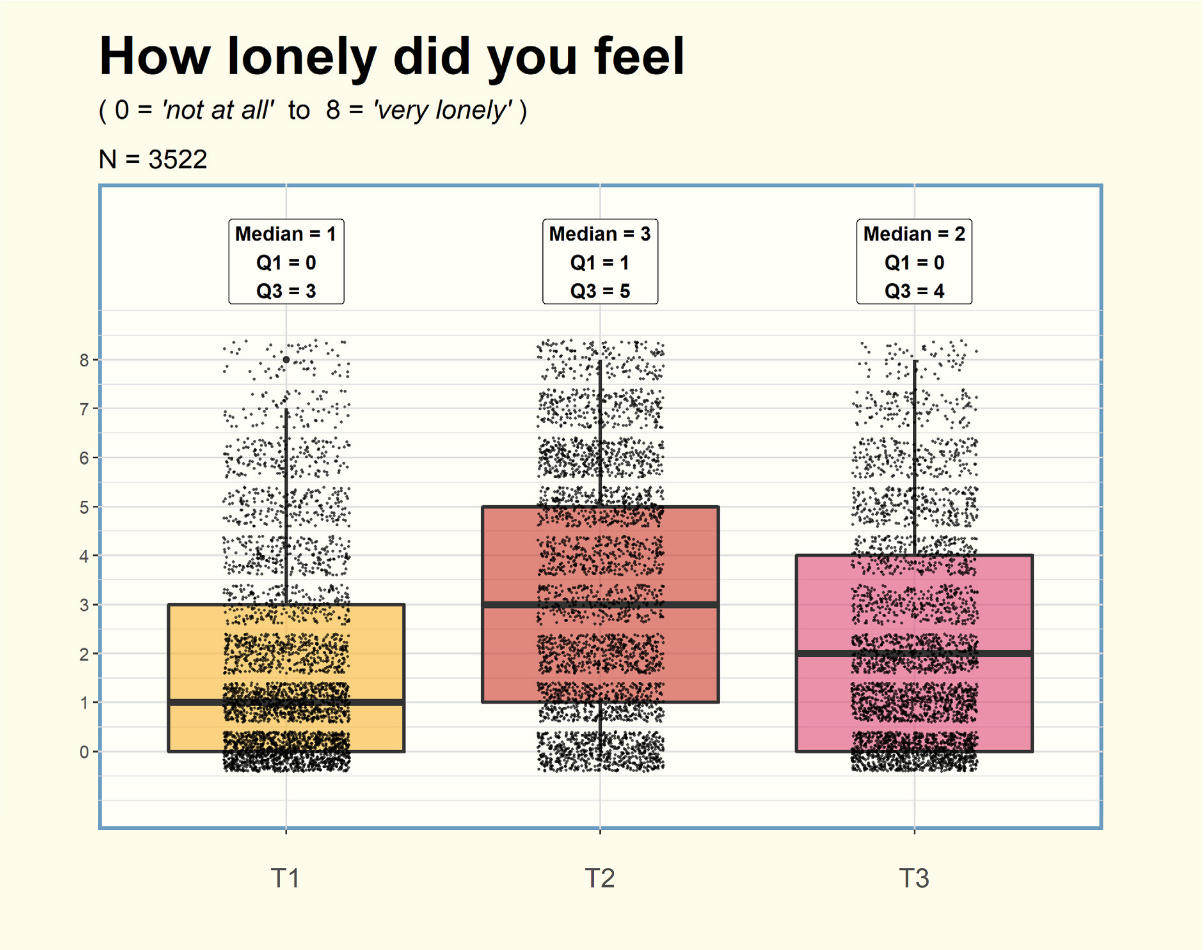

Figure S20. Median levels of loneliness for the three timepoints T1 (Pre-Lockdown), T2 (Lockdown), and T3 (Re-opening). Error bars indicate interquartile range. Q1 = Lower Quartile, Q2 = Upper Quartile.

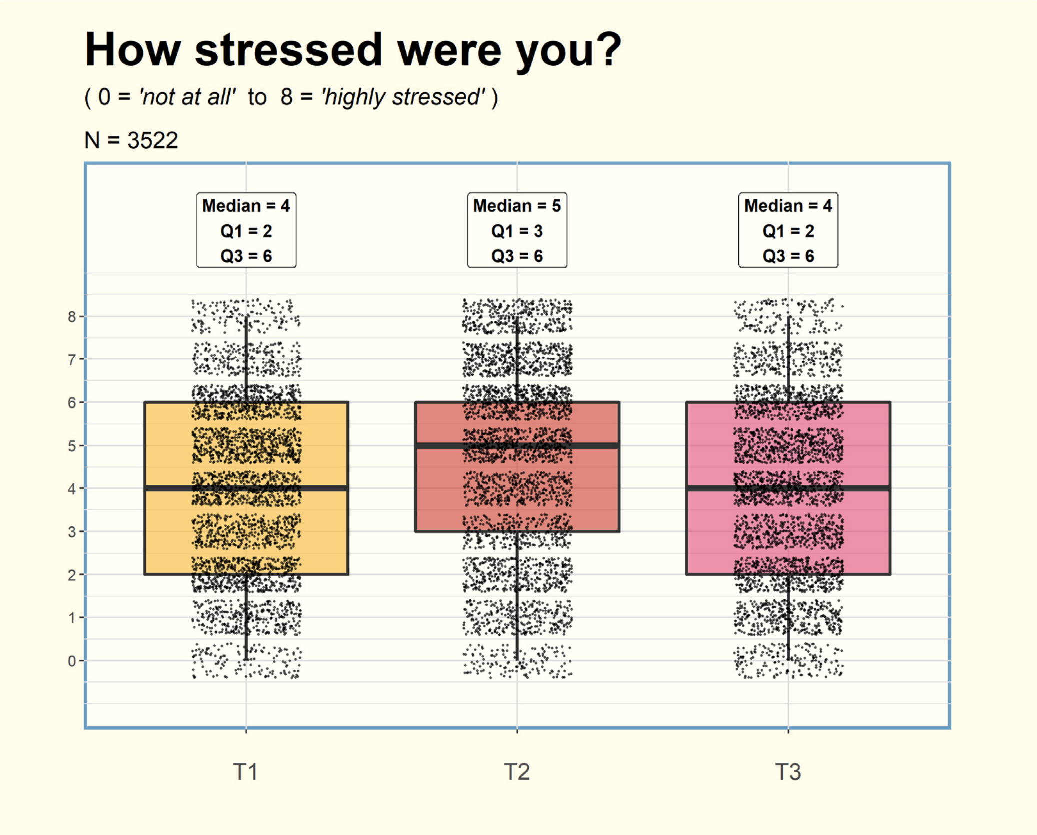

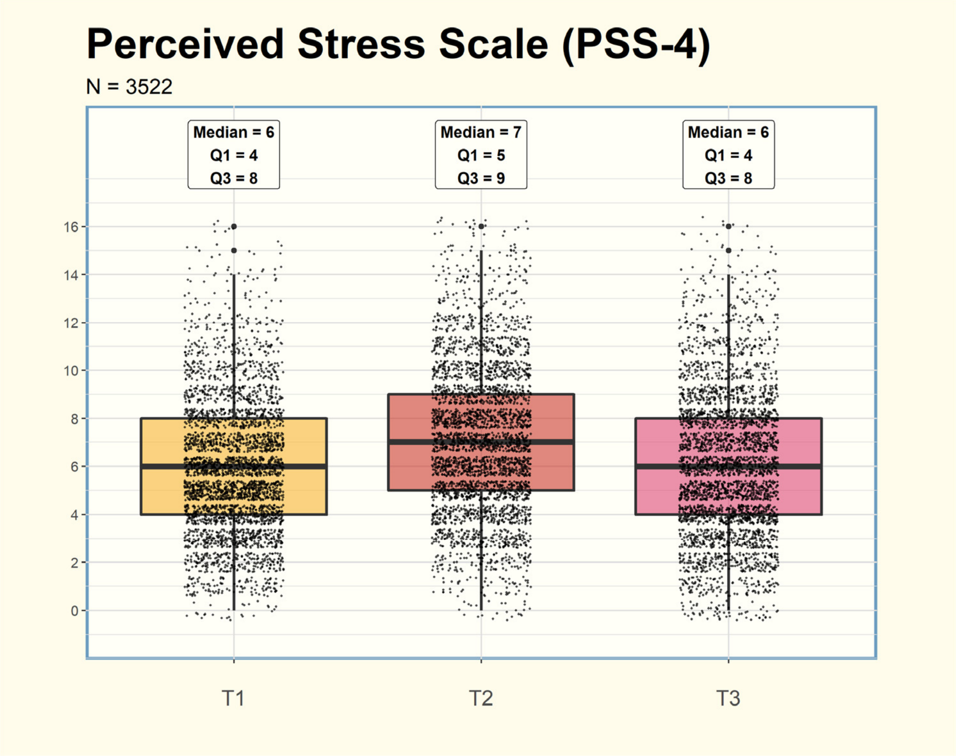

Figure S21. Median levels of stress (top panel) and perceived stress using PSS-4 (bottom panel) for the three timepoints T1 (Pre-Lockdown), T2 (Lockdown), and T3 (Re-opening). Error bars indicate interquartile range. Q1 = Lower Quartile, Q2 = Upper Quartile.

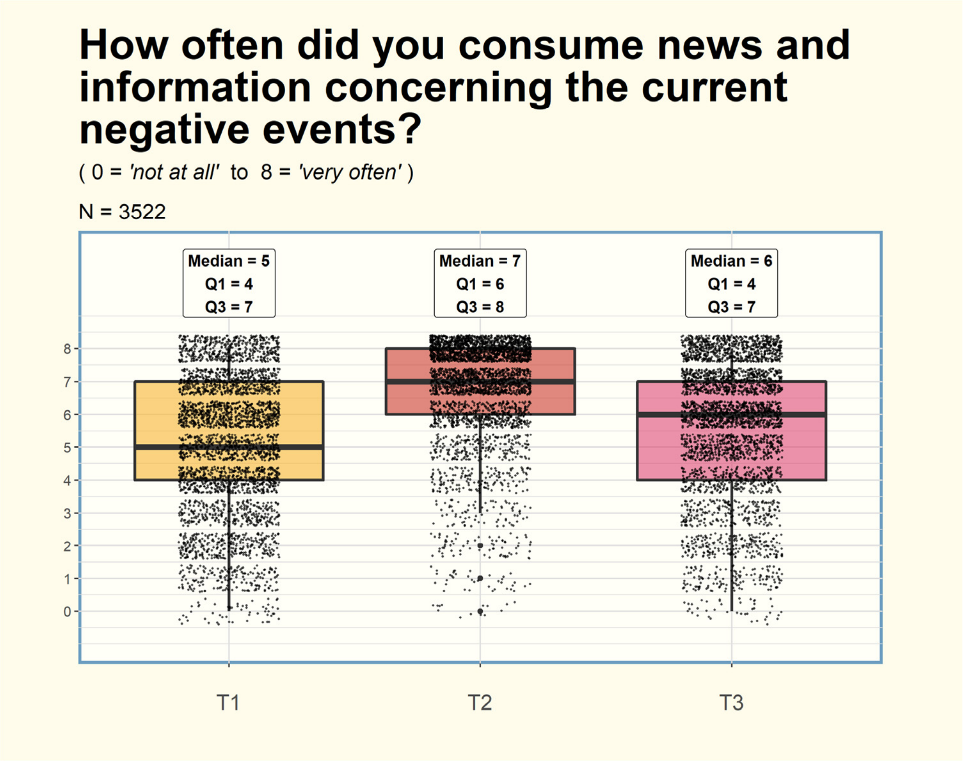

Figure S22. Median levels of negative news consumption for the three timepoints T1 (Pre-Lockdown), T2 (Lockdown), and T3 (Re-opening). Error bars indicate interquartile range. Q1 = Lower Quartile, Q2 = Upper Quartile.

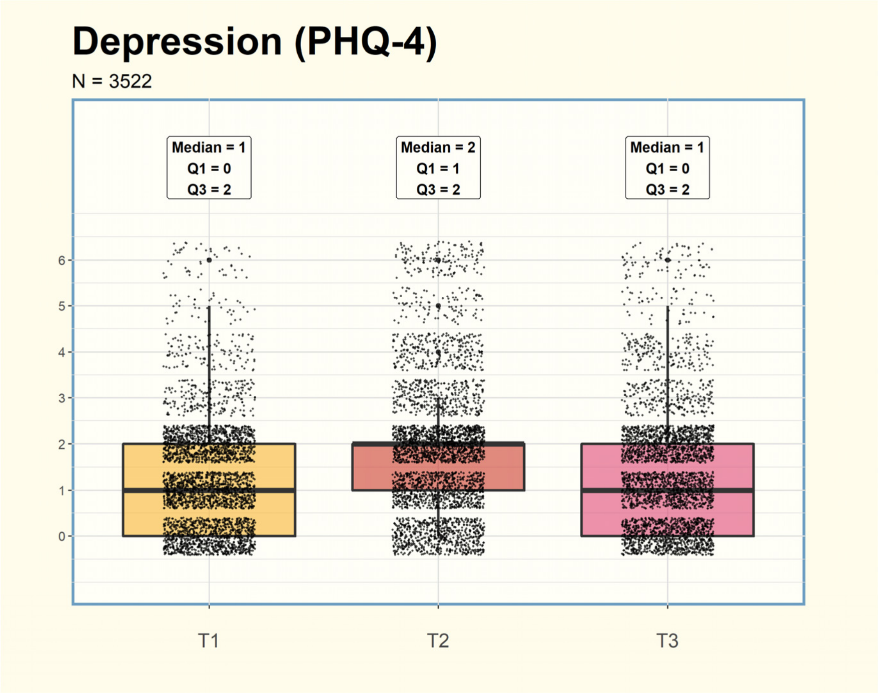

Figure S23. Median levels of depressive symptoms for the three timepoints T1 (Pre-Lockdown), T2 (Lockdown), and T3 (Re-opening). Error bars indicate interquartile range. Q1 = Lower Quartile, Q2 = Upper Quartile.

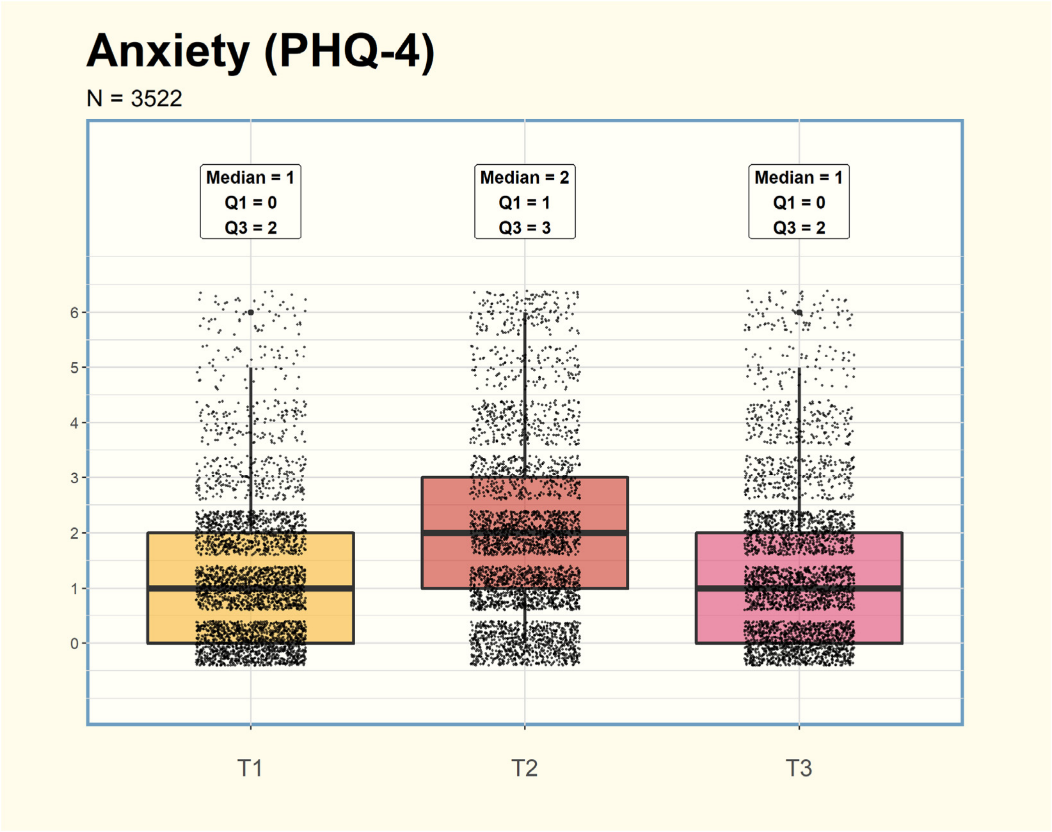

Figure S24. Median levels of anxious symptoms for the three timepoints T1 (Pre-Lockdown), T2 (Lockdown), and T3 (Re-opening). Error bars indicate interquartile range. Q1 = Lower Quartile, Q2 = Upper Quartile.

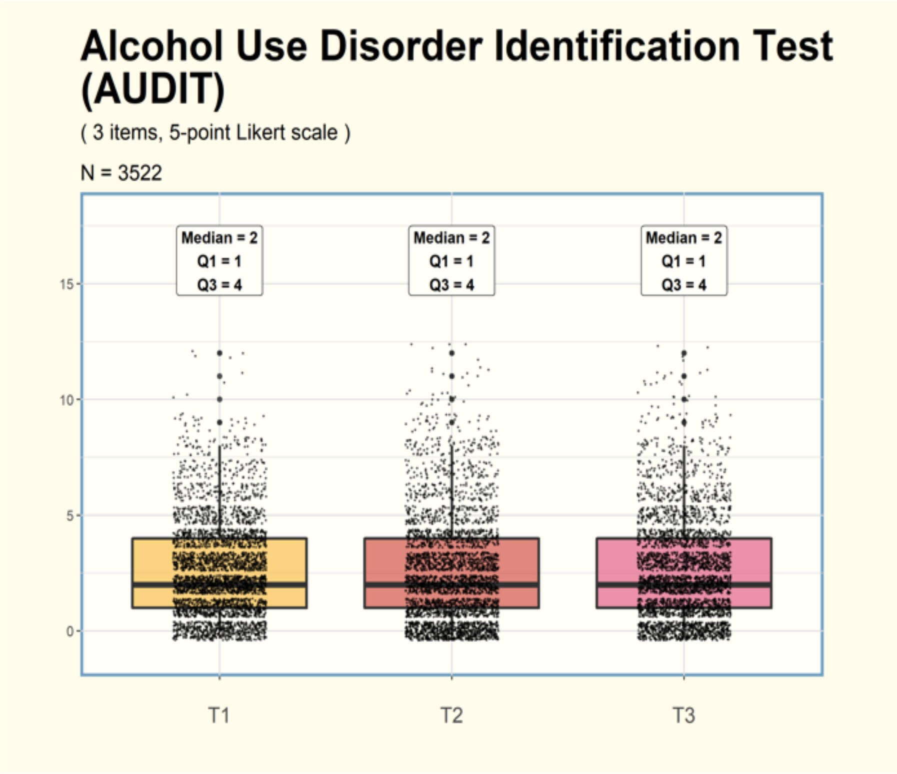

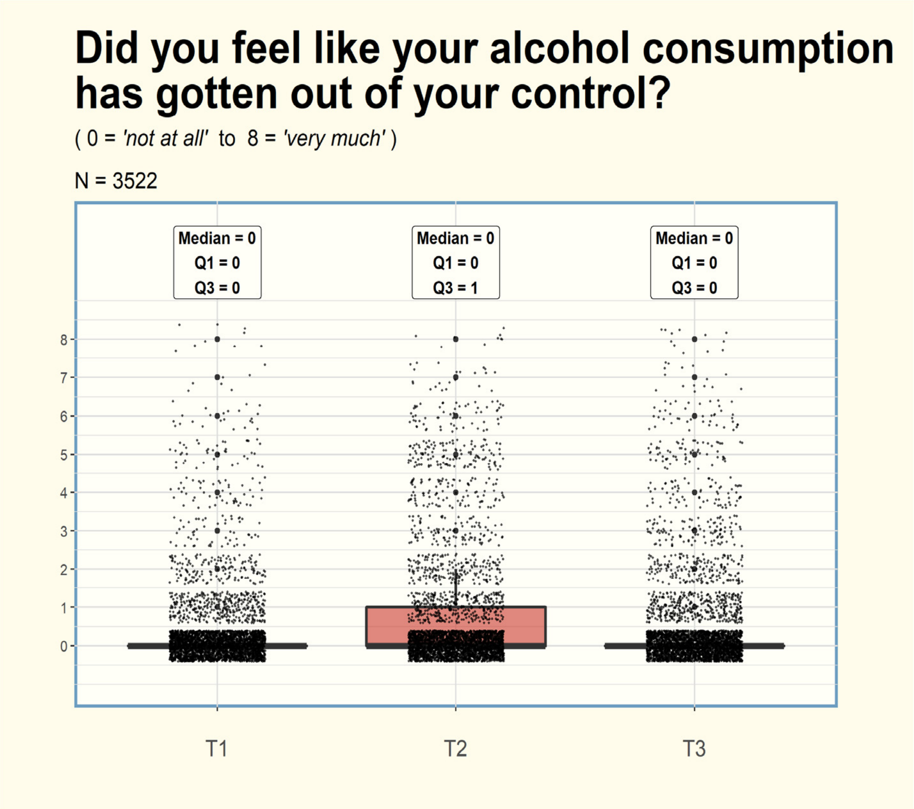

Figure S25. Median levels of alcohol use (top panel) and control of alcohol use (bottom panel) for the three timepoints T1 (Pre-Lockdown), T2 (Lockdown), and T3 (Re-opening). Error bars indicate interquartile range. Q1 = Lower Quartile, Q2 = Upper Quartile.

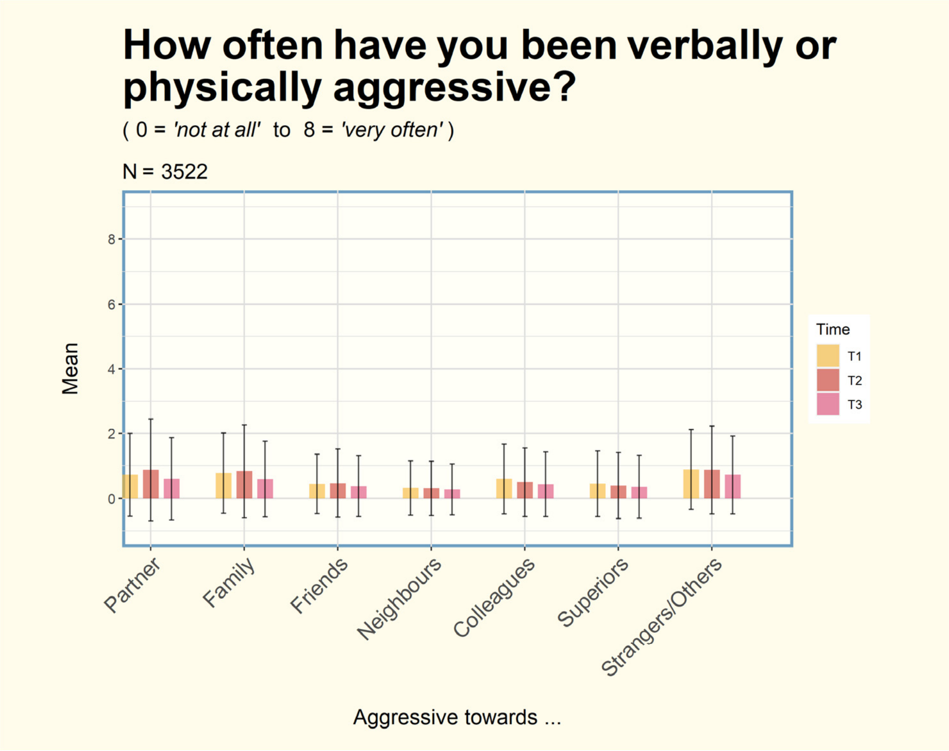

Figure S26. Mean levels of aggression as perpetrator for the three timepoints T1 (Pre-Lockdown), T2 (Lockdown), and T3 (Re-opening). Error bars indicate standard deviation.

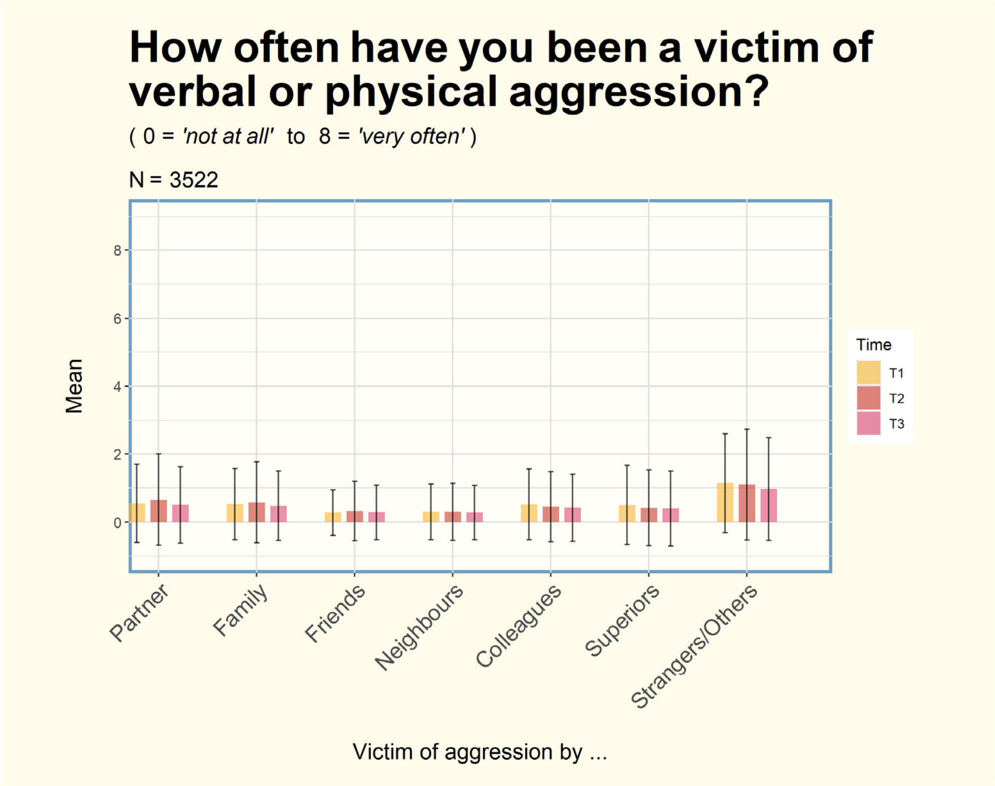

Figure S27. Mean levels of aggression as a victim for the three timepoints T1 (Pre-Lockdown), T2 (Lockdown), and T3 (Re-opening). Error bars indicate standard deviation.

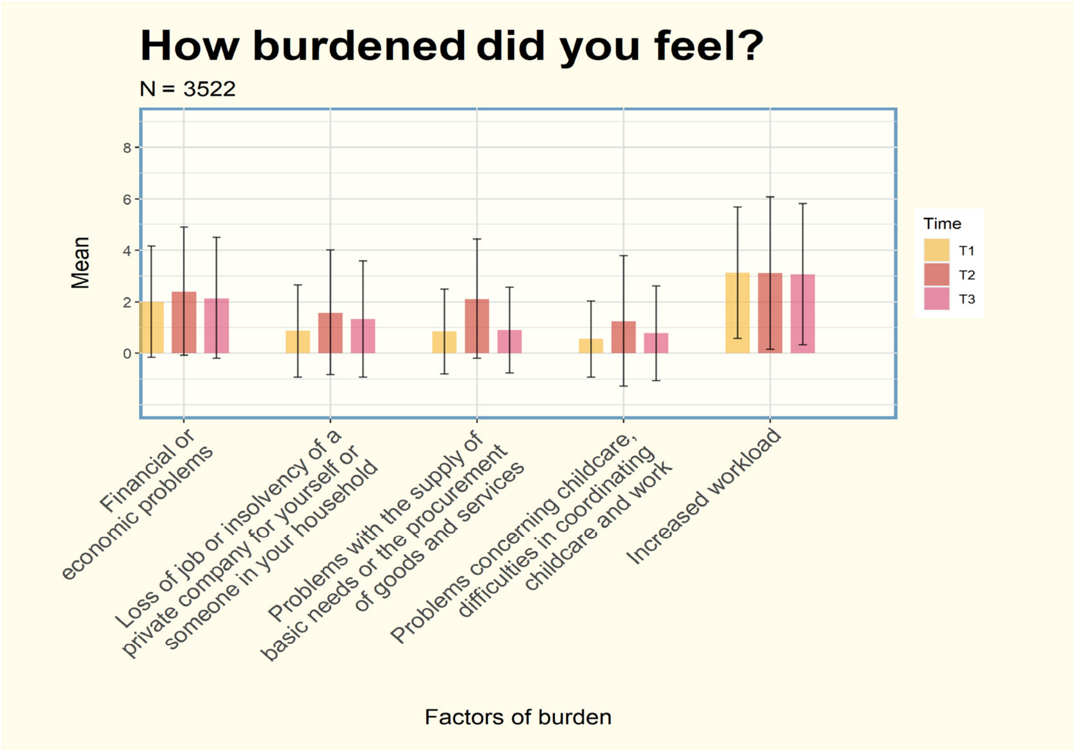

Figure S28. Mean levels of finance-related burdens for the three timepoints T1 (Pre-Lockdown), T2 (Lockdown), and T3 (Re-opening). Error bars indicate standard deviation.

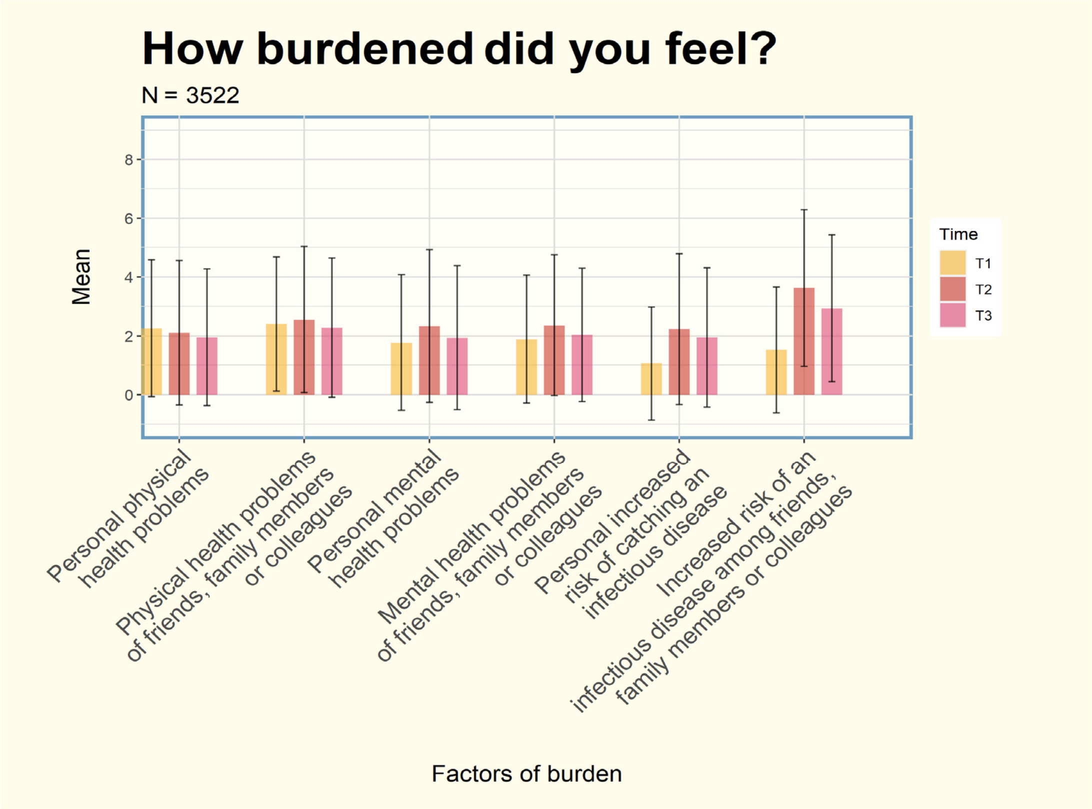

Figure S29. Mean levels of physical and mental health -related burdens for the three timepoints T1 (Pre-Lockdown), T2 (Lockdown), and T3 (Re-opening). Error bars indicate standard deviation.

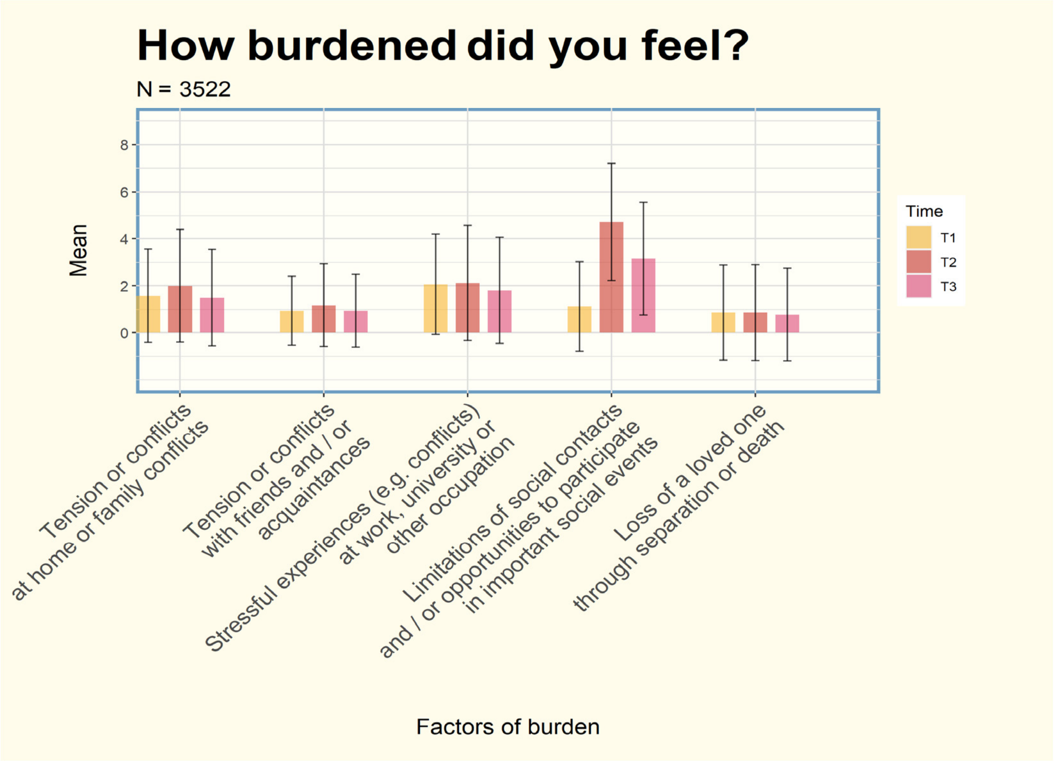

Figure S30. Mean levels of conflict-related burdens for the three timepoints T1 (Pre-Lockdown), T2 (Lockdown), and T3 (Re-opening). Error bars indicate standard deviation.

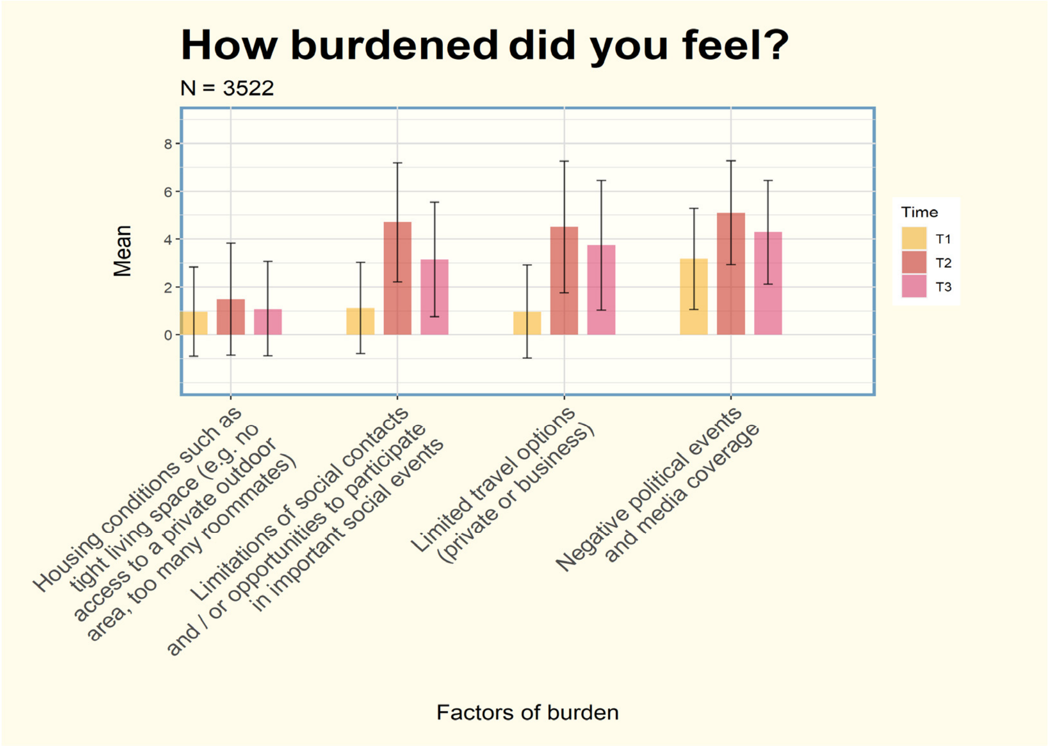

Figure S31. Mean levels of limitation-related burdens for the three timepoints T1 (Pre-Lockdown), T2 (Lockdown), and T3 (Re-opening). Error bars indicate standard deviation.

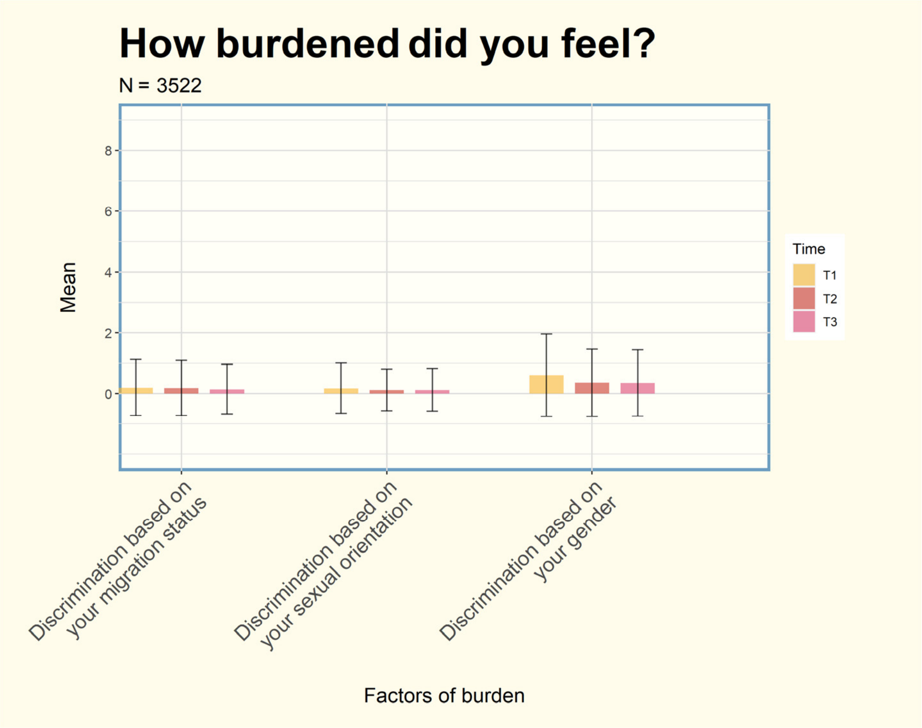

Figure S32. Mean levels of discrimination-related burdens for the three timepoints T1 (Pre-Lockdown), T2 (Lockdown), and T3 (Re-opening). Error bars indicate standard deviation.

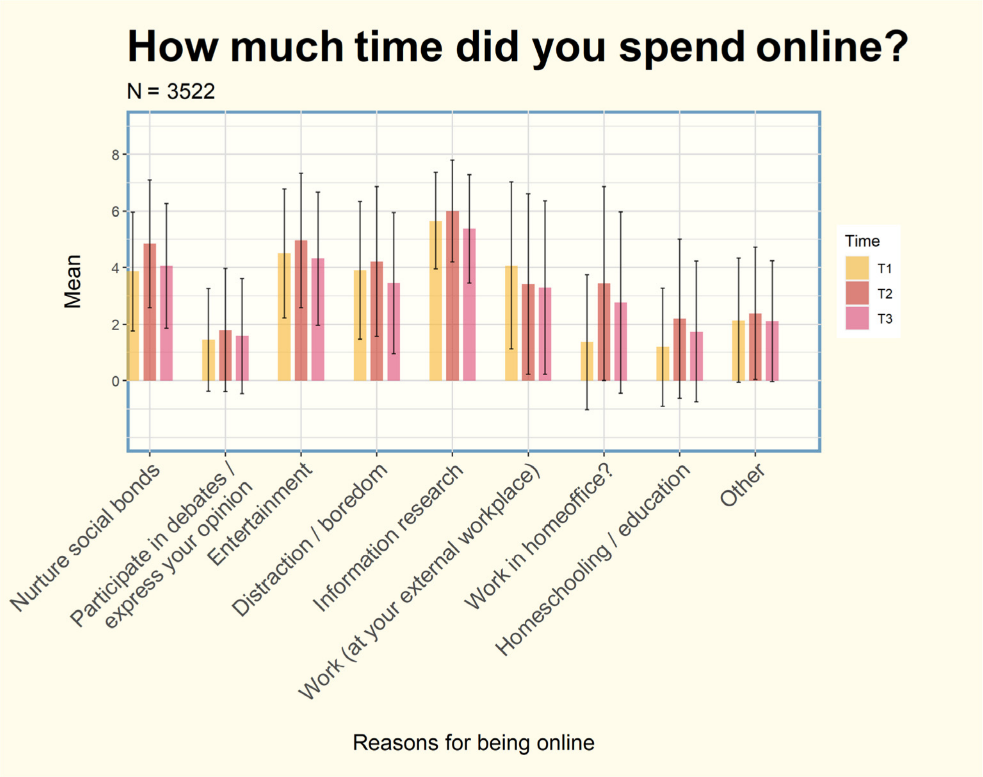

Figure S33. Mean levels of internet consumption for various activities for the three timepoints T1 (Pre-Lockdown), T2 (Lockdown), and T3 (Re-opening). Error bars indicate standard deviation.

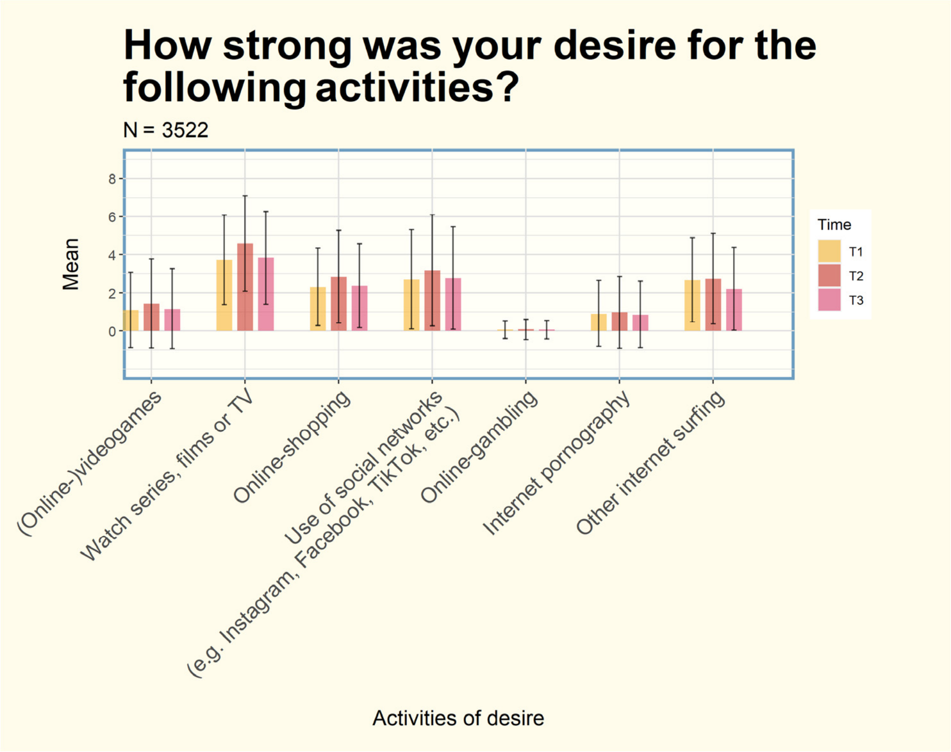

Figure S34. Mean levels of desire for internet consumption for various activities for the three timepoints T1 (Pre-Lockdown), T2 (Lockdown), and T3 (Re-opening). Error bars indicate standard deviation.

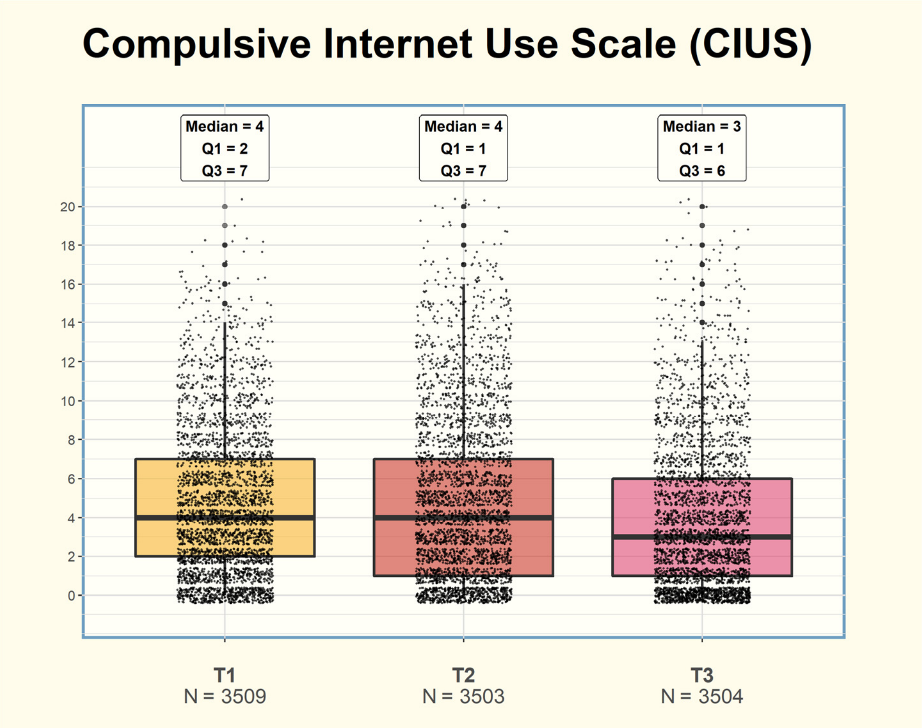

Figure S35. Median levels of compulsive internet use for the three timepoints T1 (Pre-Lockdown), T2 (Lockdown), and T3 (Re-opening). Error bars indicate interquartile range. Q1 = Lower Quartile, Q2 = Upper Quartile.

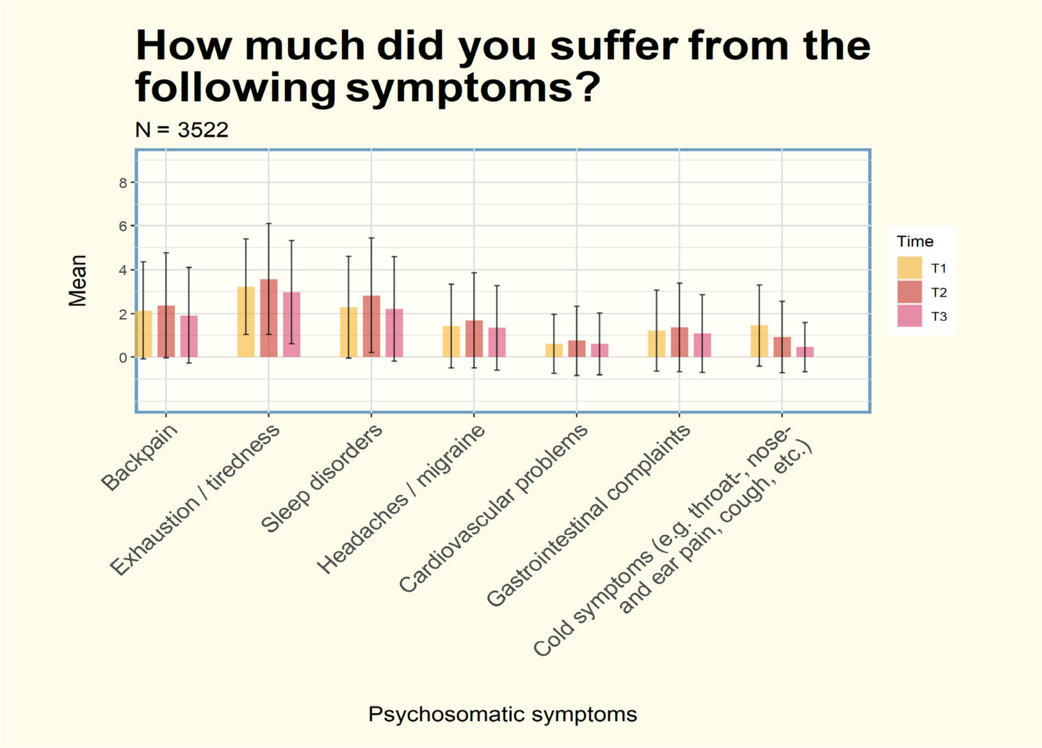

Figure S36. Mean levels of psychosomatic symptoms reported by participants for the three timepoints T1 (Pre-Lockdown), T2 (Lockdown), and T3 (Re-opening). Error bars indicate standard deviation.

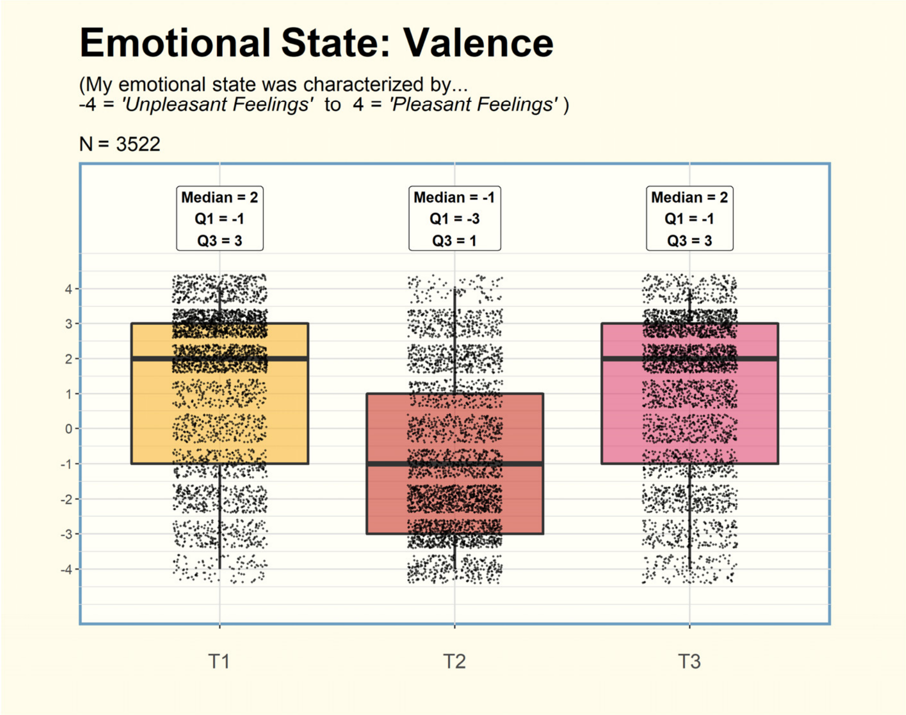

Figure S37. Median levels of valence of emotional state for the three timepoints T1 (Pre-Lockdown), T2 (Lockdown), and T3 (Re-opening). Error bars indicate interquartile range. Q1 = Lower Quartile, Q2 = Upper Quartile.

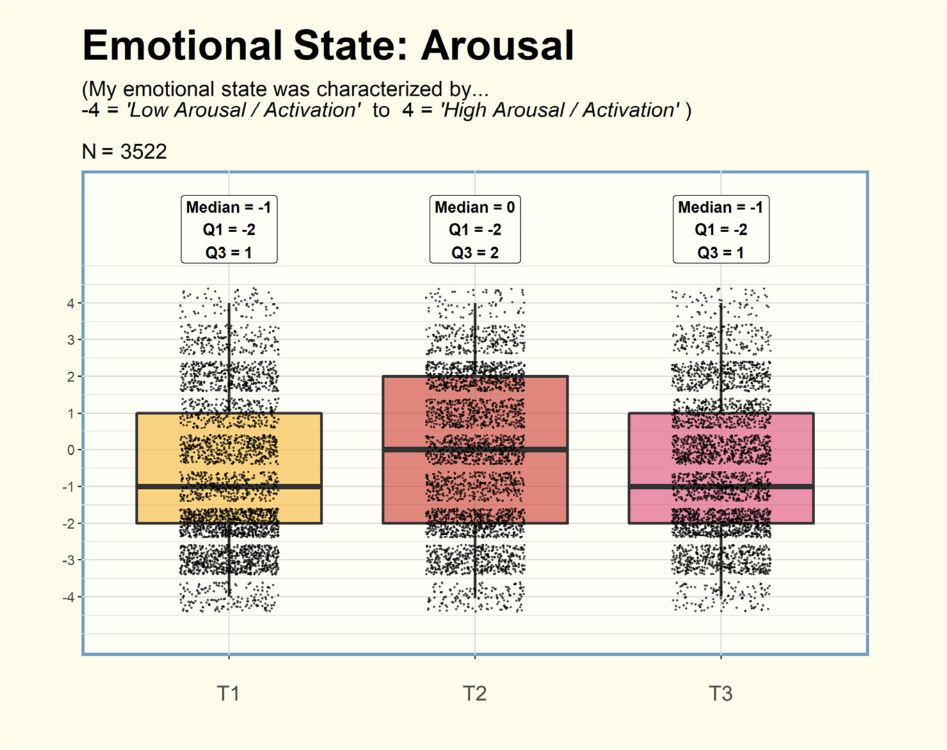

Figure S38. Median levels of emotional arousal for the three timepoints T1 (Pre-Lockdown), T2 (Lockdown), and T3 (Re-opening). Error bars indicate interquartile range. Q1 = Lower Quartile, Q2 = Upper Quartile.

6.2.2 State Resilience. Compared to the pre-lockdown period (T1) and reopening period (T3), during lockdown period, we found numerical decreases in median levels of life satisfaction (Figure S39), optimism (Figure S40), resilience (Figure S41), self-efficacy (Figure S42), and mean levels of seeing crisis as opportunity (Figure S43). During lockdown, participants reported the following coping strategy use was increased compared to T1: spending time in nature, acceptance, and distraction (Figure S44).

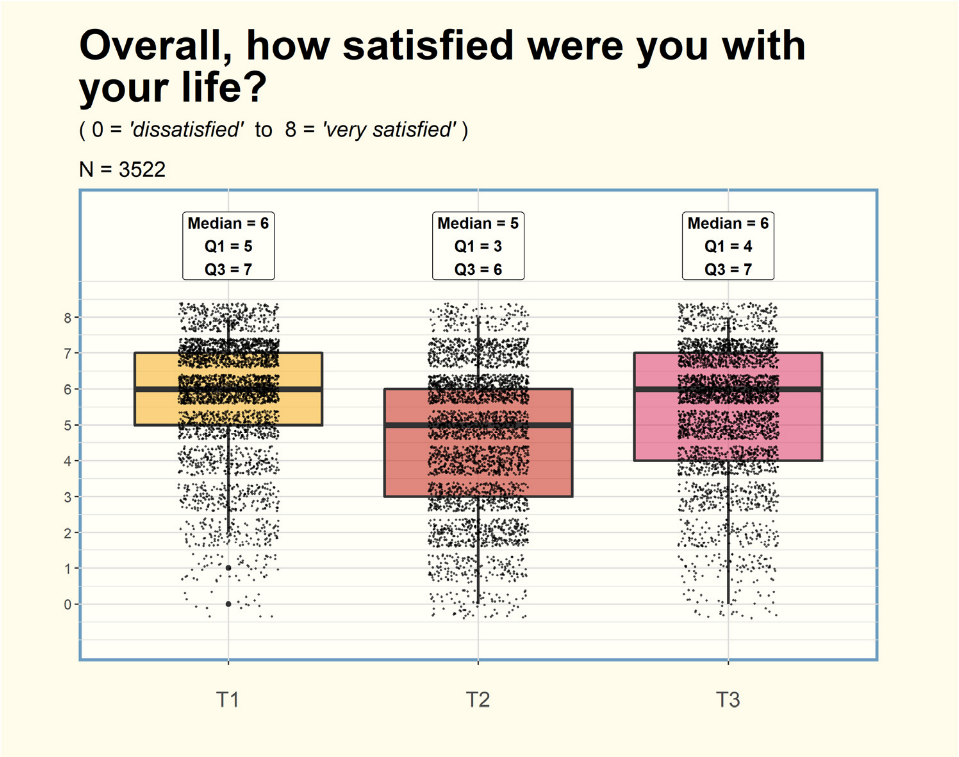

Figure S39. Median levels of life satisfaction for the three timepoints T1 (Pre-Lockdown), T2 (Lockdown), and T3 (Re-opening). Error bars indicate interquartile range. Q1 = Lower Quartile, Q2 = Upper Quartile.

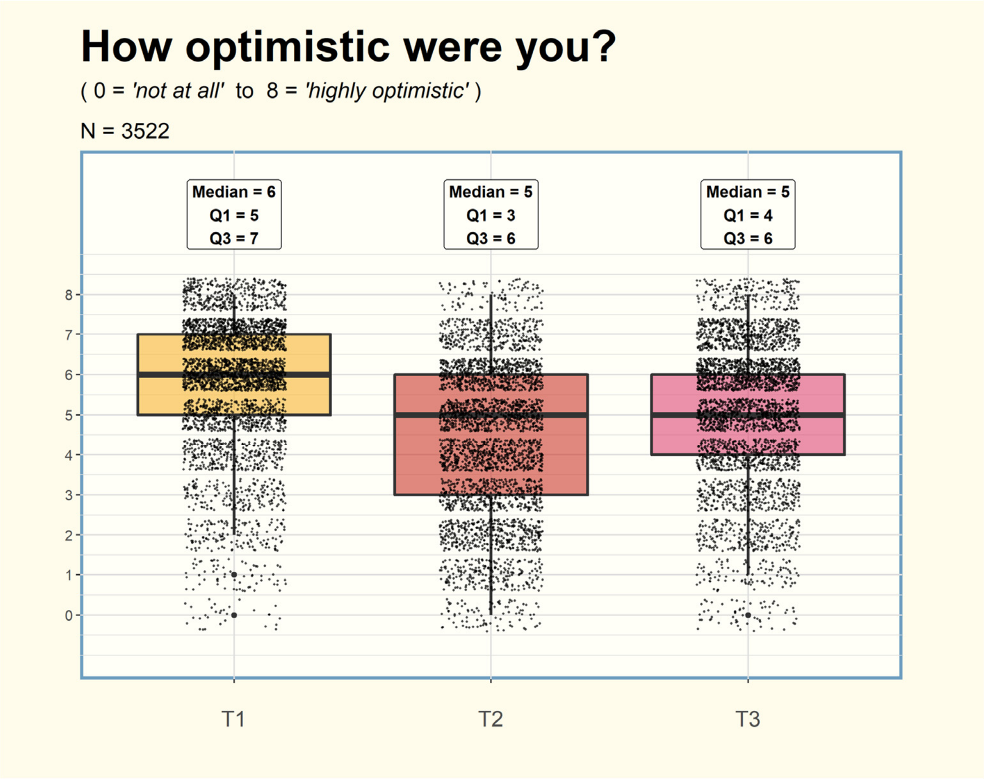

Figure S40. Median levels of optimism for the three timepoints T1 (Pre-Lockdown), T2 (Lockdown), and T3 (Re-opening). Error bars indicate interquartile range. Q1 = Lower Quartile, Q2 = Upper Quartile.

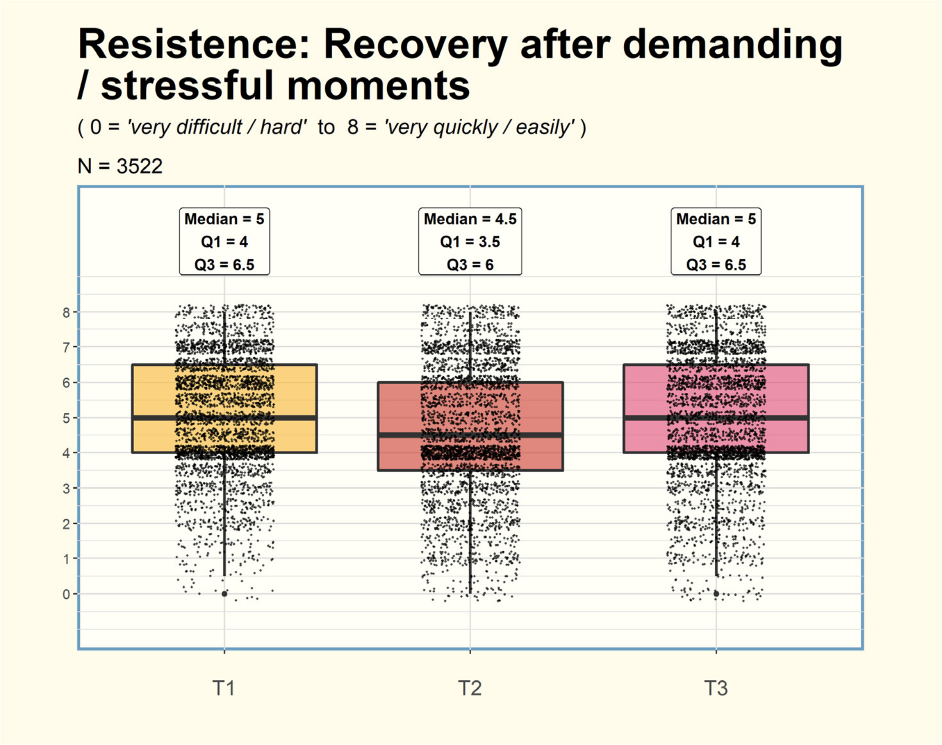

Figure S41. Median levels of resilience or resistance for the three timepoints T1 (Pre-Lockdown), T2 (Lockdown), and T3 (Re-opening). Error bars indicate interquartile range. Q1 = Lower Quartile, Q2 = Upper Quartile.

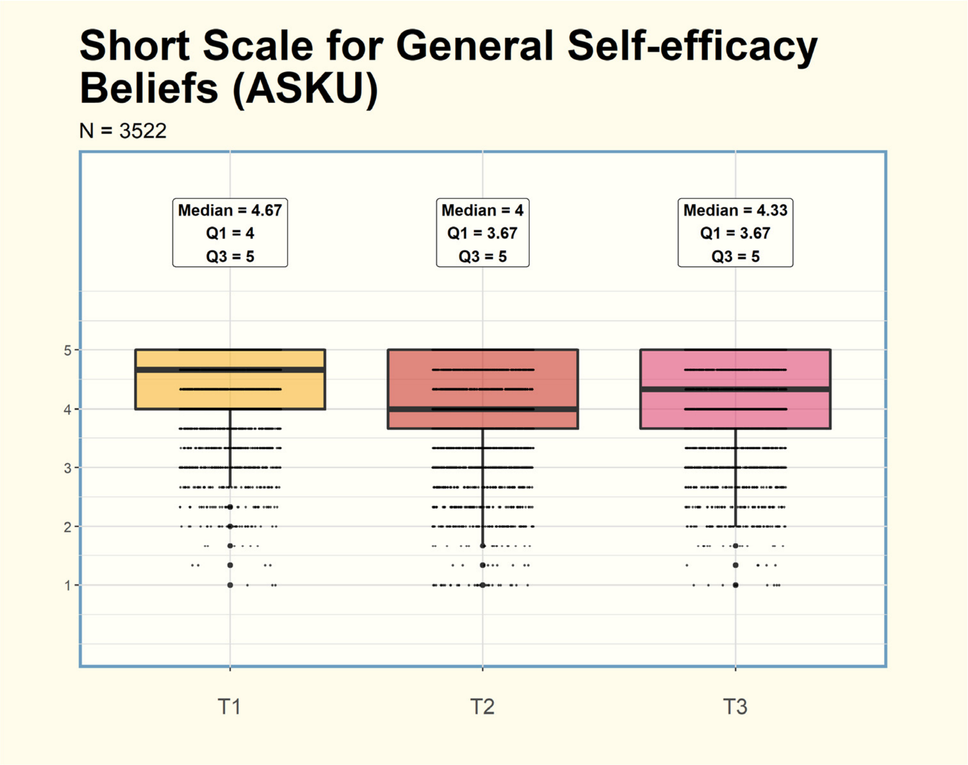

Figure S42. Median levels of self-efficacy for the three timepoints T1 (Pre-Lockdown), T2 (Lockdown), and T3 (Re-opening). Error bars indicate interquartile range. Q1 = Lower Quartile, Q2 = Upper Quartile.

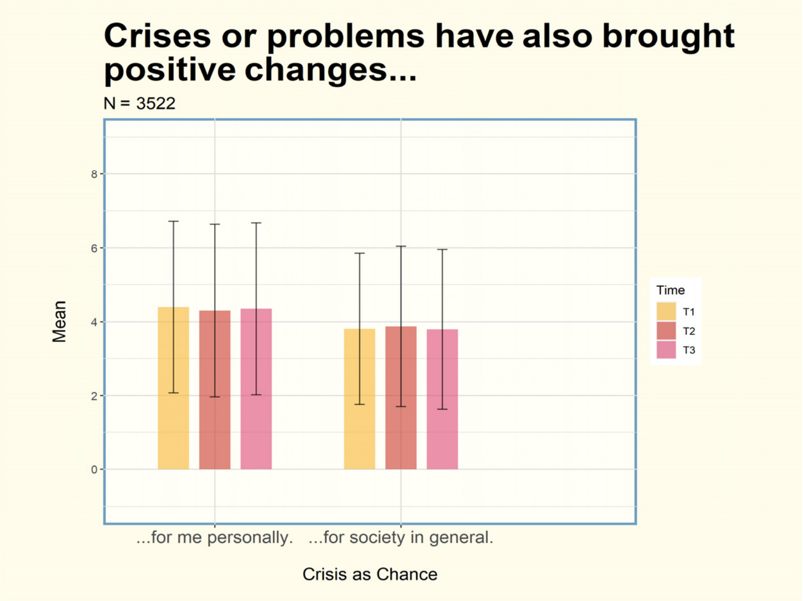

Figure S43. Mean levels of perceiving the crisis as an opportunity for the three timepoints T1 (Pre-Lockdown), T2 (Lockdown), and T3 (Re-opening). Error bars indicate standard deviation.

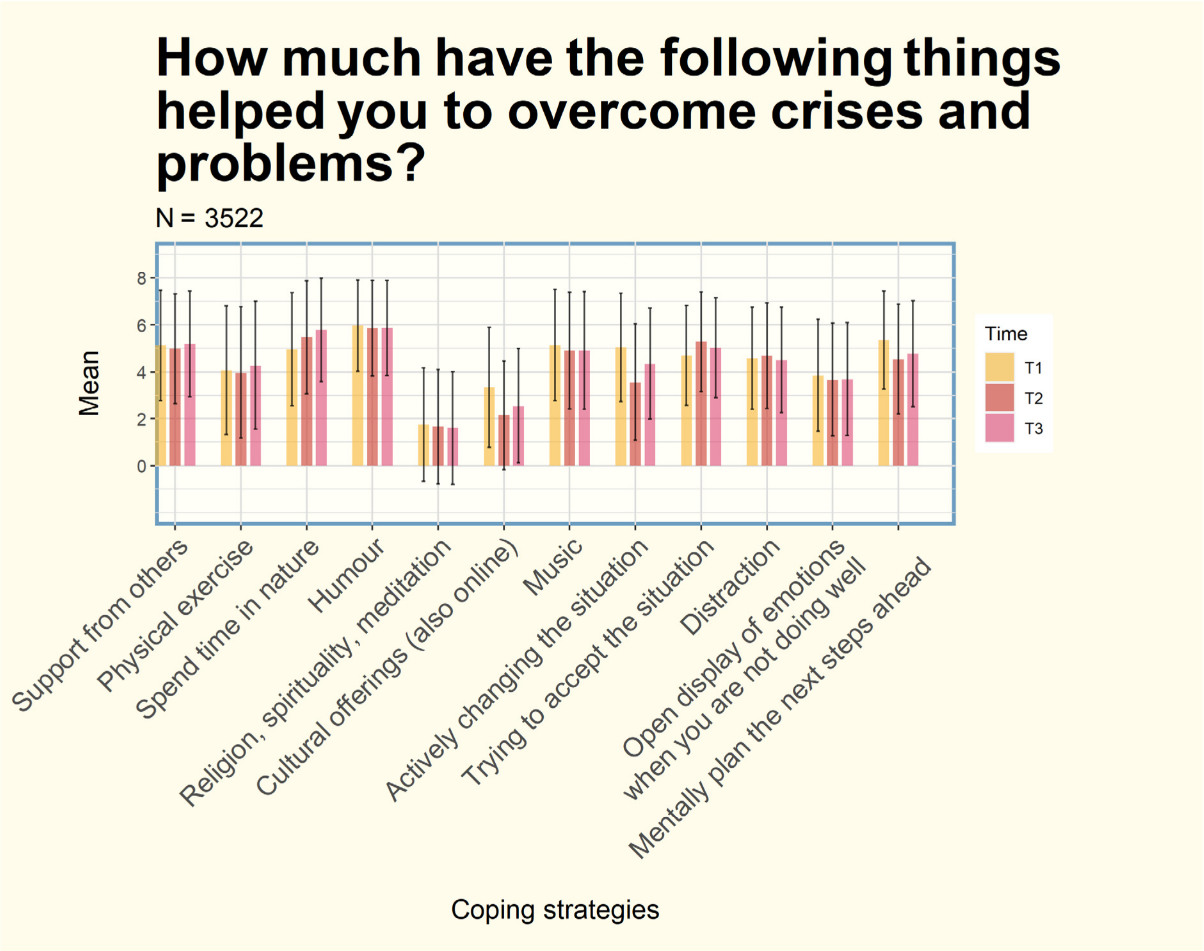

Figure S44. Mean levels of coping strategies used for the three timepoints T1 (Pre-Lockdown), T2 (Lockdown), and T3 (Re-opening). Error bars indicate standard deviation.

6.2.3 State Social Cohesion. Compared to the pre-lockdown period (T1) and reopening period (T3), during T2 we found numerical decreases in mean levels of sense of belonging (Figure S45). With respect to trust, we found numerical decreases in mean levels of trust in friends and fellow citizens during lockdown, but numerical increase in trust in German media, chancellor, and government (Figure S46). During lockdown, participants reported numerical decrease in personal contact (Figure S47), and numerical decreases in positive interactions (Figure S48). Participants reported little to no changes in frequency of online contact during lockdown (Figure S49), with almost no changes in the valence of these interactions (Figure S50). Similarly, during lockdown, we found little to no changes in prosocial behavior towards others (Figure S51) and prosocial behavior experience from others (Figure S52). Lastly, participants reported a decrease in social and political participation during the lockdown period (Figure S53).

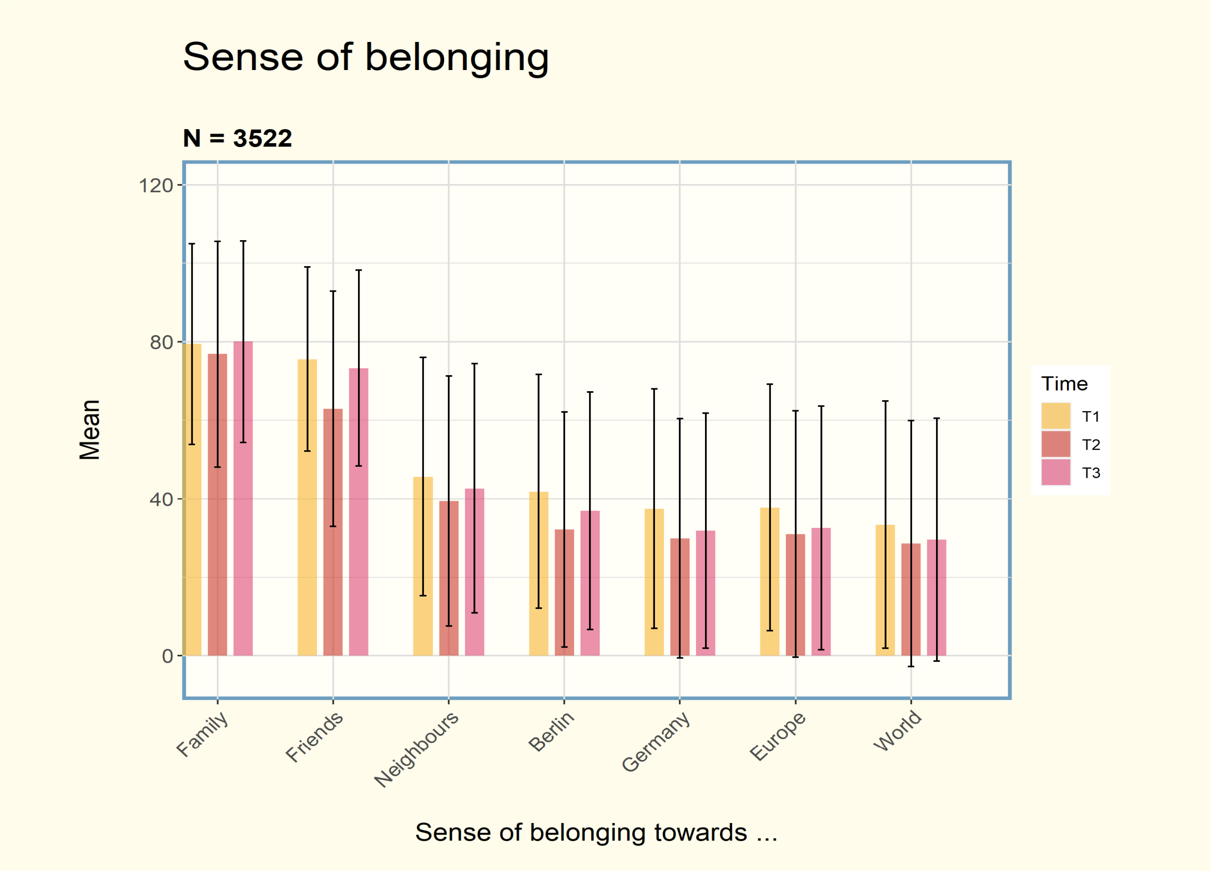

Figure S45. Mean levels of sense of belonging with various people and entities for the three timepoints T1 (Pre-Lockdown), T2 (Lockdown), and T3 (Re-opening). Sense of belonging was measured on a scale from 0 (no sense of belonging) to 100 (highest sense of belonging). Error bars indicate standard deviation.

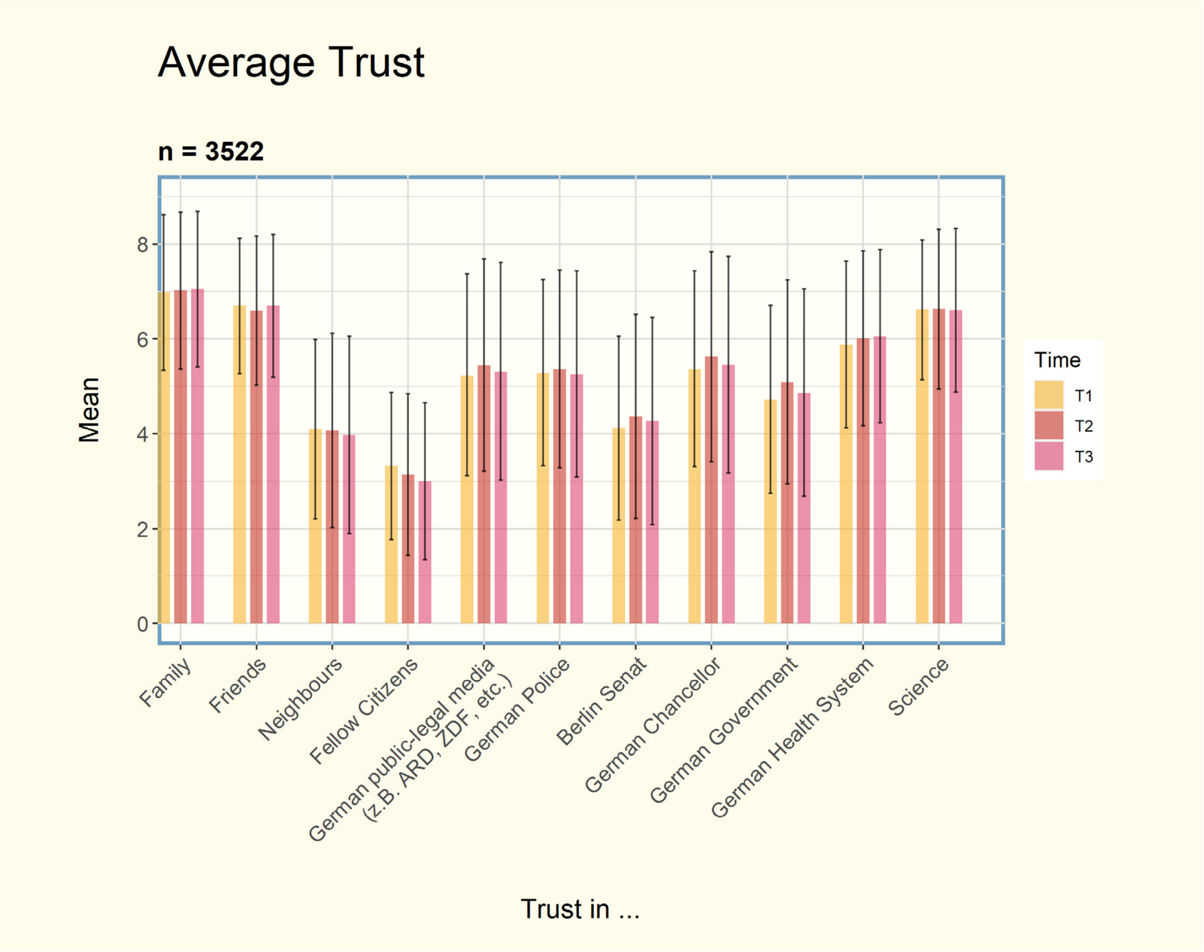

Figure S46. Mean levels of trust in various people and institutions for the three timepoints T1 (Pre-Lockdown), T2 (Lockdown), and T3 (Re-opening). Error bars indicate standard deviation.

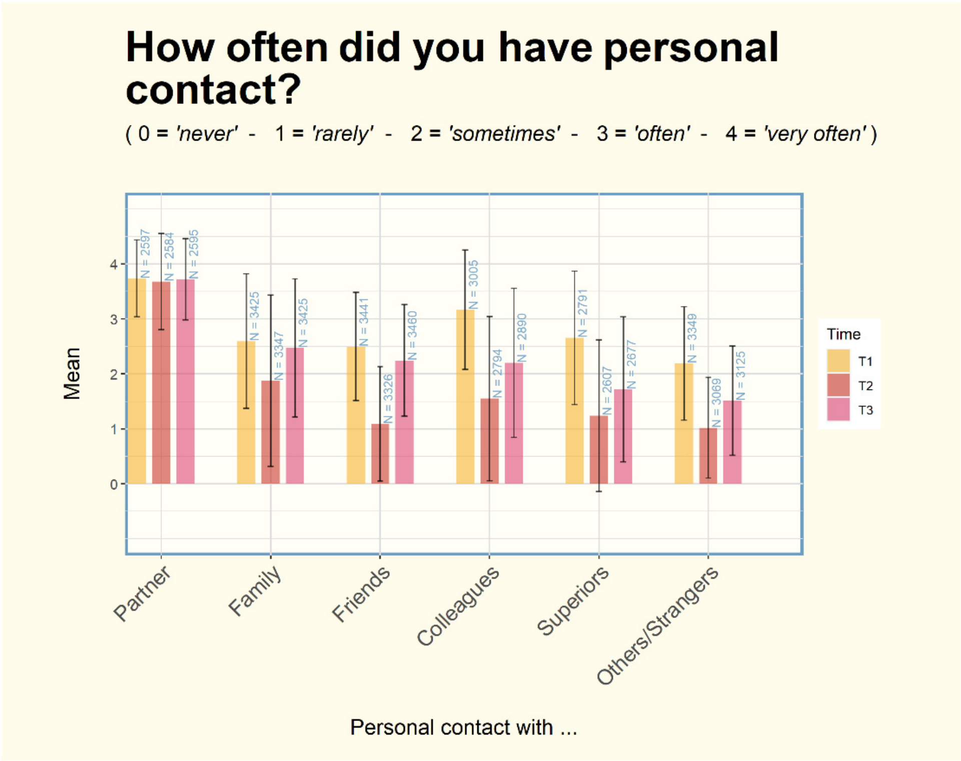

Figure S47. Mean levels of personal contact for the three timepoints T1 (Pre-Lockdown), T2 (Lockdown), and T3 (Re-opening). Error bars indicate standard deviation.

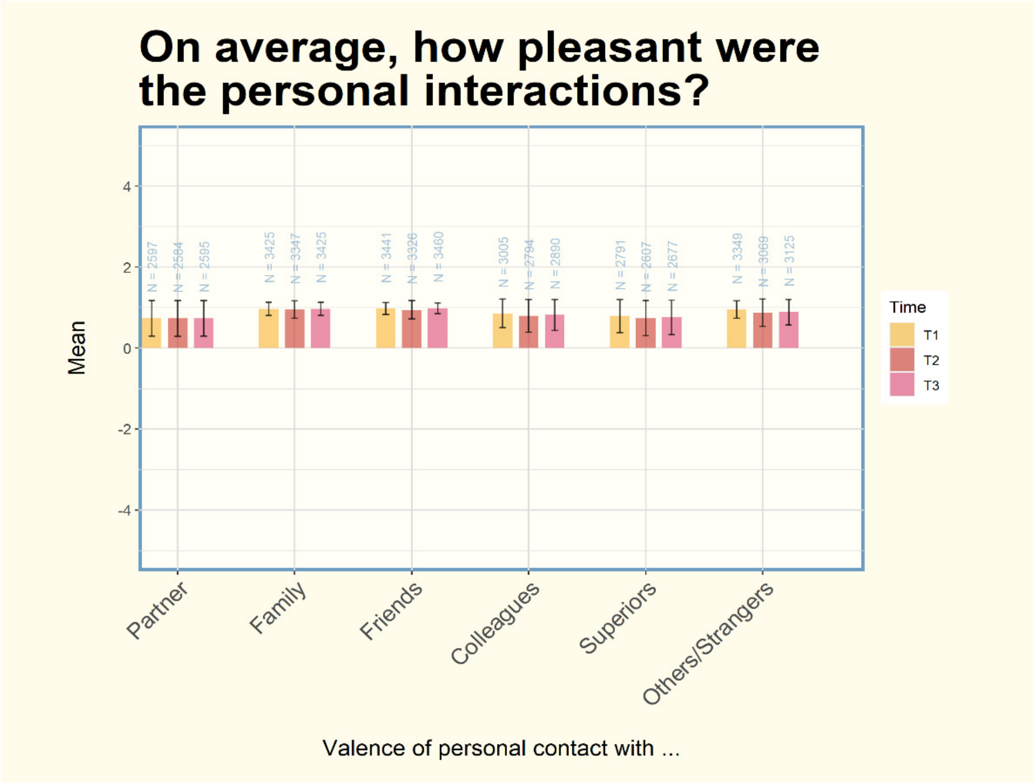

Figure S48. Mean levels of valence of personal contact for the three timepoints T1 (Pre-Lockdown), T2 (Lockdown), and T3 (Re-opening). Error bars indicate standard deviation.

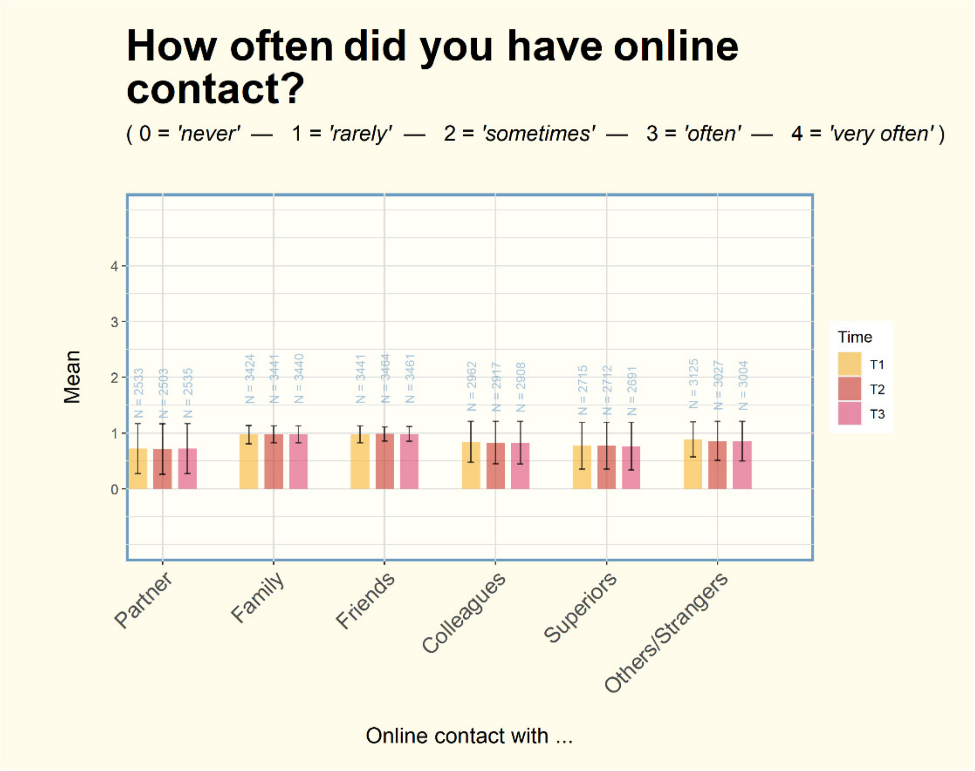

Figure S49. Mean levels of online contact for the three timepoints T1 (Pre-Lockdown), T2 (Lockdown), and T3 (Re-opening). Error bars indicate standard deviation.

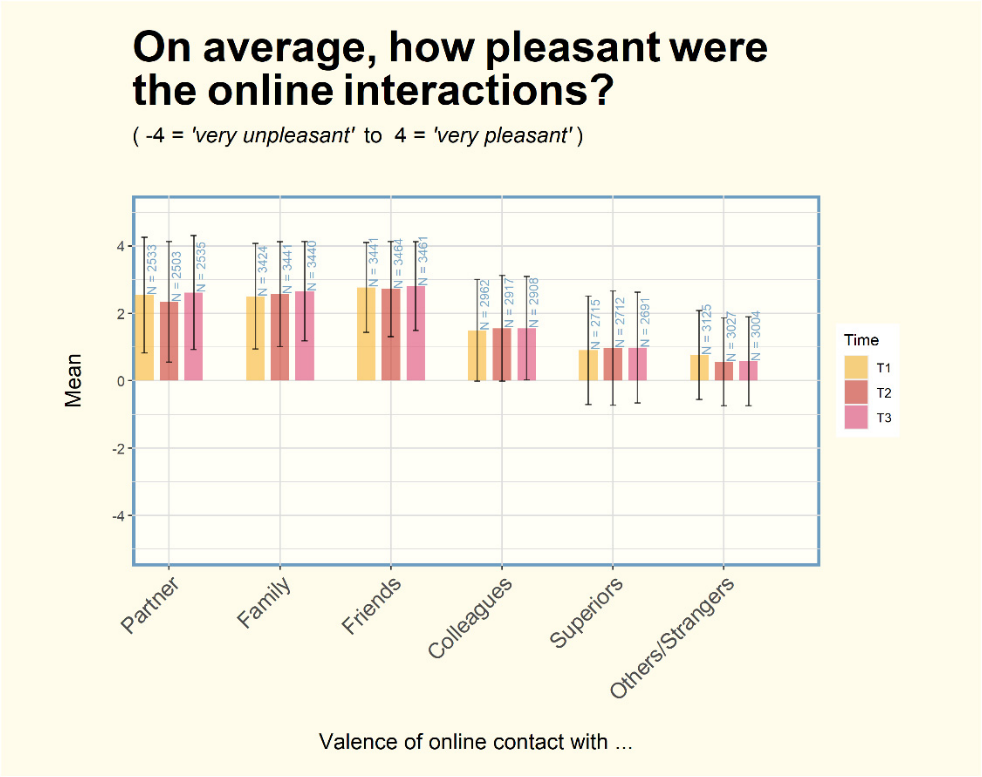

Figure S50. Mean levels of valence of online contact for the three timepoints T1 (Pre-Lockdown), T2 (Lockdown), and T3 (Re-opening). Error bars indicate standard deviation.

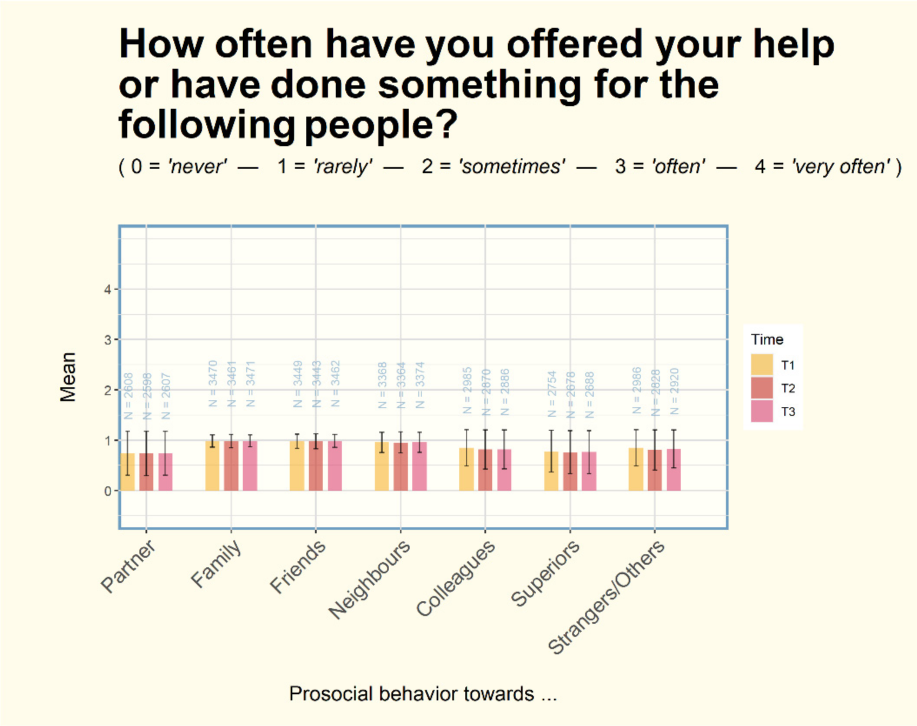

Figure S51. Mean levels of prosocial behaviors towards others for the three timepoints T1 (Pre-Lockdown), T2 (Lockdown), and T3 (Re-opening). Error bars indicate standard deviation.

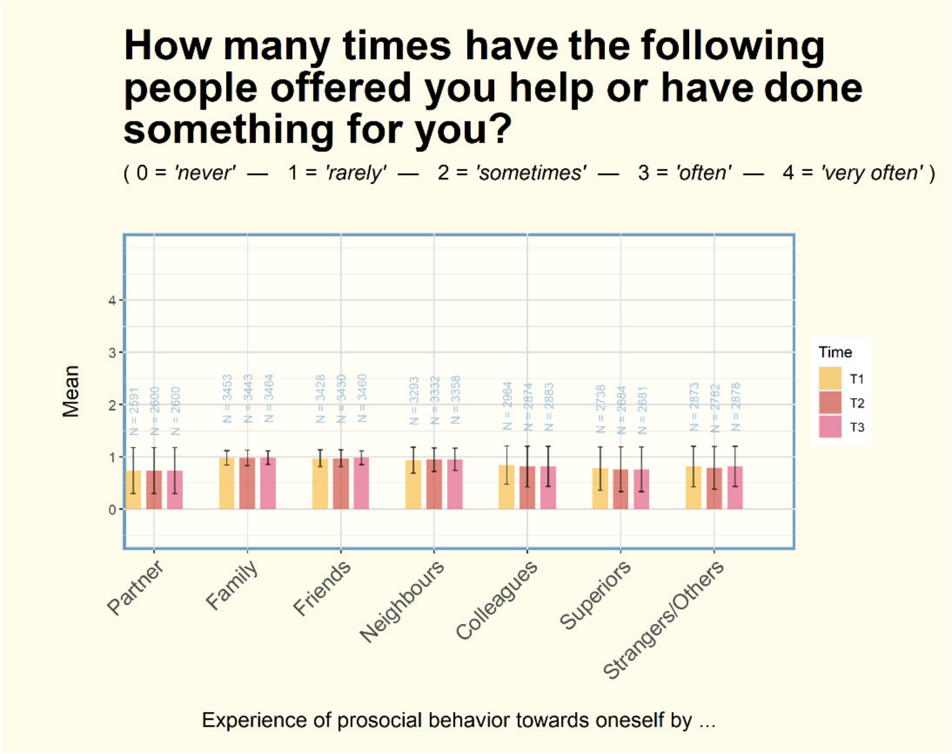

Figure S52. Mean levels of prosocial behavior experienced from others for the three timepoints T1 (Pre-Lockdown), T2 (Lockdown), and T3 (Re-opening). Error bars indicate standard deviation.

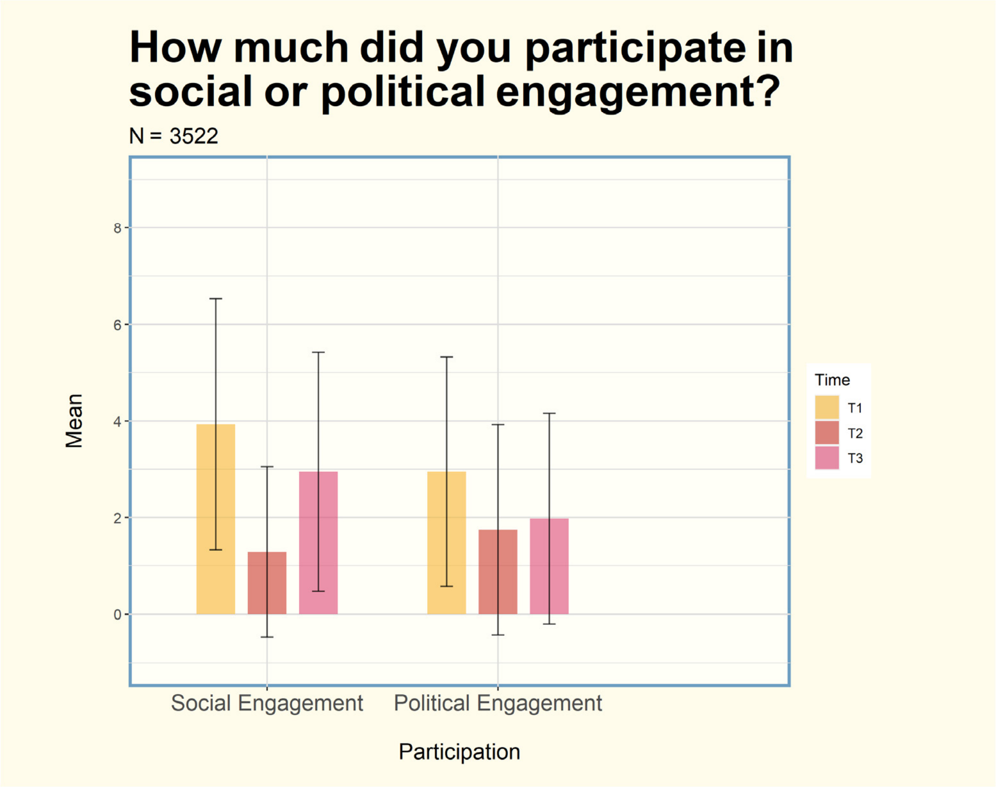

Figure S53. Mean levels of social and political participation for the three timepoints T1 (Pre-Lockdown), T2 (Lockdown), and T3 (Re-opening).

5.3 COVID-specific variables

Compared to pre-lockdown and lockdown timepoints, participants reported spending more time outdoors during reopening period (Figure S54). However, across the three timepoints, participants reported little to no numerical changes in perceived financial security, financial ability to cover basic needs, workload, number of working hours, number and type of co-residents (Figures S55–S60). However, participants reported greater numerical covid-related anxiety during the lockdown compared to T1 and T3 (Figure S61). Lastly, participants also reported numerical increases in all covid-specific fears (Figures S62

and S63) and covid-specific fear behaviors (Figure S64) during lockdown, compared to T1 and T3.

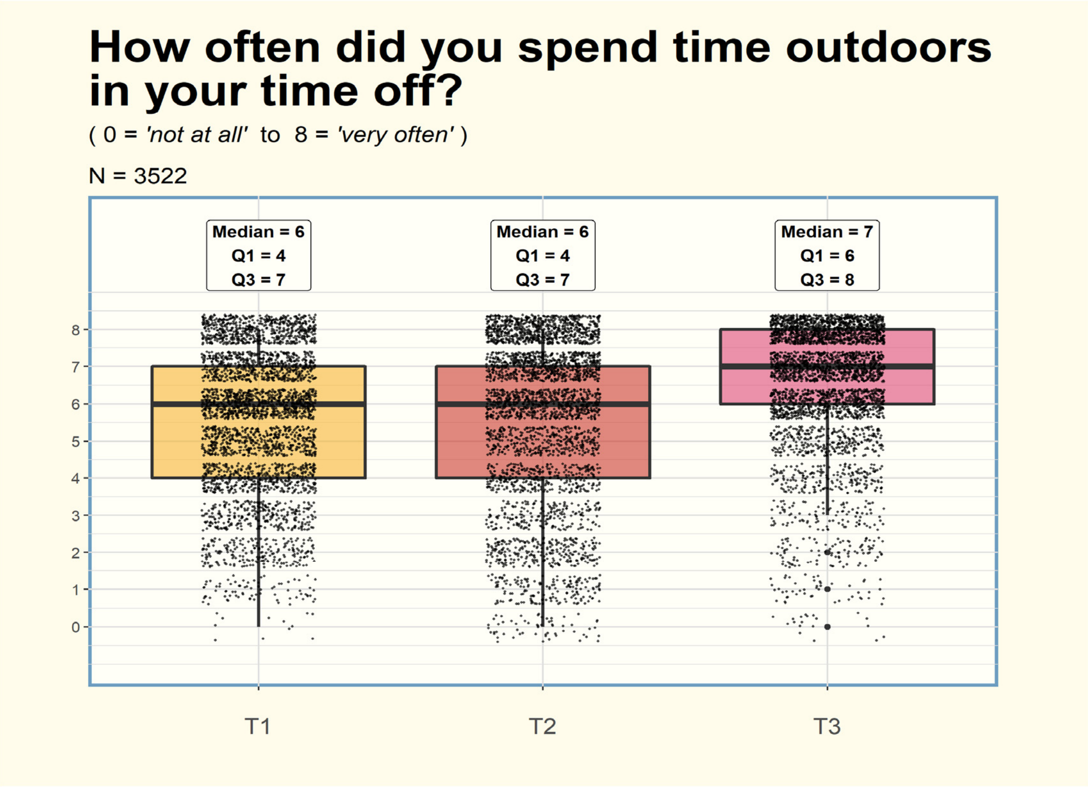

Figure S54. Median levels of time spent outdoors for the three timepoints T1 (Pre-Lockdown), T2 (Lockdown), and T3 (Re-opening). Error bars indicate interquartile range. Q1 = Lower Quartile, Q2 = Upper Quartile.

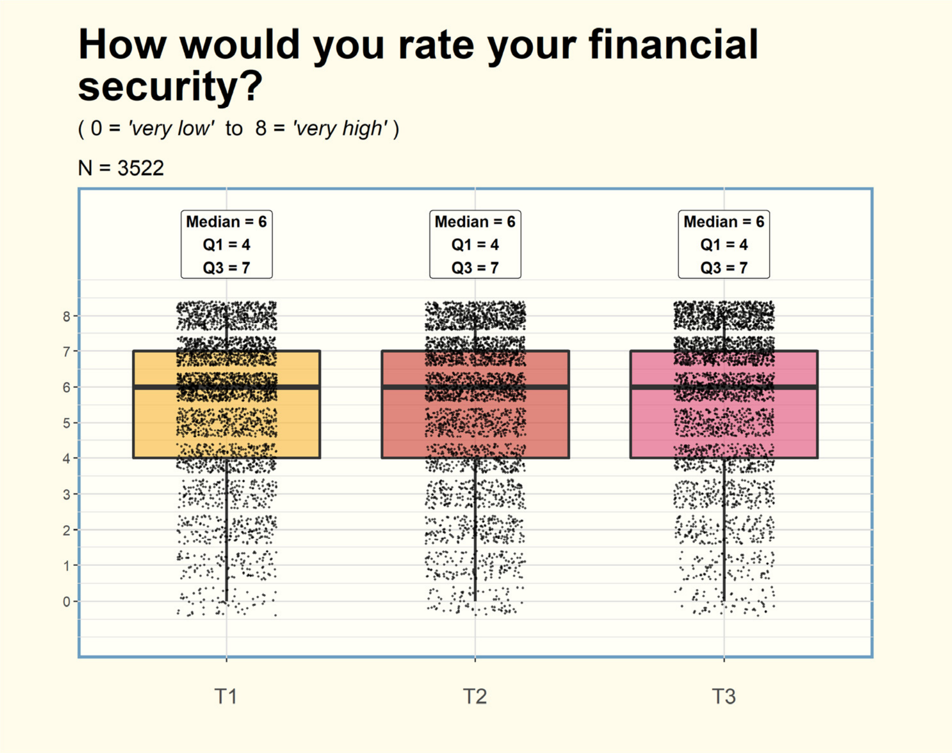

Figure S55. Median levels of financial security for the three timepoints T1 (Pre-Lockdown), T2 (Lockdown), and T3 (Re-opening). Error bars indicate interquartile range. Q1 = Lower Quartile, Q2 = Upper Quartile.

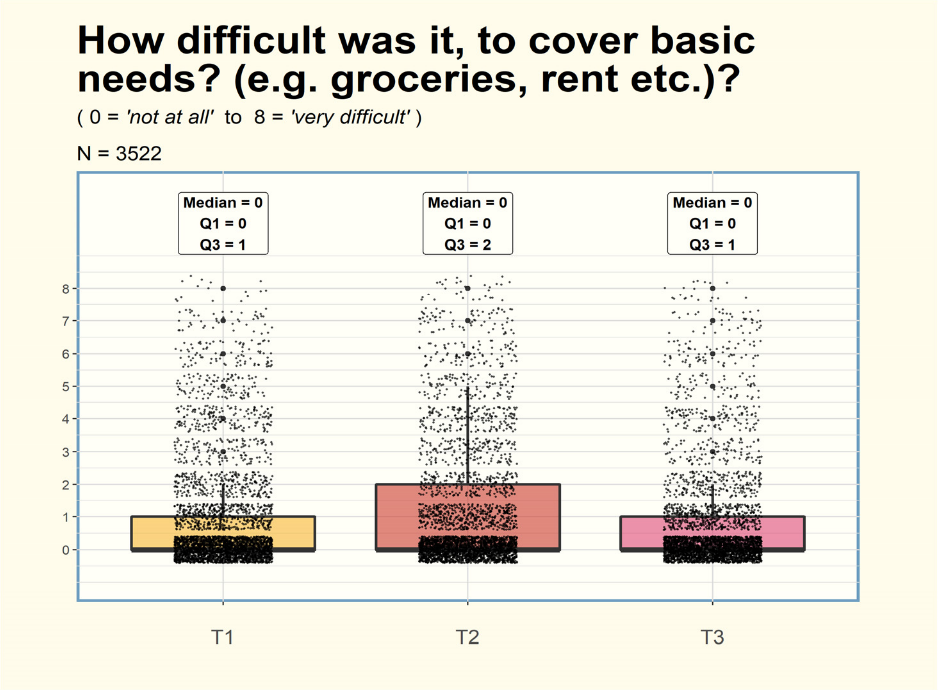

Figure S56. Median levels of ability to cover basic needs for the three timepoints T1 (Pre-Lockdown), T2 (Lockdown), and T3 (Re-opening). Error bars indicate interquartile range. Q1 = Lower Quartile, Q2 = Upper Quartile.

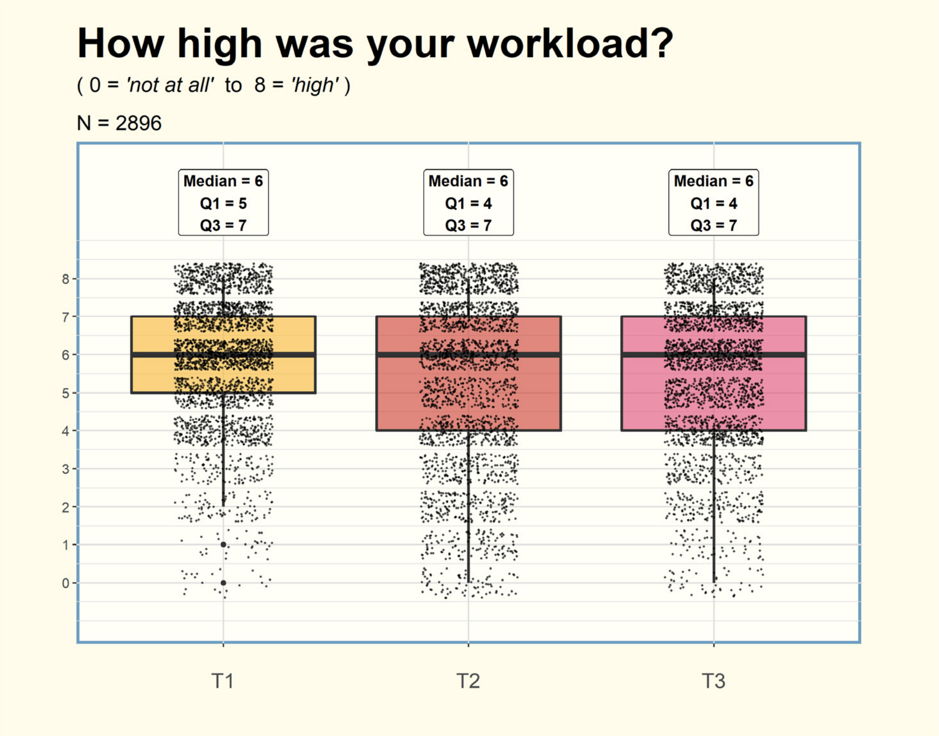

Figure S57. Median levels of perceived workload for the three timepoints T1 (Pre-Lockdown), T2 (Lockdown), and T3 (Re-opening). These numbers are reported only for individuals who indicated that they were engaged in some form of employment. Error bars indicate interquartile range. Q1 = Lower Quartile, Q2 = Upper Quartile.

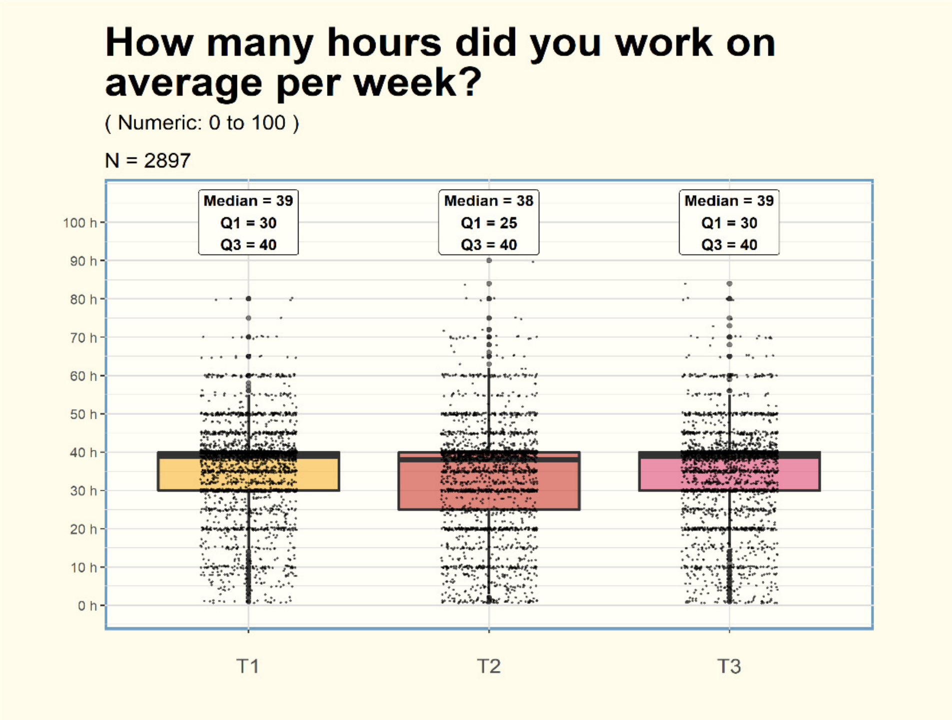

Figure S58. Median levels of weekly working hours for the three timepoints T1 (Pre-Lockdown), T2 (Lockdown), and T3 (Re-opening). These numbers are reported only for individuals who indicated that they were engaged in some form of employment. Error bars indicate interquartile range. Q1 = Lower Quartile, Q2 = Upper Quartile.

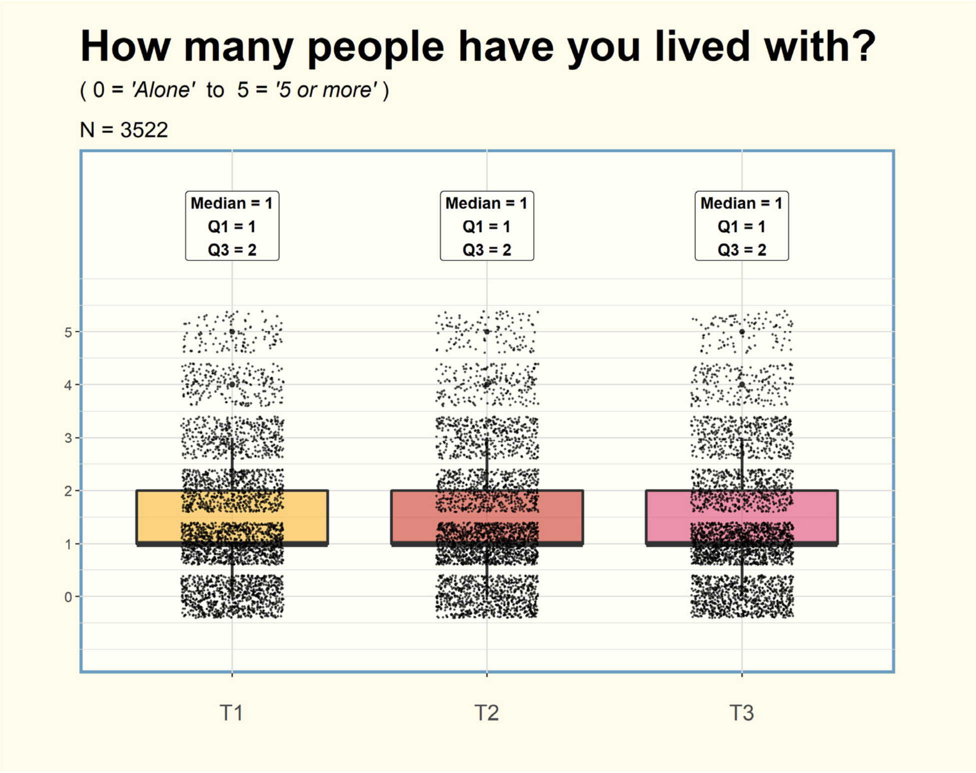

Figure S59. Median levels of weekly working hours for the three timepoints T1 (Pre-Lockdown), T2 (Lockdown), and T3 (Re-opening). Error bars indicate interquartile range. Q1 = Lower Quartile, Q2 = Upper Quartile.

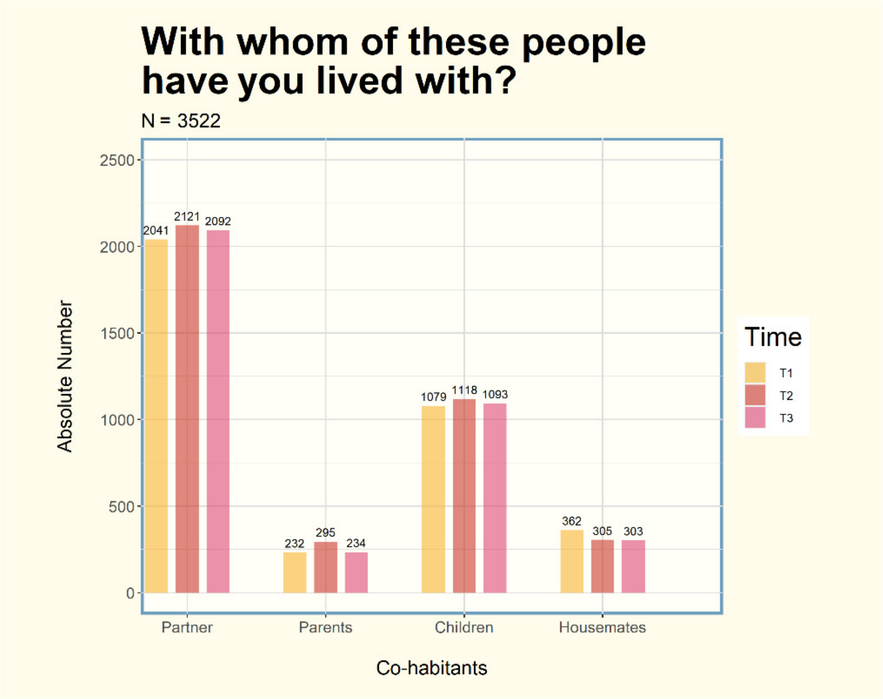

Figure S60. Absolute numbers of participants in our sample living with various type of co-residents for the three timepoints T1 (Pre-Lockdown), T2 (Lockdown), and T3 (Re-opening).

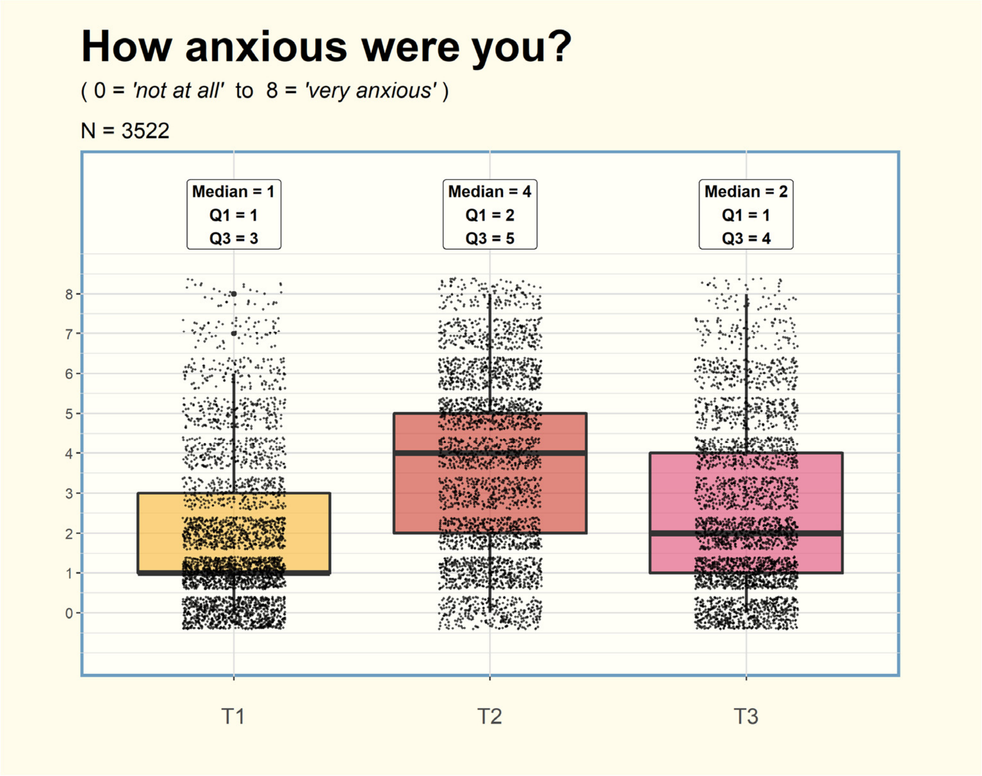

Figure S61. Median levels of covid-specific anxiety or fear for the three timepoints T1 (Pre-Lockdown), T2 (Lockdown), and T3 (Re-opening). Error bars indicate interquartile range. Q1 = Lower Quartile, Q2 = Upper Quartile.

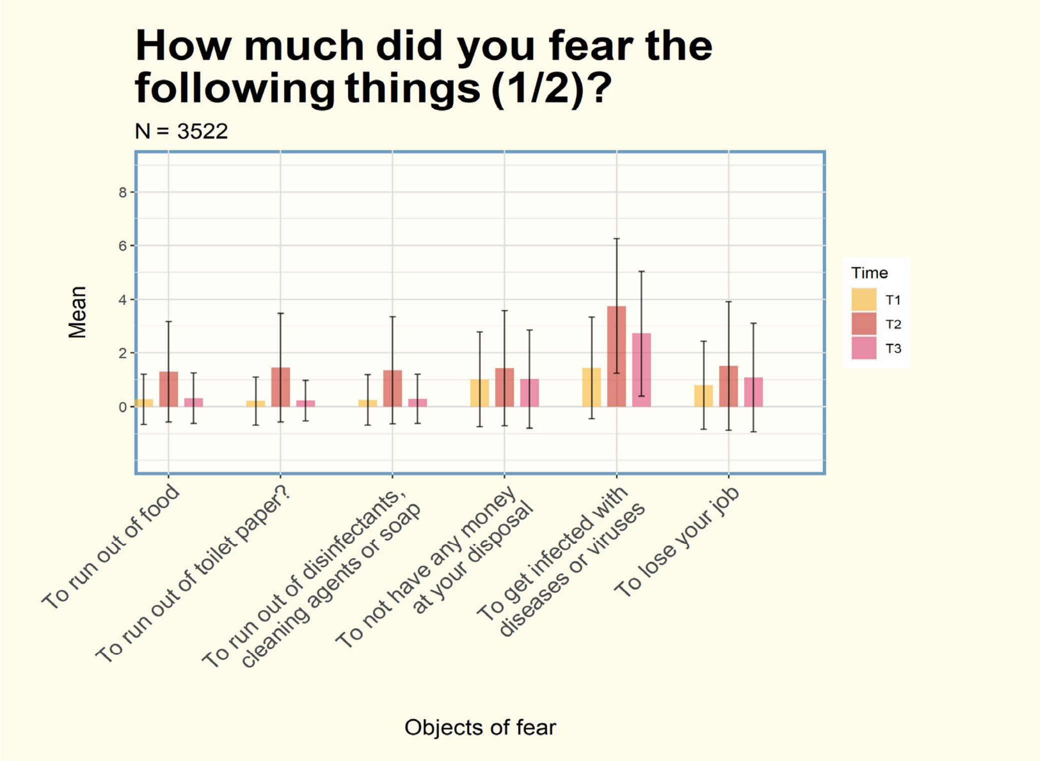

Figure S62. Mean levels of covid-specific fears for the three timepoints T1 (Pre-Lockdown), T2 (Lockdown), and T3 (Re-opening). Error bars indicate standard deviation.

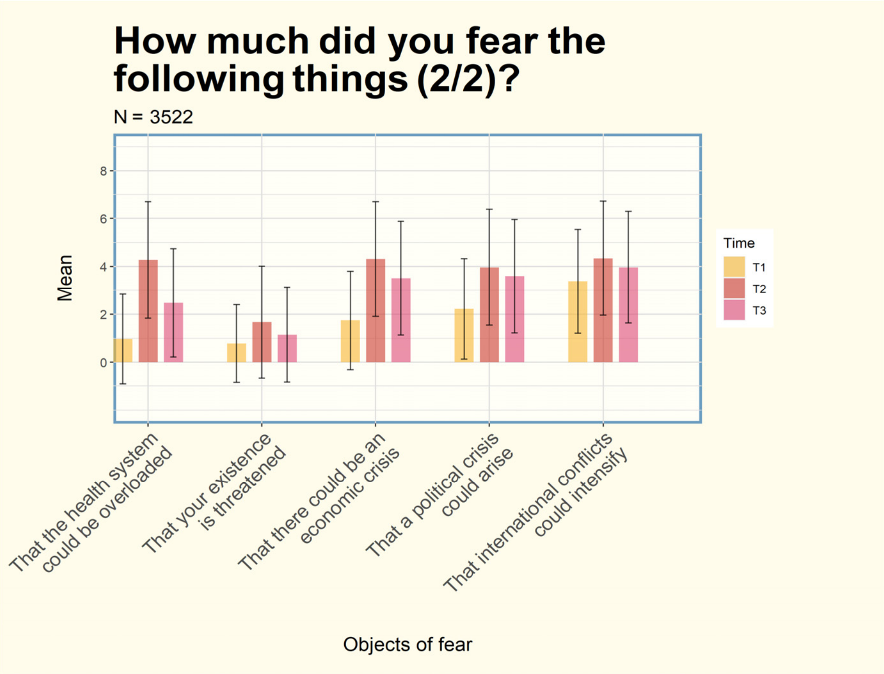

Figure S63. Mean levels of covid-specific fears for the three timepoints T1 (Pre-Lockdown), T2 (Lockdown), and T3 (Re-opening). Error bars indicate standard deviation.

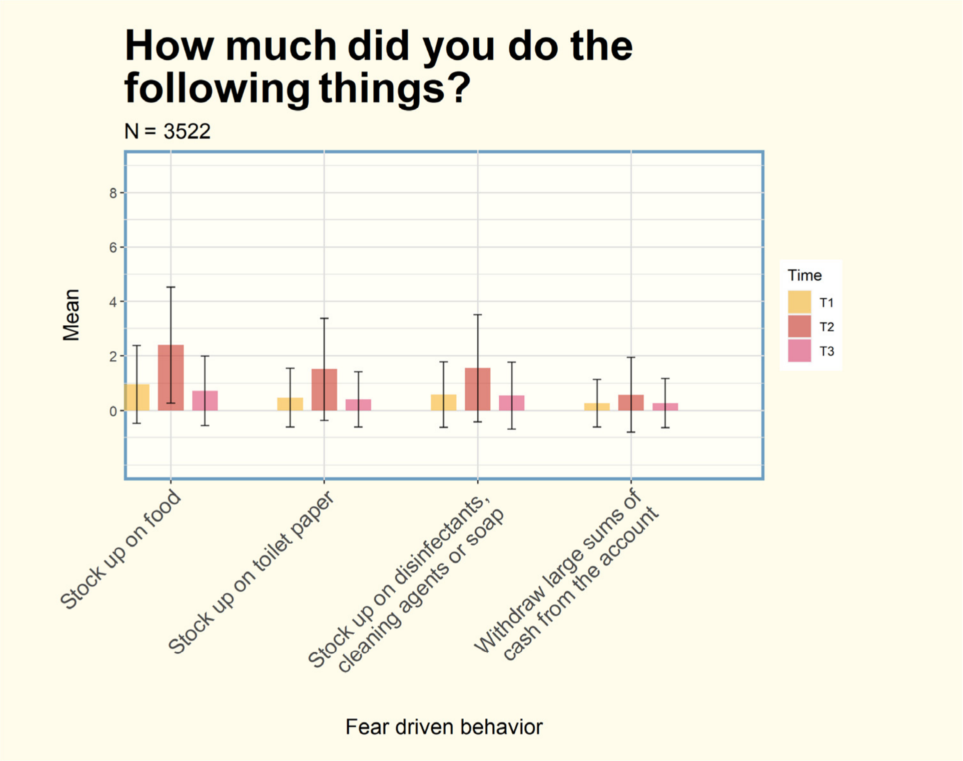

Figure S64. Mean levels of covid-specific fear behaviors for the three timepoints T1 (Pre-Lockdown), T2 (Lockdown), and T3 (Re-opening). Error bars indicate standard deviation.
