# Supplementary material for: Coping with the COVID-19 Pandemic: Perceived Changes in Psychological Vulnerability, Resilience and Social Cohesion before, during and after Lockdown
Source: Int J Environ Res Public Health. 2022 Mar 10;19(6):3290. doi: 10.3390/ijerph19063290 (PMC8952288; doi:10.3390/ijerph19063290)
Supplement: Supplementary file 1 [file ijerph-19-03290-s001.zip › Supplement S2.pdf]

Supplement S2. Item statistics

Table S1. Item and composite statistics for vulnerability and resilience indicators

		T1			T2			T3		
	scale range	*M*	*SD*	*α*	*M*	*SD*	*α*	*M*	*SD*	*α*
Vulnerability indicators										
Burdens	0 - 8	1.38	.93	.86 [.85, .86]	2.26	1.07	.84 [.83, .85]	1.82	1.01	.85 [.85, .86]
Appropriateness of protection measures				.38 [.35, .41]			.18 [.14, .23]			.17 [.12, .26]
	0 - 8	1.79	2.13		2.10	2.42		1.97	2.23	
	0 - 8	2.86	2.44		2.56	2.44		3.10	2.51	
	0 - 8	2.54	2.54		3.02	2.70		2.66	2.47	
	0 - 8	2.25	2.17		2.28	2.11		2.81	2.24	
Psychosomatic complaints	0 - 8	1.77	1.26	.75 [.74, .76]	1.93	1.44	.79 [.78, .80]	1.52	1.29	.79 [.78, .80]
Aggression as perpetrator	0 - 8	.60	.74	.80 [.79, .81]	.61	.78	.77 [.76, .78]	.48	.72	.81 [.80, .82]
Aggression as victim	0 - 8	.55	.64	.70 [.68, .71]	.55	.70	.70 [.69, .72]	.48	.65	.72 [.70, .73]
Craving for internet activities	0 - 8	1.93	1.09	.60 [.58, .62]	2.27	1.21	.59 [.57, .61]	1.90	1.14	.61 [.59, .63]
Pandemic-related fears	0 - 8	1.19	1.05	.84 [.84, .85]	2.67	1.40	.84 [.83, .85]	1.85	1.15	.82 [.81, .83]
Pandemic-related behaviors	0 - 8	.57	.92	.81 [.80, .82]	1.51	1.48	.81 [.80, .82]	.49	.87	.80 [.79, .81]
Alcohol consumption (AUDIT-C)	0 - 12	2.65	2.04	.64 [.62, .66]	2.52	2.28	.66 [.65, .68]	2.58	2.21	.68 [.66, .69]
Internet Use (CIUS-5)	0 - 20	4.52	3.78	.78 [.77, .80]	4.90	4.31	.82 [.81, .82]	3.79	3.84	.82 [.81, .83]
Perceived stress (PSS-4)	0 - 16	5.86	2.78	.76 [.75, .77]	7.09	3.06	.79 [.78, .80]	6.05	3.04	.80 [.79, .81]
Depressive symptoms (PHQ-2)	0 - 6	1.34	1.23	.73 [.71, .74]	1.89	1.51	.80 [.78, .81]	1.47	1.37	.78 [.77, .80]
Anxiety symptoms (GAD-2)	0 - 6	1.25	1.32	.75 [.73, .77]	1.83	1.61	.80 [.79, .81]	1.42	1.45	.80 [.79, .81]
News consumption*	0 - 8	5.04	2.11		6.75	1.61		5.59	2.05	
Alcohol loss of control*	0 - 8	.43	1.13		.75	1.56		.61	1.37	
Overall stress*	0 - 8	4.00	2.07		4.59	2.30		3.91	2.14	
Loneliness*	0 - 8	1.85	2.08		3.24	2.52		2.44	2.20	
Overall anxiety*	0 - 8	1.94	1.90		3.57	2.30		2.49	2.08	
Resilience indicators										
Stress recovery	0 - 8	5.03	1.65	.75 [.73, .77]	4.60	1.88	.82 [.81, .83]	4.92	1.83	.81 [.80, .82]
Crises as a chance	0 - 8	4.10	1.95	.74 [.72, .76]	4.09	2.04	.78 [.77, .79]	4.07	2.05	.81 [.79, .82]
Coping during crises	0 - 8	4.49	1.18	.74 [.73, .75]	4.22	1.16	.71 [.70, .73]	4.38	1.17	.73 [.72, .74]
Self-efficacy (ASKU)	1 - 5	4.37	.72	.89 [.88, .89]	4.04	.89	.90 [.90, .91]	4.18	.83	.91 [.91, .92]
Optimism*	0 - 8	5.69	1.72		4.33	1.92		5.03	1.82	
Life satisfaction*	0 - 8	5.77	1.64		4.66	1.94		5.41	1.71	

Note. Sample mean (*M*), standard deviation (*SD*), Cronbach's Alpha with 95% confidence interval (*α*) for indicators at three measurement occasions of January 2020 (T1), mid of March to mid of April 2020 (T2) and June 2020 (T3). There was no missing data. * = single-item indicator.

Table S2. Item statistics for social cohesion indicators

Social cohesion indicators	scale range	T1 *M*	T1 *SD*	T1 NA	T2 *M*	T2 *SD*	T2 NA	T3 *M*	T3 *SD*	T3 NA
Belonging family	1-100	6.36	2.05	-	6.15	2.30	-	6.40	2.06	-
Belonging friends	1-100	6.04	1.87	-	5.03	2.40	-	5.86	1.99	-
Belonging neighborhood	1-100	3.65	2.43	-	3.15	2.55	-	3.41	2.54	-
Belonging Berlin	1-100	3.35	2.38	-	2.57	2.40	-	2.95	2.42	-
Belonging Germany	1-100	2.99	2.44	-	2.39	2.44	-	2.55	2.40	-
Belonging Europe	1-100	3.02	2.51	-	2.47	2.51	-	2.60	2.48	-
Belonging World	1-100	2.67	2.52	-	2.28	2.51	-	2.37	2.48	-
Trust family	0 - 8	6.98	1.64	-	7.02	1.65	-	7.05	1.64	-
Trust friends	0 - 8	6.70	1.43	-	6.60	1.57	-	6.70	1.50	-
Trust neighbors	0 - 8	4.10	1.89	-	4.07	2.05	-	3.97	2.08	-
Trust fellow citizens	0 - 8	3.32	1.55	-	3.14	1.71	-	3.00	1.66	-
Trust media	0 - 8	5.24	2.12	-	5.45	2.24	-	5.32	2.29	-
Trust police	0 - 8	5.29	1.96	-	5.37	2.08	-	5.26	2.17	-
Trust senate	0 - 8	4.12	1.93	-	4.37	2.15	-	4.27	2.18	-
Trust chancellor	0 - 8	5.37	2.06	-	5.63	2.21	-	5.46	2.28	-
Trust government	0 - 8	4.72	1.97	-	5.09	2.15	-	4.87	2.18	-
Trust health system	0 - 8	5.88	1.76	-	6.01	1.84	-	6.06	1.82	-
Trust science	0 - 8	6.62	1.47	-	6.63	1.68	-	6.61	1.72	-
Interaction frequency partner	0 - 8	3.74	.70	925	3.68	.88	938	3.72	.74	927
Interaction quality partner	-4 - 4	2.33	1.89	925	2.20	1.94	938	2.37	1.89	927
Interaction frequency family	0 - 8	2.60	1.22	97	1.87	1.56	175	2.47	1.26	97
Interaction quality family	-4 - 4	2.62	1.57	97	2.00	1.81	175	2.69	1.54	97
Interaction frequency friends	0 - 8	2.50	.98	81	1.09	1.04	196	2.24	1.01	62
Interaction quality friends	-4 - 4	2.97	1.26	81	1.85	1.77	196	2.88	1.35	62
Interaction frequency colleagues	0 - 8	3.17	1.09	517	1.55	1.49	728	2.20	1.35	632
Interaction quality colleagues	-4 - 4	1.57	1.59	517	.84	1.58	728	1.29	1.64	632
Interaction frequency supervisors	0 - 8	2.65	1.21	731	1.24	1.38	915	1.72	1.32	845
Interaction quality supervisors	-4 - 4	.95	1.81	731	.43	1.51	915	.70	1.67	845
Interaction frequency others	0 - 8	2.19	1.03	173	1.02	.91	453	1.51	1.00	397
Interaction quality others	-4 - 4	.83	1.45	173	.11	1.43	453	.39	1.46	397
Prosocial frequency self -partner	0 - 4	3.25	.84	914	3.21	.89	924	3.17	.90	915
Prosocial frequency partner - self	0 - 4	3.06	1.01	931	3.08	1.01	922	3.05	1.02	922
Prosocial frequency self -family	0 - 4	2.73	1.06	52	2.67	1.13	61	2.60	1.10	51
Prosocial frequency family - self	0 - 4	2.34	1.12	69	2.27	1.19	79	2.34	1.14	58
Prosocial frequency self -friends	0 - 4	2.37	1.02	73	2.08	1.12	79	2.15	1.05	60
Prosocial frequency friends - self	0 - 4	2.11	1.04	94	1.89	1.14	92	2.05	1.07	62
Prosocial frequency self -neighbors	0 - 4	1.19	1.03	154	1.17	1.09	158	1.03	1.02	148
Prosocial frequency neighbors - self	0 - 4	.96	.98	229	.91	1.03	190	.86	.98	164
Prosocial frequency self -colleagues	0 - 4	1.99	1.07	537	1.65	1.15	652	1.66	1.11	636
Prosocial frequency colleagues - self	0 - 4	1.60	1.01	558	1.34	1.06	648	1.38	1.04	639
Prosocial frequency self - supervisors	0 - 4	1.49	1.19	768	1.20	1.16	844	1.20	1.14	834
Prosocial frequency supervisors - self	0 - 4	1.17	1.04	784	1.04	1.05	838	1.02	1.01	841
Prosocial frequency self -others	0 - 4	1.04	.93	536	.75	.91	694	.75	.86	602
Prosocial frequency others - self	0 - 4	.67	.78	649	.46	.72	740	.52	.73	644
Prosocial efforts	0 - 8	3.19	1.70	-	3.13	1.83	-	3.01	1.78	-
Political participation	0 - 8	2.95	2.37	-	1.75	2.18	-	1.98	2.18	-
Social participation	0 - 8	3.93	2.60	-	1.29	1.76	-	2.94	2.47	-

Note. Sample mean (*M*), standard deviation (*SD*), and not available data cases (NA) for indicators at three measurement occasions of January 2020 (T1), mid of March to mid of April 2020 (T2) and June 2020 (T3).
