# Supplementary material for: Coping with the COVID-19 Pandemic: Perceived Changes in Psychological Vulnerability, Resilience and Social Cohesion before, during and after Lockdown
Source: Int J Environ Res Public Health. 2022 Mar 10;19(6):3290. doi: 10.3390/ijerph19063290 (PMC8952288; doi:10.3390/ijerph19063290)
Supplement: Supplementary file 1 [file ijerph-19-03290-s001.zip › Supplement S3.pdf]

Supplement S3. Exploratory factor analyses

Table S3. Factor loadings of vulnerability indicators at T1

	Factor 1	Factor 2	Factor 3	Factor 4	Factor 5
Anxiety symptoms (GAD-2)	**.89**	-.04	-.07	-.06	.01
Depressive symptoms (PHQ-2)	**.79**	-.02	-.13	.06	.03
Overall Anxiety	**.72**	-.04	.07	-.03	-.02
Perceived stress (PSS-4)	**.68**	.01	.02	.07	.01
Loneliness	**.63**	.01	-.09	.09	.04
Psychosomatic complaints	**.60**	.06	.11	.00	-.05
Overall stress	**.59**	.00	-.03	-.10	.00
Burdens	**.52**	.06	**.35**	.00	-.01
Aggression as victim	.02	**.93**	-.12	.03	-.02
Aggression as perpetrator	-.01	**.69**	-.02	.00	.05
Pandemic-related fears	.19	-.12	**.83**	-.06	.02
Pandemic-related behaviours	-.03	-.01	**.49**	-.03	-.04
Craving for internet activities	-.09	.01	.04	**.79**	-.01
Internet Use (CIUS-5)	.09	.01	-.02	**.65**	.00
Alcohol consumption (AUDIT-C)	-.09	.03	-.03	-.01	**.77**
Alcohol loss of control	.09	.02	.05	.00	**.63**
News consumption	-.03	-.05	.20	.02	.03
Protection measures item 1	-.03	.08	.13	-.02	.00
Protection measures item 4	.02	.04	.18	.04	-.02
Eigenvalues	3.91	1.26	1.15	1.13	1.05
Variance proportion	.21	.07	.06	.06	.06

Note. Factor loadings above .30 in bold

Table S4. Factor loadings of vulnerability indicators at T2

	Factor 1	Factor 2	Factor 3	Factor 4	Factor 5
Anxiety symptoms (GAD-2)	**.88**	-.08	-.17	.15	-.02
Depressive symptoms (PHQ-2)	**.85**	-.04	.05	-.02	-.03
Overall Anxiety	**.82**	-.04	-.03	.06	-.05
Perceived stress (PSS-4)	**.63**	-.08	-.02	.13	-.01
Loneliness	**.62**	.12	.14	-.07	-.03
Psychosomatic complaints	**.60**	-.09	**.41**	-.07	-.05
Overall stress	**.58**	.05	.22	-.20	-.01
Burdens	**.54**	.16	**.31**	-.01	.00
Aggression as victim	-.02	**.87**	-.07	-.06	.05
Aggression as perpetrator	.01	**.82**	-.06	-.07	.06
Pandemic-related fears	.21	-.01	**.69**	.04	.01
Pandemic-related behaviours	-.03	.02	**.54**	.06	.02
Craving for internet activities	-.03	-.07	.10	**.71**	.07
Internet Use (CIUS-5)	.19	-.04	-.02	**.68**	.01
Alcohol consumption (AUDIT-C)	-.14	.04	.09	.02	**.86**
Alcohol loss of control	.04	.05	.18	.06	**.77**
News consumption	-.02	-.04	**.31**	.00	.07
Protection measures item 1	.22	.06	-.15	.02	.06
Protection measures item 4	-.03	.10	.06	.08	-.05
Eigenvalues	4.38	1.43	1.34	1.32	1.04
Variance proportion	.23	.08	.07	.07	.05

Note. Factor loadings above .30 in bold

Table S5. Factor loadings of vulnerability indicators at T3

	Factor 1	Factor 2	Factor 3	Factor 4	Factor 5
Anxiety symptoms (GAD-2)	**.91**	-.08	-.09	.00	-.02
Depressive symptoms (PHQ-2)	**.88**	-.06	-.18	.10	.01
Overall Anxiety	**.70**	-.02	-.09	.06	-.01
Perceived stress (PSS-4)	**.69**	-.07	.18	-.04	-.03
Loneliness	**.64**	.06	.14	-.05	-.04
Psychosomatic complaints	**.60**	.01	.11	-.13	.00
Overall stress	**.51**	.11	**.39**	-.01	.04
Burdens	.27	-.02	.06	.00	.00
Aggression as victim	-.13	**.89**	.01	-.04	-.01
Aggression as perpetrator	-.04	**.81**	.10	-.07	.01
Pandemic-related fears	.17	.01	**.72**	-.03	.09
Pandemic-related behaviours	-.03	.12	**.40**	.05	-.03
Craving for internet activities	.15	-.04	-.03	**.72**	.00
Internet Use (CIUS-5)	-.10	-.03	.11	**.63**	.06
Alcohol consumption (AUDIT-C)	-.17	.00	.01	.03	**.88**
Alcohol loss of control	.10	.01	.03	.05	**.66**
News consumption	.00	-.03	.27	-.03	.03
Protection measures item 1	.11	.10	-.07	.05	.01
Protection measures item 4	-.03	-.01	.21	.11	-.05
Eigenvalues	4.30	1.37	1.29	1.21	1.03
Variance proportion	.25	.08	.08	.07	.06

Note. Factor loadings above .30 in bold

Table S6. Factor loadings of resilience indicators at T1

	Factor 1	Factor 2
Life satisfaction	**.80**	-.02
Optimism	**.73**	.01
Self-efficacy	**.64**	.04
Resistance	**.58**	-.03
Coping	.03	**.68**
Chance	-.03	**.67**
Eigenvalues	1.92	0.91
Variance proportion	0.32	0.15

Note. Factor loadings above .30 in bold

Table S7. Factor loadings of resilience indicators at T2

	Factor 1	Factor 2
Life satisfaction	**.79**	-.08
Optimism	**.76**	-.01
Self-efficacy	**.67**	-.01
Resistance	**.66**	.10
Coping	-.10	**.67**
Chance	.14	**.63**
Eigenvalues	2.12	0.86
Variance proportion	0.35	0.14

Note. Factor loadings above .30 in bold

Table S8. Factor loadings of resilience indicators at T3

	Factor 1	Factor 2
Life satisfaction	**.77**	.05
Optimism	**.76**	-.10
Self-efficacy	**.74**	.05
Resistance	**.69**	.06
Coping	.05	**.64**
Chance	-.04	**.64**
Eigenvalues	2.23	0.87
Variance proportion	0.37	0.15

Note. Factor loadings above .30 in bold

Table S9. Factor loadings of social cohesion items at T1

	Factor 1	Factor 2	Factor 3	Factor 4	Factor 5	Factor 6	Factor 7
Belonging family	-.05	.20	**.75**	.05	-.03	-.04	-.12
Belonging friends	-.07	.28	.06	**.70**	.00	.14	-.25
Belonging neighborhood	-.13	**.39**	.11	.08	**.57**	-.06	-.15
Belonging Berlin	-.03	**.83**	.00	.05	.01	.01	-.01
Belonging Germany	.06	**.92**	.06	-.10	-.06	.02	-.03
Belonging Europe	.07	**.97**	-.07	-.07	-.11	.04	.00
Belonging World	.04	**.86**	-.06	-.09	-.07	.02	.05
Trust family	.01	-.08	.74	.12	.01	-.13	-.06
Trust friends	.02	-.12	.04	**.74**	.12	.01	-.10
Trust neighbors	.05	-.08	.06	.15	**.69**	-.25	.01
Trust fellow citizens	.20	.11	.01	.13	.21	-.20	.24
Trust media	**.71**	.00	-.09	.08	-.03	-.06	.06
Trust police	**.56**	-.01	.13	-.10	.12	-.04	-.07
Trust senate	**.68**	.02	-.01	-.03	-.01	-.02	.07
Trust chancellor	**.85**	.01	.01	-.07	-.03	.04	.04
Trust government	**.91**	.01	.00	-.08	.01	.05	.02
Trust health system	**.63**	.03	.01	.03	.01	.07	-.11
Trust science	**.58**	.03	-.06	.12	-.10	.06	-.04
Interaction frequency family	-.02	-.04	**.61**	-.06	.01	.10	.05
Interaction quality family	.00	-.08	**.73**	.06	-.08	-.05	.09
Interaction frequency friends	-.03	-.02	-.07	**.66**	-.08	.30	.02
Interaction quality friends	.07	-.12	.09	**.64**	-.08	.10	.04
Interaction frequency others	-.09	.04	.04	.14	-.09	-.04	**.42**
Interaction quality others	.01	.01	.08	.10	-.04	-.16	**.42**
Prosocial frequency self -family	.03	.03	**.58**	-.10	.04	**.44**	.03
Prosocial frequency family - self	.08	-.03	**.56**	.06	-.05	**.44**	.02
Prosocial frequency self -friends	.04	.01	-.06	**.41**	.06	**.77**	-.05
Prosocial frequency friends - self	.07	-.05	-.07	**.51**	.08	**.68**	-.02
Prosocial frequency self -neighbors	-.02	-.08	-.07	-.11	**.79**	.28	.04
Prosocial frequency neighbors - self	.00	-.12	-.10	-.04	**.84**	.23	.03
Prosocial frequency self -others	.02	-.01	-.03	-.19	.06	.12	**.74**
Prosocial frequency others - self	.06	-.07	-.05	-.09	.05	.13	**.67**
Prosocial efforts	-.03	.06	.04	-.05	.06	.15	.10
Political participation	.01	.12	-.07	.14	.03	.05	.15
Social participation	-.05	-.02	.00	**.31**	.03	.12	.14
Eigenvalue	3.67	3.44	2.80	2.49	2.21	1.90	1.47
Variance proportion	.10	.10	.08	.07	.06	.05	.04

Note. Factor loadings above .30 in bold

Table S10. Factor loadings of social cohesion items at T2

	Factor 1	Factor 2	Factor 3	Factor 4	Factor 5	Factor 6	Factor 7
Belonging family	-.03	.28	**.64**	.07	-.06	-.05	-.24
Belonging friends	-.05	**.43**	.02	.04	.26	**.33**	-.24
Belonging neighborhood	-.14	**.46**	.02	**.53**	-.05	-.07	.08
Belonging Berlin	.00	**.86**	.00	.05	-.07	-.05	.09
Belonging Germany	.05	**.94**	.03	-.04	-.07	-.11	.06
Belonging Europe	.03	**.99**	-.04	-.08	-.02	-.12	.06
Belonging World	.00	**.92**	-.03	-.10	-.04	-.12	.09
Trust family	.01	-.01	**.58**	.18	.00	-.04	-.28
Trust friends	.05	-.03	.04	.27	**.33**	**.30**	**-.36**
Trust neighbors	-.02	-.06	.04	**.93**	-.13	-.08	.04
Trust fellow citizens	.18	.11	.01	**.46**	-.07	.11	.04
Trust media	**.78**	.02	-.06	-.01	.04	.00	.02
Trust police	**.59**	-.06	.12	.07	-.08	-.05	.01
Trust senate	**.71**	.04	.00	.01	-.04	.02	.06
Trust chancellor	**.90**	.01	-.01	-.06	.02	-.04	.07
Trust government	**.95**	.01	-.02	-.06	.01	-.05	.09
Trust health system	**.65**	-.03	.02	-.03	.03	.05	-.07
Trust science	**.62**	.03	-.05	-.03	.08	.02	-.03
Interaction frequency family	-.03	-.07	**.66**	-.07	-.19	.13	.12
Interaction quality family	.00	-.10	**.68**	.02	-.15	.23	.02
Interaction frequency friends	-.02	-.13	.02	-.15	.14	.74	.17
Interaction quality friends	.04	-.12	.01	.00	.18	**.69**	.02
Interaction frequency others	-.02	-.01	.03	.02	-.13	.13	**.32**
Interaction quality others	.07	.05	.00	.20	-.11	.12	.19
Prosocial frequency self -family	.04	-.02	**.66**	-.05	.27	-.25	.14
Prosocial frequency family - self	.01	-.01	**.58**	-.07	**.36**	-.08	.04
Prosocial frequency self -friends	.01	-.05	-.07	-.05	**.84**	.18	.10
Prosocial frequency friends - self	.02	-.06	-.09	.01	**.83**	.26	.04
Prosocial frequency self -neighbors	-.07	-.11	-.03	**.62**	.22	-.17	**.39**
Prosocial frequency neighbors - self	-.11	-.15	-.05	**.67**	.20	-.12	**.37**
Prosocial frequency self -others	.04	.07	-.04	.12	.14	-.02	**.57**
Prosocial frequency others - self	.09	.05	-.05	.13	.13	.01	**.53**
Prosocial efforts	.01	.01	.15	-.04	.14	-.03	.13
Political participation	-.02	.12	-.02	-.07	.11	.13	.18
Social participation	-.06	.02	.06	-.01	.02	.29	.21
Eigenvalue	4.01	3.88	2.50	2.38	2.20	1.54	1.65
Variance proportion	.11	.11	.07	.07	.06	.04	.05

Note. Factor loadings above .30 in bold

Table S11. Factor loadings of social cohesion items at T3

	Factor 1	Factor 2	Factor 3	Factor 4	Factor 5	Factor 6	Factor 7
Belonging family	-.02	.21	**.76**	.03	-.03	.03	-.10
Belonging friends	-.04	.28	.11	**.63**	.00	.08	-.10
Belonging neighborhood	-.14	**.50**	.05	.05	**.56**	-.05	-.08
Belonging Berlin	-.02	**.82**	.01	.05	.04	-.06	.06
Belonging Germany	.05	**.90**	.04	-.06	.00	-.02	.01
Belonging Europe	.05	**.97**	-.02	-.07	-.06	.03	-.04
Belonging World	.03	**.91**	-.03	-.09	-.08	.05	-.01
Trust family	.06	-.03	**.74**	.09	-.04	-.02	-.03
Trust friends	.11	-.07	.06	**.69**	.09	.08	-.08
Trust neighbors	.01	-.01	.03	.14	**.75**	-.17	.03
Trust fellow citizens	.17	.08	.05	.12	.27	-.21	.28
Trust media	**.79**	.03	-.07	.06	-.05	.03	.01
Trust police	**.54**	-.06	.13	-.08	.09	-.05	.01
Trust senate	**.73**	.05	-.03	-.02	-.02	-.02	.06
Trust chancellor	**.89**	.04	-.03	-.03	-.01	.08	-.05
Trust government	**.95**	.01	-.03	-.05	.01	.07	-.02
Trust health system	**.65**	-.01	.01	.07	-.06	.01	-.01
Trust science	**.66**	.02	-.07	.05	-.07	.05	-.03
Interaction frequency family	-.09	-.04	**.66**	-.04	-.05	.06	.09
Interaction quality family	-.04	-.07	**.76**	.13	-.08	-.02	.04
Interaction frequency friends	-.08	-.04	-.04	**.66**	-.07	.16	.10
Interaction quality friends	.03	-.12	.08	**.72**	.01	.05	.03
Interaction frequency others	-.05	-.01	.06	.04	-.05	-.09	**.50**
Interaction quality others	.07	-.03	.05	.08	.05	-.14	**.39**
Prosocial frequency self -family	.05	.02	**.60**	-.12	.04	**.52**	-.01
Prosocial frequency family - self	.04	-.02	**.58**	.02	.00	**.55**	-.01
Prosocial frequency self -friends	.03	.01	-.07	**.46**	.10	**.74**	-.07
Prosocial frequency friends - self	.08	-.06	-.08	**.53**	.14	**.72**	-.08
Prosocial frequency self -neighbors	-.06	-.05	-.11	-.05	**.84**	.31	.01
Prosocial frequency neighbors - self	-.05	-.08	-.13	-.05	**.88**	.28	.01
Prosocial frequency self -others	.00	-.01	-.03	-.18	.11	.21	**.70**
Prosocial frequency others - self	.07	-.04	-.04	-.15	.11	.17	**.67**
Prosocial efforts	.01	.02	.04	-.08	.04	.22	.13
Political participation	-.01	.12	-.10	.07	-.11	.14	.27
Social participation	-.08	-.01	.00	.20	-.05	.09	**.33**
Eigenvalue	4.05	3.70	2.90	2.56	2.55	2.14	1.69
Variance proportion	.12	.11	.08	.07	.07	.06	.05

Note. Factor loadings above .30 in bold
