# Supplementary material for: Coping with the COVID-19 Pandemic: Perceived Changes in Psychological Vulnerability, Resilience and Social Cohesion before, during and after Lockdown
Source: Int J Environ Res Public Health. 2022 Mar 10;19(6):3290. doi: 10.3390/ijerph19063290 (PMC8952288; doi:10.3390/ijerph19063290)
Supplement: Supplementary file 1 [file ijerph-19-03290-s001.zip › Supplement S4.pdf]

**Supplement S4**

**Table S12.** Factor loadings of social cohesion indicators on first-order and residual latent factors.

| first-order latent factor | indicator | factor loading |
|---|---|---|
| belonging | belonging family | .40 |
| | belonging friends | .64 |
| | belonging neighbors | .69 |
| | belonging Berlin | .76 |
| | belonging Germany | .68 |
| | belonging Europe | .54 |
| | belonging world | .46 |
| trust | trust family | .28 |
| | trust friends | .50 |
| | trust neighbors | .68 |
| | trust fellow citizens | .77 |
| | trust public media | .25 |
| | trust German police | .26 |
| | trust Berlin senate | .29 |
| | trust German chancellor | .21 |
| | trust German government | .25 |
| | trust German health system | .22 |
| | trust science | .21 |
| social interactions | frequency interaction family | .30 |
| | valence interaction family | .27 |
| | frequency interaction friends | .56 |
| | valence interaction friends | .44 |
| | frequency interaction others | .33 |
| | valence interaction others | .20 |
| social engagement | prosocial frequency self - family | .50 |
| | prosocial frequency family - self | .66 |
| | prosocial frequency self - friends | .43 |
| | prosocial frequency friends - self | .31 |
| | prosocial frequency self - neighbors | .51 |
| | prosocial frequency neighbors - self | .70 |
| | prosocial frequency self - others | .45 |
| | prosocial frequency others - self | .36 |
| family | belonging family | .71 |
| | trust family | .71 |
| | frequency interaction family | .59 |
| | valence interaction family | .70 |
| | prosocial frequency self - family | .59 |
| | prosocial frequency family - self | .54 |
| friends | belonging friends | .55 |
| | trust friends | .59 |
| | frequency interaction friends | .48 |
| | valence interaction friends | .56 |
| | prosocial frequency self - friends | .52 |

| | | |
|---|---|---|
| | prosocial frequency friends - self | .44 |
| neighbors | belonging neighbors | .50 |
| | trust neighbors | .52 |
| | prosocial frequency self - neighbors | .70 |
| | prosocial frequency neighbors - self | .67 |
| others | frequency interaction others | .37 |
| | valence interaction others | .30 |
| | prosocial frequency self - others | .61 |
| | prosocial frequency others - self | .65 |
| national & international | belonging Berlin | .35 |
| | belonging Germany | .51 |
| | belonging Europe | .78 |
| | belonging world | .71 |
| | trust fellow citizens | .07 |
| institutions | trust public media | .61 |
| | trust German police | .44 |
| | trust Berlin senate | .65 |
| | trust German chancellor | .85 |
| | trust German government | .88 |
| | trust German health system | .48 |
| | trust science | .47 |

*Note.* Factor loadings are standardized for an ease of interpretation.
